# Intra- and inter-individual cognitive variability in schizophrenia and bipolar spectrum disorder: an investigation across multiple cognitive domains

**DOI:** 10.1038/s41537-023-00414-4

**Published:** 2023-12-18

**Authors:** Beathe Haatveit, Lars T. Westlye, Anja Vaskinn, Camilla Bärthel Flaaten, Christine Mohn, Thomas Bjella, Linn Sofie Sæther, Kjetil Sundet, Ingrid Melle, Ole A. Andreassen, Dag Alnæs, Torill Ueland

**Affiliations:** 1 Norwegian Centre for Mental Disorders Research, Division of Mental Health and Addiction, Oslo University Hospital, Oslo, Norway; 2 Norwegian Centre for Mental Disorders Research, Institute of Clinical Medicine, University of Oslo, Oslo, Norway; 3 Department of Psychology, University of Oslo, Oslo, Norway; 4 K.G. Jebsen Centre for Neurodevelopmental Disorders, University of Oslo, Oslo, Norway; 5 Centre for Research and Education in Forensic Psychiatry, Oslo University Hospital, Oslo, Norway

**Keywords:** Psychiatric disorders, Human behaviour

## Abstract

There is substantial cognitive heterogeneity among patients with schizophrenia (SZ) and bipolar disorders (BD). More knowledge about the magnitude and clinical correlates of performance variability could improve our understanding of cognitive impairments. Using double generalized linear models (DGLMs) we investigated cognitive mean and variability differences between patients with SZ (*n* = 905) and BD spectrum disorders (*n* = 522), and healthy controls (HC, *n* = 1170) on twenty-two variables. The analysis revealed significant case-control differences on 90% of the variables. Compared to HC, patients showed larger intra-individual (within subject) variability across tests and larger inter-individual (between subject) variability in measures of fine-motor speed, mental processing speed, and inhibitory control (SZ and BD), and in verbal learning and memory and intellectual functioning (SZ). In SZ, we found that lager intra -and inter (on inhibitory control and speed functions) individual variability, was associated with lower functioning and more negative symptoms. Inter-individual variability on single measures of memory and intellectual function was additionally associated with disorganized and positive symptoms, and use of antidepressants. In BD, there were no within-subject associations with symptom severity. However, greater inter-individual variability (primarily on inhibitory control and speeded functions) was associated with lower functioning, more negative -and disorganized symptoms, earlier age at onset, longer duration of illness, and increased medication use. These results highlight larger individual differences in patients compared to controls on various cognitive domains. Further investigations of the causes and correlates of individual differences in cognitive function are warranted.

## Introduction

Cognitive dysfunction is a central feature of psychotic disorders. In schizophrenia (SZ), group-level performance within key cognitive domains has been reported to be 1-2 standard deviations (SD) below the healthy population^1^. The clinical relevance of cognitive functioning is considerable, and impaired cognition is associated with poor response to treatment, difficulties with social relationships and occupational functioning^2^. Patients with bipolar disorder (BD) show intermediate group level cognitive dysfunction compared to SZ and healthy controls^3^. Despite better performance compared to SZ, the pattern of cognitive profiles is similar and are associated with functional outcome also in BD^4^.

Cognitive deficits are often present from illness onset^5^ and remain relatively stable throughout the course of the illness^6^, implicating neurodevelopmental processes in both disorders^5^. Studies also show overlap in symptom dimensions^7^, genetic lability^8^, and brain morphology^9^, suggesting a continuum and common pathophysiological mechanisms across psychotic disorders. Still, the association between cognitive impairments and genetic risk appears to be somewhat different in the two disorders. While most risk alleles related to SZ also are associated with lower intelligence and neurodevelopmental processes, BD risk alleles are related to both higher and lower intelligence (although more to higher than lower)^10^.

Most individuals vary in their cognitive performance from one test to another, but also show some consistency in their responses. In SZ and BD, intra-individual responses on cognitive tasks are more variable, and mean differences are often accompanied by considerable inter-individual differences on the group level^11^. Previous studies have attempted to parse this cognitive variability by identifying patient sub-groups with differentially affected neurobiology and clinical and cognitive profiles^11–13^. Cognitive variability could reflect differential sensitivity to illness related processes, including clinical developmental paths and vulnerability to environmental factors and genetic susceptibility. Furthermore, cognitive domains show differential trajectories through the lifespan^14^. Complex executive functions, including fluid skills, show protracted development through early adulthood. Processing speed peaks in the early twenties and thereafter steadily declines. Working memory performance is relatively stable in the twenties and starts to decline in the early thirties, while inhibitory control declines from the mid-thirties. Other functions involving acquired knowledge (such as intellectual functioning) continue to improve into middle age and senescence. Consequently, the individual pattern of cognitive dysfunctions is likely to reflect the age period in which a patient experiences illness.

Neuroimaging has revealed case-control differences in several brain structural characteristics in severe mental disorders^15,16^, but the individual differences are substantial^17^ and the anatomical overlap is low^18^, suggesting that group-level differences may disguise considerable heterogeneity, with clinical relevance. Although substantial individual differences in cognitive function among patients with SZ and BD have been shown, there is a lack of studies simultaneously investigating differences within (intra-individual) and between individuals (inter-individual) on single tests. This is important since cognitive sub-domains showing homogeneous deficits could reflect common pathological features. Higher heterogeneity in patients on the other hand, could indicate disease subtypes or cognitive domains that are particularly sensitive to individual trajectories, and to illness and environmental perturbations^19^. Although previous studies often show lager standard deviations around the means on cognitive tests in SZ and BD compared to HC^3,20^, no one has explicitly tested the variability difference between these populations previously. Some tests or functions may be more sensitive to variability differences than others. Accordingly, quantifying performance variability is important to identify common mechanisms of cognitive dysfunction and to detect markers of cognitive subtypes^17,19^.

The aim of the current study was to test for mean and variability (intra -and inter-individual) differences between patients with SZ and BD spectrum disorders and HC within several cognitive domains. Intra-individual variability reflects the individual standard deviation of variation across cognitive tests. Inter-individual variability reflects the average deviation from the group-level predicted mean value or slope on a single test. Here the extent of variability in patients compared to HC can be interpreted as reflecting level of performance heterogeneity in the relevant cognitive domain. Using double generalized linear models (DGLM), which allows for simultaneous modeling of the mean and the variance, we investigated whether patients with severe mental disorders show higher or lower variability at the group-level compared to HC. Further, we investigated whether the magnitude of variability in cognitive performance is related to clinical severity, i.e. if the range of performance is associated with illness severity in the patient group.

Based on studies reviewed above^3,19^, we hypothesized that patients with SZ would show lower mean performance compared to HC but display larger variability. Based on Vaskinn and colleagues^12^, we anticipated a similar pattern but of smaller magnitude in BD when compared to HC. Since cognitive heterogeneity has been linked to clinical characteristics^12,21^, we further hypothesized that larger variability in performance among patients would be associated with higher clinical severity, age at onset and duration of illness.

## Results

### Demographic, clinical and cognitive characteristics

Sample characteristics are summarized in Table 1. In brief, patients with BD and HC were older than SZ patients. There was a higher proportion of females in the BD group and males in the SZ group. Furthermore, the HC group had significantly longer education and higher intellectual functioning compared to both patient groups, while BD patients had more education and higher intellectual functioning compared to the SZ group. As reported earlier in a subsample of the presented data (Vaskinn et al., 2020), patients with SZ presented with more positive, negative, disorganized and excited symptoms, worse psychosocial functioning (GAF-S,GAF-F, SFS) and reported higher drug use than patients with BD, while the BD group reported higher alcohol consumption than the SZ and HC groups.

**Table 1:** Sample characteristics.

Group	SZ spectrum	BD spectrum	HC	Statistic	Group comparison
Age	29.9 (9.5)	33.9 (11.8)	33.9 (9.6)	F = 47.2, *p* < 0.001	HC, BP > SZ
Gender (m/f)	530/375	208/314	612/558	χ^2^ (2, N = 2597) = -46.6, *p* < 0.001	SZ, BP ~ HC
Education (years)	12.4 (2.6)	13.6 (2.4)	14.4 (2.2)	F = 174.9, *p* < 0.001	HC > BP > SZ
WASI IQ	101.2 (14.3)	108.7 (12.3)	113.3 (10.7)	F = 247.9, *p* < 0.001	HC > BP > SZ
AUDIT	6.3 (6.5)	7.3 (6.4)	5.3 (3.2)	F = 17.8, *p* < 0.001	BP > SZ > HC
DUDIT	4.1 (7.6)	2.9 (6.1)	0.3 (1.2)	F = 85.9, *p* < 0.001	SZ > BP > HC
SFS	105.7 (9.2)	110.5 (9.2)	123.8 (5.2)	F = 1434, *p* < 0.001	HC > BP > SZ
PANSS negative	13.0 (5.8)	8.6 (3.5)	–	t = 15.6, *p* < 0.001	SZ > BD
PANSS positive	9.7 (4.1)	5.7 (2.5)	–	t = 20.3, *p* < 0.001	SZ > BD
PANSS disorganized	5.6 (2.5)	4.1 (1.5)	–	t = 12.4, *p* < 0.001	SZ > BD
PANSS Excited	5.6 (2.1)	5.2 (1.7)	–	t = 4.0, *p* < 0.001	SZ > BD
PANSS Depressed	8.1 (3.2)	7.7 (3.0)	–	t = 2.1, *p* = 0.040	SZ > BD
YMRS	4.6 (4.9)	3.2 (4.4)	–	t = 5.4, *p* < 0.001	SZ > BD
GAF Symptom	44.6 (12.2)	58.5 (11.3)	–	t = –21.2, *p* < 0.001	BD > SZ
GAF Function	45.6 (12.2)	56.5 (12.8)	–	t = –15.9, *p* < 0.001	BD > SZ
AAO	24.0 (8.2)	25.9 (9.7)	–	t = –3.9, *p* < 0.001	BD > SZ
AAO Depression	21.4 (8.0)	21.9 (9.2)	–	t = –0.9, *p* = 0.353	-
DOI	6.0 (6.9)	7.8 (9.2)	–	t = –4.1, *p* < 0.001	BD > SZ
Antipsychotics (*n*, %)	751 (83.0)	250 (47.9)	–	χ^2^ (1, *N* = 1427) = 194.7, (*p* < 0.001)	SZ > BD
- cumulative DDD	1.3 (0.9)	0.9 (0.8)	–	t = 6.9, *p* < 0.001	SZ > BD
Antiepileptics (*n*, %)	104 ((11.5)	201 (38.5)	–	χ^2^ (1, *N* = 1427) = 143.8, (*p* < 0.001)-	BD > SZ
- cumulative DDD	0.7 (0.5)	0.8 (0.5)	–	t = –1.7, *p* = 0.088	ns
Lithium (*n*, %)	19 (2.1)	94 (18.0)	–	χ^2^ (1, *N* = 1427) = 114.9, (*p* < 0.001)	BD > SZ
- cumulative DDD	0.9 (0.5)	1.1 (0.4)	–	t = –1.7, *p* = 0.091	ns
Antidepressants (*n*, %)	267 (29.5)	179 (34.3)	–	χ^2^ (1, *N* = 1427) = 3.5, (*p* = 0.066)	ns
- cumulative DDD	1.6 (1.1)	1.5 (1.0)	–	t = 1.1, *p* = 0.284	ns
Total medication use	1.6 (1.4)	1.4 (1.3)	–	t = 3.0 *p* = 0.002	SZ > BD
Psychotic episodes*	1 (1–50)	1 (0-25)	–	t(1) = 2.2, *p* = 0.136	ns
Manic episodes*	0 (0–22)	1 (0-85)	–	t(1) = 405.5, *p* < 0.001	BD > SZ
Hypomanic episodes*	0 (0–25)	1 (0-245)	*–*	t(1) = 481.5, *p* < 0.001	BD > SZ
Depressive episodes*	1 (0–101)	4 (0-120)	*–*	t(1) = 237, *p* < 0.001	BD > SZ

Presented are means, standard deviations (median, range for skewed data) and results from the pairwise comparisons.

*HC* healthy controls, *SZ* Schizophrenia, *BD* bipolar disorder, *WASI IQ* Wechsler Abbreviated Scale of Intelligence, *AUDIT/DUDIT* Alcohol/Drug Use Disorder Identification Tests, *SFS* Social Functioning Scale, *PANSS* Positive and Negative Syndrome Scale, *GAF* Global Assessment of Functioning scale, *YMRS* Young Mania Rating Scale, *AAO* age at onset, *DOI* duration of illness (years); *:retrospective count of lifetime episodes, *DDD* daily defined dosage.

### Mean and variability differences

**Fig. 1:**
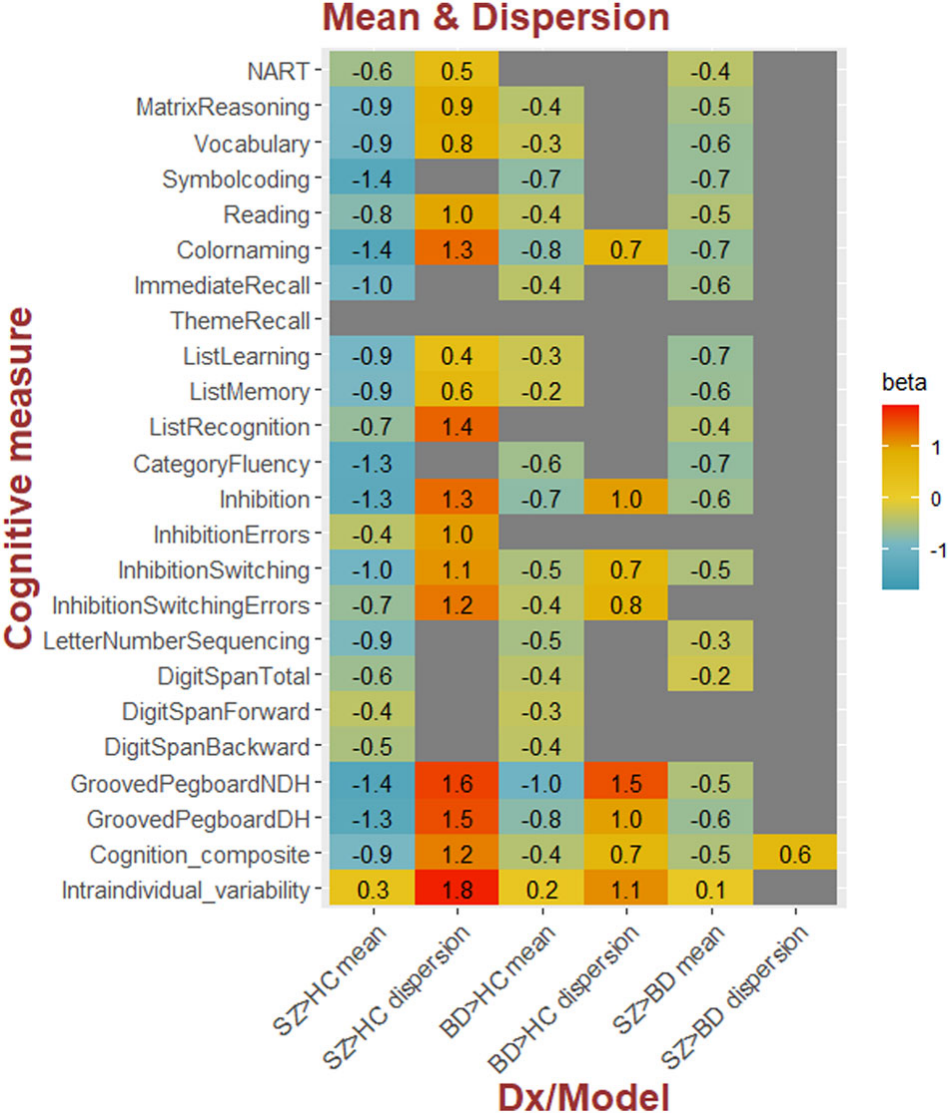
Mean and variability differences in cognitive performance. Presented are effect sizes (beta values, standardized regression coefficients) for significant (Bonferroni corrected (α = 0.05/48 tests = *p* < 0.000104)) mean and variability (dispersion) differences between groups across all cognitive tests, adjusted for age and sex. The more negative beta value the poorer the patient group performed compared to HC and more positive beta values indicate increased dispersion in patients compared to HC. Note, NDH Non-Dominant Hand, DH Dominant Hand, HC Healthy controls, SZ Schizophrenia, BD Bipolar disorder.

**Fig. 2:**
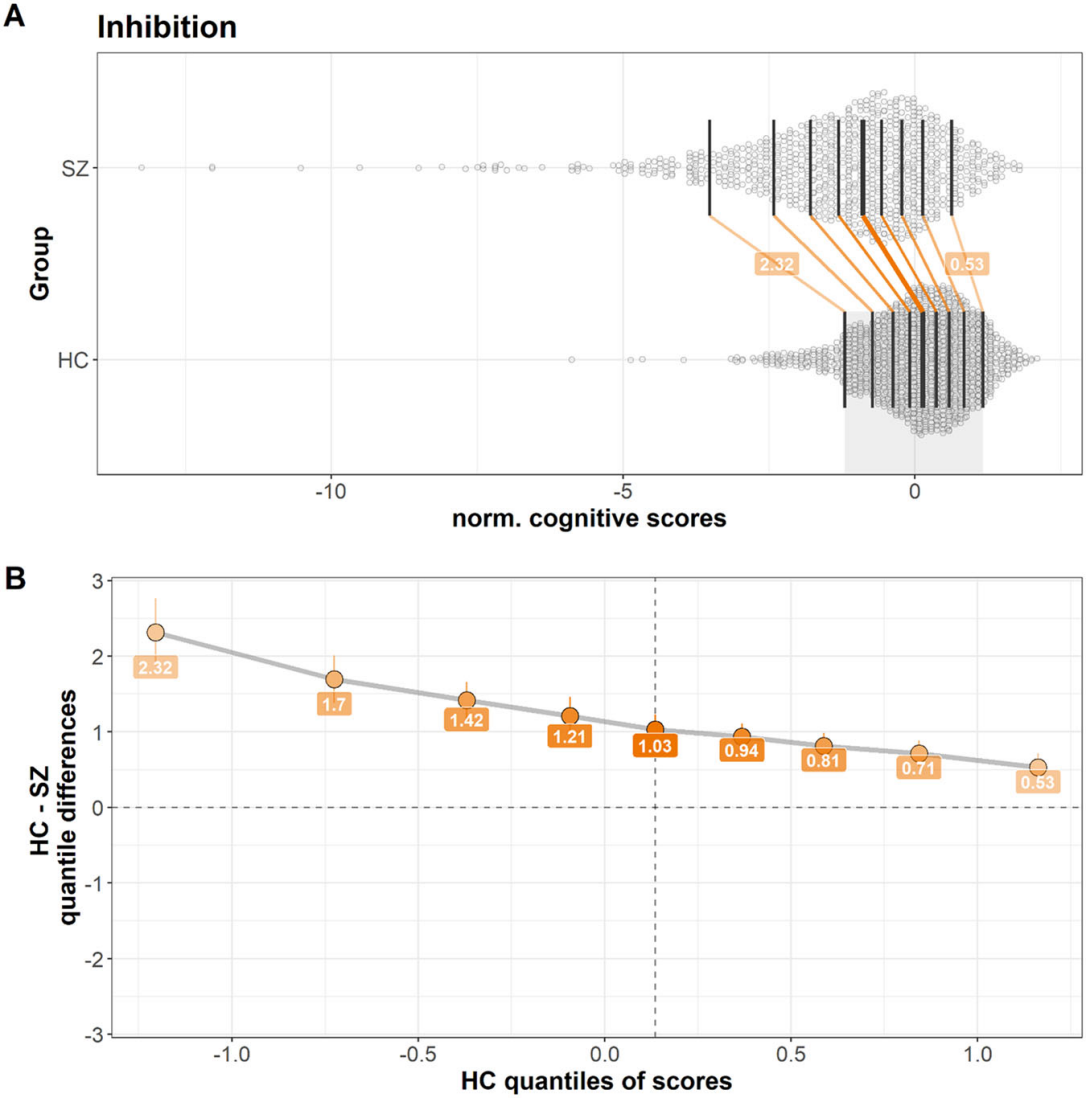
Shift function plots. **A** Marginal distributions of z-normalized cognitive scores in SZ and HC (adjusted for age and sex) for Inhibition (the remaining tests distributions are presented in the online supplement). The black vertical lines show deciles in each group, and the orange lines indicate decile-matched differences (shifts) between the two distributions. **B** Case-control difference plotted as a function of the distribution among HC. A sloped line indicates different dispersions for the two groups, beyond a simple mean shift. Number in the orange boxes indicate the case-control difference (z-scores) for the corresponding deciles within each group. The largest case-control differences are found in the lower deciles indicating a longer tail for lower performance individuals for patients compared to controls, with 4.4 times larger case-control difference in performance at the 10th percentile of the distribution for inhibitory control compared to the 90th percentile. SZ Schizophrenia, HC healthy controls.

Mean and variability (dispersion) differences for all groups are summarized in Table 2 and Fig. 1. Compared to HC, patients with SZ had significantly lower mean scores across most cognitive measures. Patients with SZ also had significantly larger intra-individual variability compared to HC. Furthermore, DGLM revealed significantly larger inter-individual variability among patients with SZ compared to HC on measures of intellectual functioning, mental processing speed, learning and memory, inhibitory control fine-motor speed, and the cognitive composite score (Fig. 2). Additionally, SZ displayed larger dispersion on the intra-individual variability score compared to HC, indicating increased between subject variability for SZ compared to HC, in the 90^th^ percentile (supplementary). The model revealed no significant inter-individual variability differences in psychomotor processing speed, semantic fluency, working memory, or on two measures of verbal learning and memory.

**Table 2:** Mean and dispersion values of cognitive performance.

Cognitive measures	SZ/HC-mean		SZ/HC-disp		BD/HC-mean		BD/HC-disp		SZ/BD-mean		SZ/BD-disp	
	*t*	log *p*	*t*	log *p*	*t*	log *p*	*t*	log *p*	*t*	log *p*	*t*	log *p*
Intellectual functioning												
NART	**–10.6**	**–24.7**	**4.9**	**6.0**	–3.2	–2.8	2.5	1.9	**–5.5**	**–7.3**	1.5	0.8
Matrix Reasoning	**–16.7**	**–58.9**	**7.3**	**12.5**	**–6.7**	**–10.5**	3.7	3.7	**–7.4**	**–12.7**	3.1	2.7
Vocabulary	**–18.2**	**–68.6**	**7.8**	**14.0**	**–5.3**	**–7.0**	3.1	2.7	**–9.7**	**–20.7**	3.2	2.8
Psychomotor processing												
Symbol coding	**–29.3**	**–162.1**	3.3	2.9	**–13.2**	**–37.8**	2.4	1.8	**–10.5**	**–24.2**	0.6	0.2
Mental processing												
Reading	**–12.8**	**–35.7**	**6.1**	**8.9**	**–5.7**	**–7.9**	3.0	2.5	**–5.8**	**–8.0**	2.2	1.6
Color naming	**–19.8**	**–80.5**	**10.0**	**22.5**	**–11.3**	**–28.0**	**4.7**	**5.6**	**–7.4**	**–12.7**	3.8	3.8
Learning and memory												
Immediate recall	**–18.8**	**–72.9**	2.0	1.3	**–6.6**	**–10.2**	1.9	1.2	**–8.8**	**–17.2**	–0.1	0.0
Thematic recall	–3.4	–3.2	2.9	2.4	0.1	0.0	1.6	1.0	–2.5	–1.9	1.2	0.6
List learning	**–18.3**	**–69.4**	**4.7**	**5.6**	**–4.6**	**–5.4**	2.2	1.6	**–10.2**	**–22.7**	1.6	0.9
List memory	**–16.4**	**–56.9**	**6.0**	**8.6**	**–4.5**	**–5.2**	1.5	0.9	**–9.8**	**–21.0**	3.7	3.6
List recognition	**–9.1**	**–18.8**	**6.5**	**10.1**	–3.6	–3.5	2.4	1.8	**–5.0**	**–6.1**	2.7	2.2
Semantic fluency												
Category fluency	**–25.9**	**–130.5**	2.4	1.8	**–9.8**	**–21.7**	2.9	2.5	**–10.6**	**–24.4**	–0.9	–0.4
Inhibitory control												
Inhibition	**–18.6**	**–71.9**	**10.2**	**23.3**	**–10.0**	**–22.3**	**6.8**	**11.0**	**–6.3**	**–9.3**	2.1	1.5
Inhibition errors	**–6.8**	**–10.9**	**5.2**	**6.7**	–2.7	–02.1	1.9	1.2	–3.3	–3.0	2.5	1.9
Inhibition switching	**–15.3**	**–49.6**	**7.7**	**13.8**	**–7.6**	**–13.3**	**4.3**	**4.7**	**–6.0**	**–8.6**	2.3	1.6
Switching errors	**–10.8**	**–25.6**	**7.8**	**14.1**	**–6.2**	**–9.2**	**4.2**	**4.6**	**–3.7**	**–3.7**	2.9	2.4
Working memory												
Letter Number Sequencing	**–18.8**	**–73.2**	0.0	0.0	**–10.1**	**–22.6**	–0.3	–0.1	**–5.6**	**–7.5**	0.4	0.1
Digit Span Total	**–13.2**	**–37.5**	-0.9	-0.4	**–7.8**	**–14.1**	–0.8	–0.4	**–4.4**	**–4.8**	–0.3	–0.1
Digit Span Forward	**–6.9**	**–11.2**	2.8	2.2	**–6.1**	**–8.8**	–0.2	–0.1	–1.3	–0.7	1.6	1.0
Digit Span Backward	**–11.0**	**–26.6**	-0.2	-0.1	**–7.1**	**–11.7**	–0.2	–0.1	–3.1	–2.7	–0.5	–0.2
Fine-motor speed												
Grooved Pegboard NDH	**–19.3**	**–76.7**	**10.6**	**24.8**	**–11.2**	**–27.8**	**8.6**	**16.7**	**–4.4**	**–4.9**	0.8	0.4
Grooved Pegboard DH	**–19.0**	**–74.1**	**10.1**	**22.8**	**–10.4**	**–24.0**	**5.8**	**8.2**	**–7.0**	**–11.3**	3.0	2.5
Cognitive composite	**–28.6**	**–156.2**	**11.3**	**28.3**	**–13.6**	**–39.8**	**5.3**	**6.8**	**–11.9**	**–30.3**	**4.1**	**4.4**
Intra-individual variability	**17.3**	**62.9**	**12.2**	**32.4**	**10.0**	**22.3**	**6.5**	**10.1**	**5.9**	**8.3**	3.6	3.5

Presented are t values and log *p* values for mean and dispersion (disp) model, SZ > HC, BD > HC and SZ > BD across all cognitive tests, adjusted for age and sex. Significant effects (corrected for multiple comparisons), (α = 0.005/48 tests ~ log10(p) = 3.98, or *p* < 0.000104) are presented in bold.

*NDH* Non-Dominant Hand, *DH* Dominant Hand, *HC* healthy controls, *SZ* Schizophrenia, *BD* bipolar disorder.

Compared to HC, patients with BD showed significantly lower mean scores on most measures with the exception of premorbid intellectual functioning and two measures of verbal learning and memory. Patients with BD also displayed significantly larger intra-individual variability compared to HC. Further, the model predicted significantly larger inter-individual variability among patients with BD compared to HC on one measure of mental processing speed, in inhibitory control, fine-motor speed, and on the cognitive composite score. There was also a significant difference in dispersion on the intra-individual variability score, where BD (like SZ), displayed larger between-subject variation at the higher end of within-subject variability compared to HC. There were no significant dispersion differences in intellectual functioning, psychomotor processing speed, verbal learning and memory, semantic fluency, or on any of the working memory measures.

Lastly, compared to BD, patients with SZ displayed significantly lower mean cognitive performance on all measures except one measure of verbal learning and inhibitory control, and two measures of working memory. Moreover, SZ had significantly larger intra-individual variability across tests compared to BD and larger inter-individual variability on the composite score. There were however no significant inter-individual variability differences on any of the other cognitive measures between these groups.

### Associations between symptoms and variability in cognitive performance

**Fig. 3:**
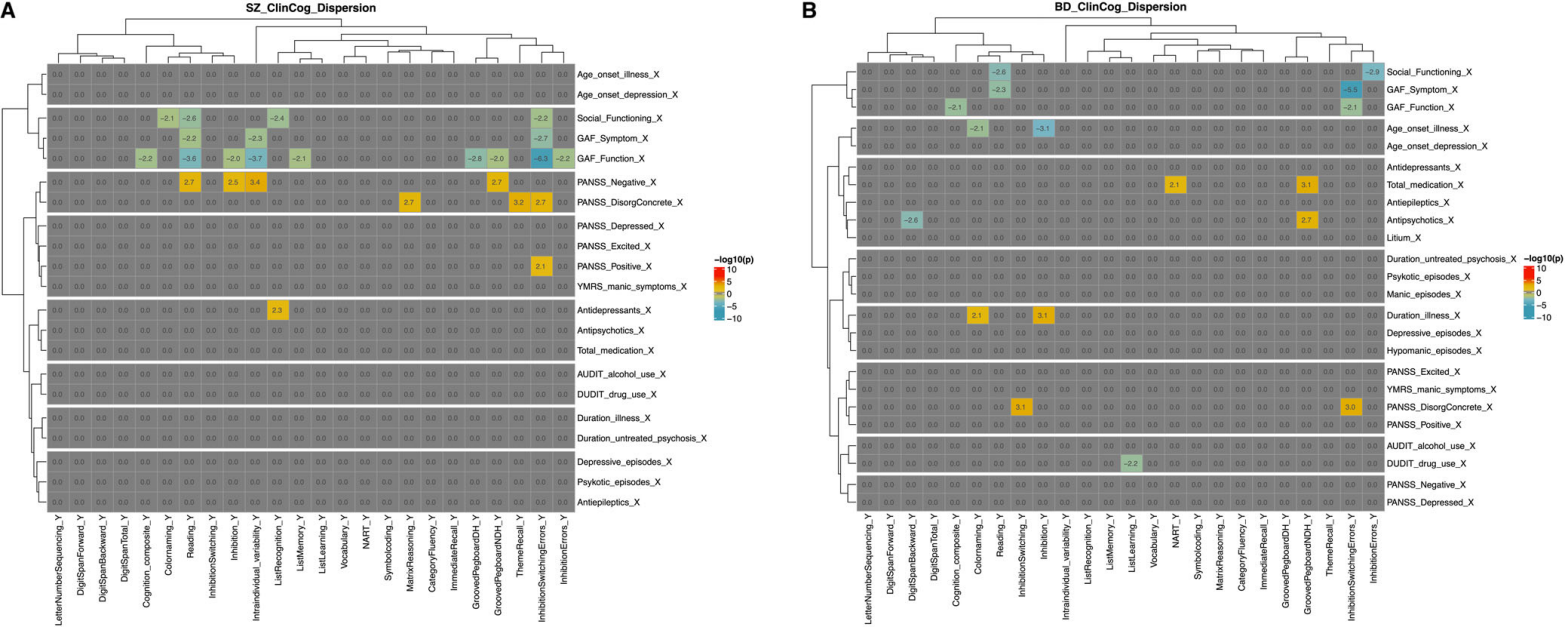
Associations between symptoms and variability in cognitive performance. Shows the associations between clinical characteristics and cognitive dispersion in schizophrenia (**A**) and bipolar (**B**) spectrum disorders, respectively. Each cell represents a DGLM dispersion effect, showing how clinical severity scores are associated with intra and inter-individual variability in cognition. Orange/red colors indicate positive associations and blue colors indicate negative associations. Note, AAO Age at onset, PANSS Positive and Negative Syndrome Scale, GAF Global Assessment of Functioning scale, YMRS Young Mania Rating Scale, DOI Duration of illness, DUP Duration of untreated psychosis, AUDIT/DUDIT Alcohol/Drug Use Disorder Identification Tests.

Figure 3A, B summarizes results from DGLMS testing for associations between clinical symptoms and intra -and inter-individual variability in test performance. Among patients with SZ, GAF-S and GAF-F scores were negatively associated and PANSS negative symptoms positively associated with intra-individual variability, indicating larger individual variation in test performance among patients with lower function and more negative symptoms. Regarding between subject variation on single domains/tests, GAF-S and GAF-F were similarly negatively associated with inter-individual variability in inhibitory control, GAF (S/F) and SFS were negatively associated with inter-individual variability in mental processing speed, while GAF-F was negatively associated with inter-individual variability on memory and fine-motor speed, also indicating larger between subject differences among patients with lower function on these particular domains. Furthermore, PANSS negative symptoms were positively associated with inter-individual variability in measures of inhibitory control, fine-motor speed, and mental processing speed, indicating higher more variable performance for these cognitive functions for patients with more negative symptoms. Lastly, disorganized symptoms were positively associated with inter-individual variability on measures of inhibitory control, memory and intellectual functioning, and the use of antidepressant medication was positively associated inter-variability in memory, indicating that increasing disorganized symptoms and use of antidepressant medication, are related to the larger variance found in these domains.

In summary, the results indicate larger variability (both within and between patients) in cognitive function among SZ patients with more severe compared to less severe clinical burden.

Among patients with BD, SFS and GAF (S/F) were negatively associated with inter-individual variability in measures of inhibitory control and mental processing speed. Additionally, GAF-F was negatively associated with overall cognitive functioning (cognitive composite). These results indicate that poorer social and global functioning is related to larger between-subject variability in these functions.

Furthermore, use of antipsychotic medications and total medication use were positively associated with inter-individual variability in fine-motor speed as well as in premorbid functioning, indicating that higher medication use is related to higher motor variability and more variable premorbid intellectual functioning. Also, there were negative associations between inter-individual variability in working memory and use of antipsychotic medications and between a measure of verbal learning and drug use, possibly reflecting that participants with higher drug use or using more antipsychotic medication, when considered jointly, are cognitively more homogeneous.

Regarding predicted variability on the inhibitory control function, disorganized symptoms and DOI were positively associated, and AAO was negatively associated with inter-individual variability in this function, indicating larger individual differences in inhibitory control among patients with early onset, longer duration of illness and more disorganized symptoms.

There were no associations between intra-individual variability in cognitive function and clinical symptoms.

## Discussion

The main finding of the present study was distinct patterns of significant mean and variability differences in cognitive domains in SZ and BD spectrum disorders compared to HC. We found larger intra-individual variability in patients with SZ and BD compared to HC, and in SZ compared to BD, when investigating the within-subject SD across cognitive performance scores. Further, we found larger inter-individual variability in several cognitive measures among patients with SZ and BD compared to HC. Specifically, we found larger inter-individual variability in measures of intellectual functioning, mental processing speed, verbal learning and memory, inhibitory control, and in fine-motor speed function, in SZ compared to HC, partly in agreement with our hypothesis. Among patients with BD, we found larger inter-variability in one measure of mental processing speed (color naming), in measures of inhibitory control and in fine-motor speed function compared to HC. There were no differences in inter-subject variability in any of the cognitive tests between the two patient groups. However, on the cognitive composite score patients with SZ had larger inter-individual variability compared to BD patients. We also replicated previous findings of lower mean cognitive scores across most tasks in SZ and BD compared to HC^3^, and in SZ compared to BD^5^.

Although, previous studies have shown that patients are less stable in their individual performance, to our knowledge, no studies have systematically investigated and shown that both within-subject and between-subject variability is higher among patients with SZ and BD compared to HC. This implies that the observed intra-individual variability in cognitive performance in patients coexists with a high degree of inter-individual variability on single constructs when compared to a healthy population. Nevertheless, this did not apply for all functions. There was no between-subject variability in working memory, semantic fluency or in psychomotor processing speed, in either patient group compared to HC.

The speeded component is a common element for the observed inter-variability in mental processing speed, inhibitory control and fine-motor speed in SZ and BD. The largest variability deviance appears to be in the lower end of the test-distributions, possibly pointing towards a disease subtype with specific speed challenges. Psychomotor slowing is frequently observed in SZ^22^, BD^23^, and across SZ and BD subgroups^12^, and is expressed as slowed initiation and execution of responses and movement. Psychomotor slowing overlaps with clinical features such as negative symptoms, catatonia and extrapyramidal symptoms^24^, and is a common side effect of some medications^25^. Psychomotor slowing is related to worsening in negative symptoms and disability and has been suggested as an independent predictor of functional outcome in BD and SZ spectrum disorders^26^. Thus, one might speculate that the observed variability on speeded tests is related to such clinical and functional characteristics. This was partly confirmed by our follow-up analysis, showing increased intra and inter-individual variability among patients (although most pronounced in SZ) with more negative symptoms and worse social and functional outcome. This implies that both within- and between-individual variability differences in speeded functions are related to similar clinical outcomes, which strengthen our findings on a single test level.

Furthermore, increased use of antipsychotic medication and total medication use were associated with increased variability in fine-motor speed in BD, assumingly reflecting individuals with a more severe type of illness. In SZ, there were no such associations. However, as > 83% of the SZ sample currently were taking antipsychotic medications it is difficult to parse out these effects, since medication use often follows the severity of illness.

During normal development, the speed in which one is able to name colors, words and visual objects has been shown to improve from childhood and adolescence to adulthood^27^. Following this, individual illness-related factors such as age at onset, duration of illness, and severity may impact these processes differentially, creating wider ranges of performance within patients than in healthy individuals. In BD earlier onset and longer duration of illness were related to increased variability in inhibitory control, potentially reflecting disrupted neurodevelopment in some patients. Previous studies have shown that poor inhibitory control is related to mania^28^, neuronal dysregulation^29^, and genetic liability^30^ in BD. Since cognitive controls functions develop into adulthood, they might be particularly vulnerable to late-maturational changes such as illness onset in BD, which include onset of manic symptoms.

Studies have reported heterogeneity in verbal memory^31^, intellectual functioning^11^, as well as in intellectual trajectories^12^ and “at risk” stages of psychotic disorders^32^. However, it is not clear whether this reflects individual sensitivity to illness-related mechanisms. We found increased variability primarily in SZ, indicating some underlying mechanisms magnifying individual differences in verbal learning and memory -and intellectual outcomes in this group. It has been speculated that SZ is a progressive neurodevelopmental disorder, and that cognitive reserve (higher premorbid intellect) is related to protective compensatory mechanisms^33^. Patients with BD showed no difference in a measure of premorbid intellectual functioning compared to HC, supporting normal-range levels of premorbid functioning^3^. Current intellectual functioning and verbal learning and memory were however affected in BD compared to HC, although not to the same extent as in SZ. Intellectual variability has previously been linked to symptom severity and different functional outcomes in SZ and BD^12^. In line with this, we found that larger variability in intellectual function was related to having more disorganized symptoms in SZ, and increased medication use in BD, potentially reflecting illness severity in both groups. Increased medication use is likely to reflect a more severe illness type and disorganized symptoms have been linked to severity in schizophrenia^34^.

In healthy neurodevelopment, processes involving acquired knowledge and crystallized intelligence develop into late adulthood^35^ and are continuously shaped by exposure to new information through education, work and social relationships. Thus, disrupted opportunities for social and cognitive maturation due to lower education and work achievements, and difficulties maintaining social relationships in SZ, could additionally be related to the increased variability reported here.

We did not find variability differences in working memory, semantic fluency or psychomotor processing speed. In these functions, the two patient groups seemed similarly affected when compared to HC, supporting a common disease mechanism^23^. However, we found that increased performance on these tests (as well as most others) predicts less between-subject variability and more homogeneous performance on speeded tests (supplementary material). This could indicate that general cognitive performance, including functioning in complex and compound functions such as semantic fluency and psychomotor processing speed, is important when predicting variability in speeded functions^23^. Further, overall performance (cognitive composite) was the strongest predictor for intra-individual variability in BD. In SZ there was no such association and measures of intellectual function were the strongest predictors (supplementary). Regardless, intra-individual variability predicts inter-individual variability in all other tests in all groups, except for working memory in BD (supplementary). Taken together these results suggest that variability differences in BD and SZ are overlapping but also different and influenced by diverse factors. Intra-individual variability seems to capture between-subject variability in single tests in a satisfactory manner. However, it may not be sensitive to capture relevant clinical information in better functioning patients, such as BD. Speed appears to be particularly sensitive to inter-individual variation, particular among subjects with more compromised functioning. Thus, identifying patients with specific speed challenges, beyond the general speed impairment found in the majority of patients is important to achieve personalized treatment.

### Strengths and weaknesses

There are some strengths and weaknesses in the study that warrant mentioning. This is the first study to explicitly investigate patient-control differences in cognitive variability. Further we present data from a large sample, covering multiple cognitive functions and clinical characteristics.

One study limitation is that we did not investigate single-task psychometrics. Cognitive tests likely differ in their sensitivity to individual differences at the extreme ends of the distribution, which could differentially affect our ability to detect diagnosis-based differences in inter-individual variability across cognitive tests. Thus, one possible confounder could be that the increased variance in patients reflects poor test psychometrics rather than “true” heterogeneity, as there were subjects performing more than 3 SD below the average. Therefore, we re-run all analyses with a cut-off of 3 SD to show that the above reported variability differences are not driven by extreme values (supplementary material). Nevertheless, these tests are commonly used when assessing heterogenous populations like SZ and BD, thus more knowledge about sensitivity to variability and how it relates to different clinical factors is important. Our result shows that individual variation in cognition relates to different clinical factors, and that the precision of a cognitive mean is dependent on symptoms, beyond simply covarying for them in a mean-model.

Environmental factors and experience may be important contributors to the observed variability in the patient groups. Unfortunately, we did not have data on environmental factors such as trauma, social deprivation, living situation etc., and this remains an important follow-up for future work investigating variability in these patients.

## Conclusions

Cognitive and clinical heterogeneity represents an important challenge and opportunity for research and for developing personalized treatments in mental health services. Here we extend previous reports by explicitly modeling the case-control variability-difference in addition to the average differences, by combining a case-control design with DGLM analyses. Further, we show that cognitive variability is related to clinical characteristics in patients, supporting the clinical relevance of cognitive diversity in patients.

Our results also indicate that intra-individual variability in patients coexists with a lot of inter-individual variability in single constructs. Consequently, it is important to differentiate the specific tests contributions from other causes of individual variability in future studies. Increased variability in measures with a speeded component, appears to be particularly clinically relevant, as these were associated with more negative symptoms and worse functional outcome. These results imply that “cognitive speed” should be a treatment target in BD and SZ subgroups with poor functioning^24^.

Furthermore, we find that younger age at onset and increased duration of illness were related to increased variability in inhibitory control in BD. This suggest that early treatment approaches, such as cognitive remediation, should be prioritized and evaluated for particularly impaired subgroups of younger BD patients within the early illness phase, to support the development of cognitive control functions.

## Methods

The cross-sectional study is part of the ongoing Thematically Organized Psychosis (TOP) study at the University of Oslo and Oslo University Hospital, approved by the Regional Committee for Medical Research Ethics and the Norwegian Data Protection Authority, and conducted in accordance with the Helsinki declaration.

### Sample

The sample included a total of 1427 patients (age range 18-65) recruited from psychiatric departments and outpatient clinics in Norway, primarily in the Oslo region. Diagnoses (Dx) were set by trained psychologists or physicians using the Structured Clinical Interview for DSM- IV axis 1 disorders (SCID- I)^36^ and included schizophrenia (*n* = 524), schizoaffective (*n* = 126), schizophreniform (*n* = 51), psychosis not otherwise specified (NOS, *n* = 204), bipolar I (*n* = 312), bipolar II (*n* = 182) and bipolar NOS (*n* = 28) disorders. The diagnoses were grouped into broad spectrum SZ (*n* = 905) and BD (*n* = 522) disorders. Patients recruited to the current study (both BD and SZ) were evaluated during a stable phase (e.g., patients that were too symptomatic were not able to participate). Healthy controls in the same age range were randomly invited (using national population records Statistics Norway) from the same catchment area (*n* = 1170) and screened for current or previous history of mental disorder before participation. All participants were recruited in the period between 2004 and 2019, informed about the study and provided written consent before participation. Study exclusion criteria were IQ below 70 (*n* = 64), previous severe head injury (requiring hospitalization including amnesia/loss of consciousness), or a neurological disease interfering with brain functioning.

### Clinical measures

Current positive, negative, disorganized, excited and depressive symptoms^37^ were assessed using the Positive and Negative Syndrome Scale (PANSS)^38^. Although PANSS primarily is used for assessing symptom severity in SZ, there are studies showing suitability for use in BD disorder samples^39,40^. Manic symptoms were assessed using the Young Mania Rating Scale (YMRS)^41^. Global functioning was assessed using the split version of the Global Assessment of Functioning scale (GAF)^42^, including separate scores for symptoms (GAF-S), and function (GAF-F). Social functioning was assessed using the Social Functioning Scale (SFS)^43,44^. Substance use was reported using the alcohol and drug use disorder identification tests (AUDIT/DUDIT)^45,46^. Medication use was reported using defined daily dose (DDD) of psychopharmacological treatment (including, antipsychotics, antidepressants, antiepileptics, lithium and total use) and estimated according to guidelines from the World Health Organization (https://www.whocc.no/atc_ddd_index). Age at onset (AAO) was calculated as the age of the first SCID-verified psychotic/manic/hypomanic episode. Additionally, we calculated AAO Depression, for first SCID-verified depressive episode. Duration of illness (DOI) was calculated subtracting AAO from current age. See Table 1 for clinical characteristics.

### Cognitive domains

Psychologists or personnel trained in standardized neuropsychological assessment administered the cognitive assessment. The following cognitive domains (*cursive*) were included (tests in **bold**): *Premorbid intellectual functioning* was assessed using the National Adult Reading Test (NART)^47^ and current *Intellectual functioning* using the **Matrix Reasoning** and **Vocabulary** subtests of the Wechsler Abbreviated Scale of Intelligence (WASI)^48^. *Processing speed* was sub-divided into *psychomotor processing speed* assessed using two different versions of the Digit-symbol-coding test (**Symbol coding**), from the Brief Assessment of Cognition in Schizophrenia (BACS)^49^ and the Wechsler Adult Intelligence Scale (WAIS-III)^50^ respectively, and *mental processing speed* (without a motor component), assessed using the **Color naming** and **Reading** subtests from the Color-Word Interference test, Delis Kaplan Executive Functioning System (D-KEEFS)^51^. *Verbal learning and memory* were assessed using the learning (**List Learning**), delayed recall (**List Recall**), and recognition (**List Recognition**) subtests of the Californian Verbal Learning Test (CVLT)^52^ or the Hopkins Verbal Learning Test (HVLT)^53^ and using the **Immediate Recall** and **Thematic Recall** sub-scores of the Logical Memory test from the Wechsler Memory Scale (WMS)^54^. *Semantic fluency* was measured using the **Category Fluency** subtest from the Verbal Fluency tests, D-KEEFS^51^ and or the MATRICS Consensus Cognitive Battery (MCCB)^55,56^. *Inhibitory control* was assessed using the **Inhibition,** **Inhibition Switching** and the accompanying error conditions from the Color-Word Interference Test, D-KEFS^51^. *Working memory* was measured using three sub-scores from the Digit Span test, including **Digit Span Forward,** **Digit Span Backward** and the **Digit Span Total** score from WAIS-III^50^, and using the total score from two different versions of the **Letter Number Sequencing** tests (MCCB and WAIS, respectively)^50,56^. Finally, to measure *fine-motor speed*, the two sub-scores from the Grooved Pegboard test, dominant hand (**Grooved Pegboard DH**) and non-dominant hand (**Grooved Pegboard NDH**) were used^57^. As some tests measuring the same skill were slightly different, we calculated standardized (not normalized) scores for each test, using the control group means and standard deviations as reference. Then, using standardized test scores, we calculated a cognitive composite score based on the average of the standardized scores and an intra-individual variance in performance score, using the standard deviations across the scores for each individual. A more detailed description of the tests (including descriptive statistics and number of valid cases per test) can be found in the online supplement.

### Statistical analyses

Statistical analyses were performed with R (version 3.5.3) and the R-package “dglm” (https://CRAN.R-project.org/package=dglm), and SPSS version 25.

Group differences in demographic and clinical data were assessed in SPSS using independent t-tests and analysis of variance (ANOVA) for continuous variables and chi- square statistics for categorical data.

In the main analyses we simultaneously estimated mean and between-subject variability (dispersion) parameters using DGLM, which iterates between the mean and dispersion sub-models until overall convergence. For each of the 24 cognitive variables, we used the following model specifications (1) Y ~ Age + Sex + Dx, (2) ~ Age + Sex + Dx. Here (1) is the mean model specification, and (2) is the dispersion model specification, and Y represents the cognitive outcome variables for the first GLM and the dispersion of the mean GLM (the residual scores) for the second GLM. For the group comparison we then obtain an estimate of the mean difference between groups, as well as an estimate of the difference in dispersion around the mean between SZ/HC, BD/HC, and SZ/BD for all the cognitive variables, including the precalculated composite and intra-individual variability scores.

All effects were corrected for multiple testing using the Bonferroni method (α = 0.005/48 tests ~ log10(p) =3.98, or *p* < 0.000104).

To investigate the degree to which the range of individual variability in cognitive performance is associated with disease severity within SZ or BD group, we added symptom measures (Sx) as additional independent variables in the dGLMs (Y ~ Age + Sex + Sx, (2) ~ Age + Sex + Sx). For these analyses we adjusted the false discovery rate^58^ using the R-function “p.adjust”, with q = 0.05 and method = ”BH”(https://www.jstor.org/stable/2346101), this was done across all tests and group-comparisons. The statistical threshold for q = 0.05 was set to log10(p) = 2.03, or *p* < 0.009.

Reanalysis of the mean and dispersion models with outlier removal (3 SD cut-off on all cognitive measures) can be found in the supplementary material (Fig. S2). There are also association analyses between mean performance on one test and the dispersion of the other (Fig. S3), to check whether performance on one task is related to cognitive heterogeneity in another, i.e. if higher performance on one test is related to higher or lower variability on another.

### Supplementary information

Online supplement

## Data Availability

The dataset used in the current study are not publicly available due to privacy or ethical restrictions. Anonymized data can be made available from the authors on reasonable request and with permission of Norwegian Center for Mental Disorders Research.
