# Supplementary material for: Intra- and inter-individual cognitive variability in schizophrenia and bipolar spectrum disorder: an investigation across multiple cognitive domains
Source: Schizophrenia (Heidelb). 2023 Dec 18;9(1):89. doi: 10.1038/s41537-023-00414-4 (PMC10728206; doi:10.1038/s41537-023-00414-4)
Supplement: Supplementary file 1 — Online supplement [file 41537_2023_414_MOESM1_ESM.pdf]

Supplementary materials

Intra- and inter-individual cognitive variability in schizophrenia and bipolar spectrum disorder: An investigation across multiple cognitive domains

Corresponding author:
Beathe Haatveit
email: beathe.haatveit@medisin.uio.no
Oslo University Hospital
Division Mental Health and Addiction
Psychosis Research Unit/TOP
PO Box 4956 Nydalen
0424 Oslo, Norway

Cognitive test batteries

In the current study participants were tested with either Test battery 1 (2004-2012) or Battery 2012 (ongoing). Some tests in the batteries are identical while others were slightly different versions that covered similar cognitive domains. The cognitive testing lasted for approximately 3-4 hours in total including breaks and patients were using their prescribed medications also on the day of testing. If participants reported being too tired, the testing was split over two days. After assessment, participants with invalid test results were removed from the analyses. This includes those who: misinterpreted the task instructions, exceeded the task stop criteria, had poor Norwegian language, and those with an IQ below a cut-off of 70. Below follows a detailed test-description of the cognitive assessments that were included in the study.

Grooved pegboard test: The execution of movement and motor dexterity is the main component of the task which requires visual and fine-motor coordination. The task consists of twenty-five holes with randomly positioned slots, and pegs that have a key along one side that fits the positioned slot. Pegs must be rotated to match the hole before they can be inserted. The total time to complete the pegboard for dominant hand and non-dominant hand respectively, are reported.

Letter–Number Sequencing test: Combinations of increasingly longer lists of intermingled numbers and letters are read aloud to the participant. The total number of correctly sorted sequences is reported.

Digit Span test: Increasingly longer sequences of digits are read aloud to the participant who is asked to repeat them back in the same order: **Digit Span Forward**, and thereafter in the reverse order: **Digit Span Backward**. The longest series of correct digits, forward and backward, are reported. Additionally, a **Digit Span Total score** including the total number of correct reported digits forward and backward summarized is reported.

Logical memory test measures **Immediate Recall** and **Theme Recall**: Individuals are read a short story and asked to recall as many details as possible. Number of recalled details is reported, additionally number of recalled theme details.

CVLT/HVLT: A list of 12 (HVLT) or 15 (CVLT) words from three (HVLT) or four (CVLT) different categories is orally presented. Participants are instructed to recall as many words as possible after each of three (HVLT) or five (CVLT) trials. The sum of correct repeated words is used as a measure of verbal learning: **List Learning**, the sum of correct repeated words after a 20-minute delay is used as a measure of verbal memory: **List memory**, and finally the sum of correct recognized word (yes/no questions) is used as a measure of cued recognition: **List Recognition**.

Verbal Fluency Test: In Category Fluency, a measure of semantic fluency, participants are asked to generate as many words as possible, from a given category (animals and boys names in the D-KEEFS version and animals in the MCCB version), within a time frame (60 seconds). The total number of words generated is reported.

Digit Symbol Coding test: The symbol coding test involves writing numbers (BACS) or nonsense symbols (WASI-III) corresponding to nonsense symbols (BACS) or numbers (WASI-III) as fast as possible for 90 (BACS) or120 (WASI-III) seconds. Scores are obtained for the correct number of symbols/numbers respectively coded.

Color-Word Interference test: In the first **Color naming** condition participant are asked to name the color of the ink (red, green, blue) of the written words as fast as possible. In the second **Reading** condition the participants are asked to read aloud the written words (red, green, blue) printed in black ink as fast as possible. In the third **Inhibition** condition the participants are asked to name the color of the ink (red, green, blue) of written words in incongruent colors as quickly as possible. Lastly, in the fourth **Inhibition Switching** condition, the participants are asked to name the color of the ink of written words in incongruent colors (as in the third condition), but to read the word aloud (and not name the ink color) whenever a word appears inside a box, as quickly as possible. For all four conditions the total time taken to complete the task is reported. Number of **Inhibition Errors** and number of **Inhibition Switching Errors** are additionally reported.

NART: NART is a measure of premorbid intelligence that consists of 50 orthographically irregular words that the participants are asked to read out loud. The total number of incorrectly pronounced words is reported.

Matrix Reasoning: The participants are asked to view an array of pictures with one missing. Then the participants are asked to select one picture that fits the array from five given options. Number of correct trails is reported.

Vocabulary: The participants are asked to define the words presented to them. Number of correct responses is reported.

Table S1. Frequency table: reported are n valid cases per test.

Test	HC	SZ	BD
**Vocabulary**	1168	884	515
**MatrixReasoning**	1169	892	515
**NART**	1050	646	421
**InhibitionSwitchingErrors**	1161	827	517
**InhibitionErrors**	1161	826	516
**InhibitionSwitching**	1136	826	516
**Inhibition**	1159	829	517
**Colornaming**	1162	830	518
**Reading**	1161	829	518
**Symbolcoding**	1169	900	520
**CategoryFluency**	1168	854	516
**ListRecognition**	1168	896	519
**ListMemory**	1168	826	516
**ListLearning**	1144	745	517
**ThemeRecall**	1090	705	493
**ImmediateRecall**	1090	705	493
**DigitSpanBackward**	1143	773	519
**DigitSpanForward**	1143	751	519
**DigitSpanTotal**	1143	752	518
**LetterNumberSequencing**	1144	820	456
**GroovedPegboardNDH**	1087	810	493
**GroovedPegboardDH**	1088	810	497

Table S2.

Cognitive measure	HC		SZ		BD	
	mean	SD	mean	SD	mean	SD
**Intellectual functioning**						
NART total errors	15.5	7.4	20.6	9.0	16.9	8.3
Matrix Reasoning	28.8	3.6	25.8	5.2	27.0	5.0
Vocabulary	62.7	7.2	54.7	10.3	60.4	8.5
**Psychomotor processing**						
WAIS Symbol coding	77.1	13.5	59.7	15.8	66.6	16.4
BACS Symbol coding	58.4	9.4	46.0	11.2	53.6	10.4
**Mental processing**						
Reading (seconds)	28.2	4.7	35.0	9.0	31.9	7.2
Color naming (seconds)	21.2	3.7	24.2	5.8	22.5	4.8
**Learning and memory**						
Immediate recall	27.8	6.4	21.6	7.1	25.6	7.2
Thematic recall	11.0	2.3	10.5	2.8	11.0	2.6
CVLT List learning	56.9	9.4	48.9	11.2	54.8	11.3
CVLT List memory	13.2	2.6	11.1	3.3	12.6	3.1
CVLT List recognition	14.2	2.5	13.0	3.6	13.6	3.5
HVLT List learning	28.6	4.0	24.4	5.4	27.7	4.3
HVLT List memory	10.3	1.7	8.4	2.6	9.9	1.9
HVLT List recognition	11.8	0.5	11.2	1.5	11.7	0.7

Table S2 Continues

**Inhibitory control**						
Inhibition (seconds)	48.6	9.8	61.3	18.6	56.2	16.8
Inhibition errors	1.1	1.4	1.7	2.4	1.3	1.8
Inhibition switching (seconds)	54.9	11.6	66.6	20.6	61.1	18.3
Inhibition switching errors	1.1	1.4	2.2	2.6	1.7	2.0
**Working memory**						
WAIS Letter Number Seq.	11.4	2.5	9.2	2.4	9.9	2.4
MX Letter Number Sequencing	15.6	2.9	13.2	3.0	14.3	2.9
WAIS Digit Span Total	16.6	3.7	14.5	3.6	15.1	3.5
WAIS Digit Span Forward	6.4	1.2	6.0	1.5	6.0	1.2
WAIS Digit Span Backward	5.0	1.3	4.4	1.3	4.6	1.3
**Semantic fluency**						
Fluency	48.9	8.3	38.0	9.7	43.8	10.5
MX Fluency	28.9	6.2	21.5	6.3	26.1	5.9
**Fine-motor speed**						
Grooved Pegboard NDH	67.0	10.4	81.9	22.6	79.5	23.4
Grooved Pegboard NDH	60.6	8.7	72.8	18.8	68.5	16.4

Presented are raw scores (means) and standard deviations for all cognitive measures (excluding the cognition composite and intra-individual variability score that were based on standardized scores). Some tests, measuring the same cognitive skill, had slightly different versions. These were combined into one unique z score. Note, HC: Healthy controls; SZ: Schizophrenia; BD: Bipolar disorder; SD: standard deviations; WAIS: Wechsler Adult Intelligence Scale; BACS: Brief Assessment of Cognition in Schizophrenia; MX: MATRICS Consensus Cognitive Battery; NDH: Non-Dominant Hand; DH: Dominant Hand.

Figure S1, a)

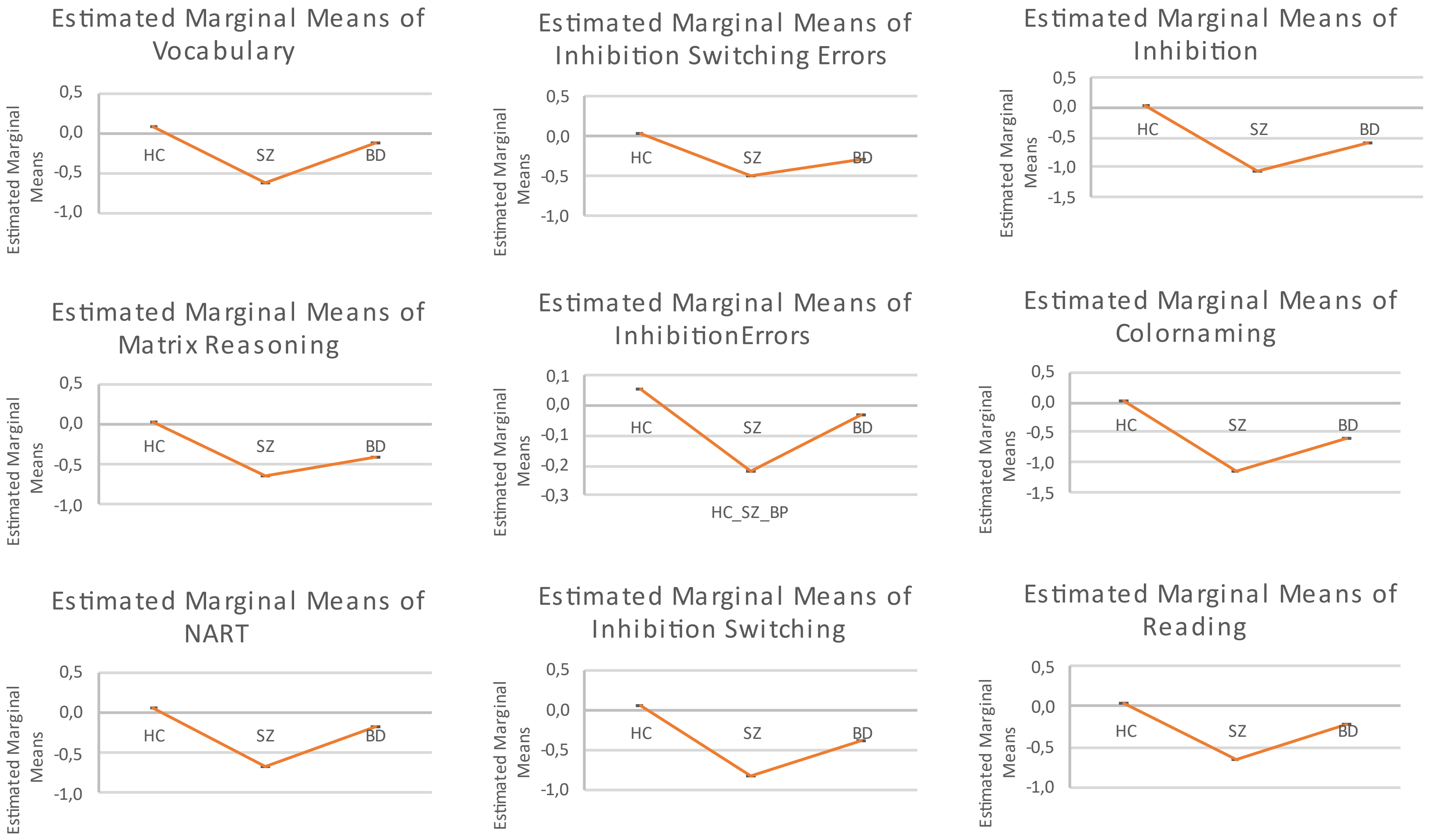
Estimated Marginal Means of Vocabulary
Estimated Marginal Means of Inhibition Switching Errors
Estimated Marginal Means of Inhibition
Estimated Marginal Means of Matrix Reasoning
Estimated Marginal Means of InhibitionErrors
Estimated Marginal Means of Colornaming
Estimated Marginal Means of NART
Estimated Marginal Means of Inhibition Switching
Estimated Marginal Means of Reading
Estimated Marginal Means
HC
SZ
BD
HC_SZ_BP
0,5
0,1
0,0
-0,1
-0,2
-0,3
-0,5
-1,0
-1,5

Figure S1, b)

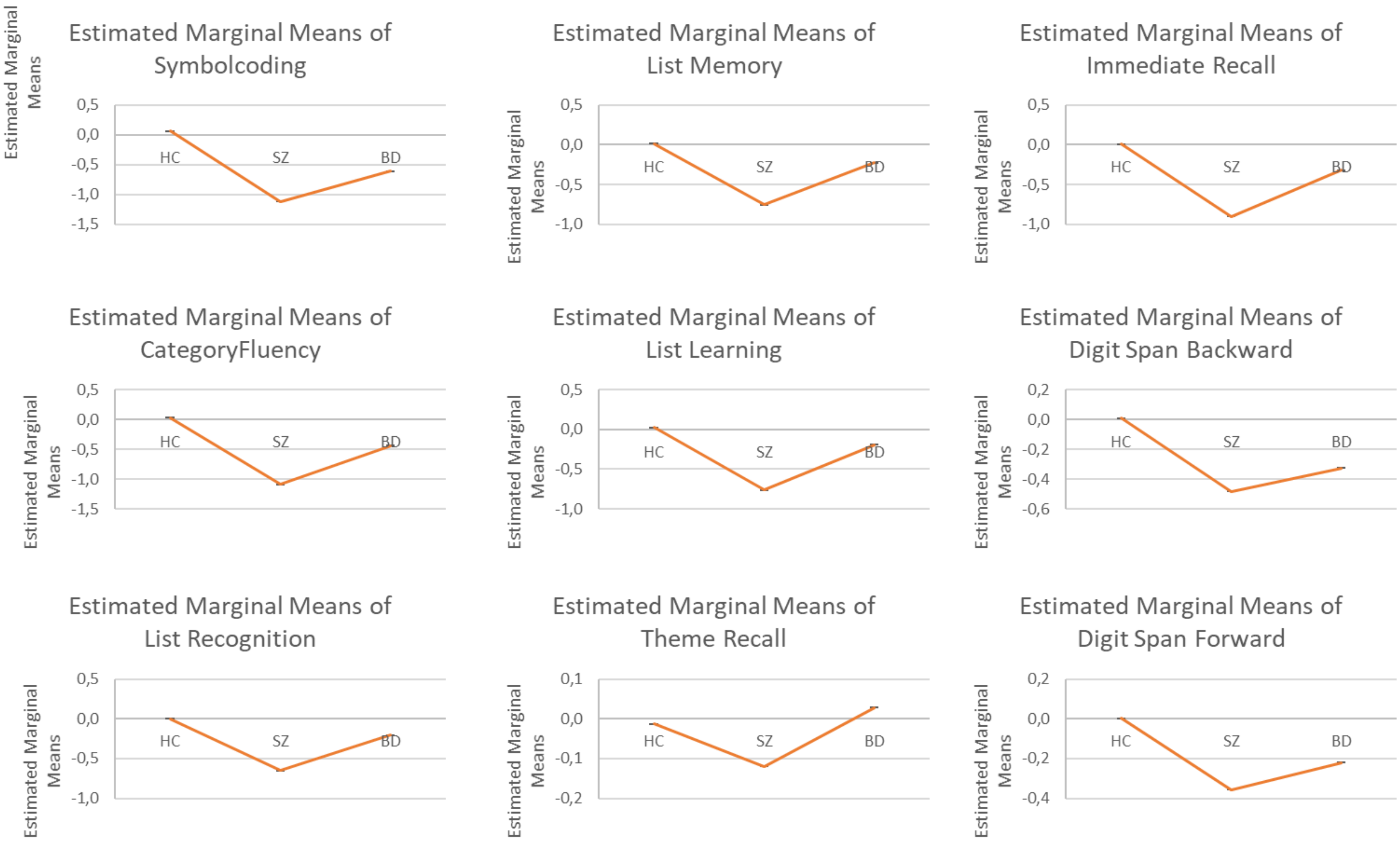
Estimated Marginal Means of Symbolcoding
Estimated Marginal Means
0,5
0,0
-0,5
-1,0
-1,5
HC
SZ
BD
Estimated Marginal Means of List Memory
Estimated Marginal Means
0,5
0,0
-0,5
-1,0
HC
SZ
BD
Estimated Marginal Means of Immediate Recall
Estimated Marginal Means
0,5
0,0
-0,5
-1,0
HC
SZ
BD
Estimated Marginal Means of CategoryFluency
Estimated Marginal Means
0,5
0,0
-0,5
-1,0
-1,5
HC
SZ
BD
Estimated Marginal Means of List Learning
Estimated Marginal Means
0,5
0,0
-0,5
-1,0
HC
SZ
BD
Estimated Marginal Means of Digit Span Backward
Estimated Marginal Means
0,2
0,0
-0,2
-0,4
-0,6
HC
SZ
BD
Estimated Marginal Means of List Recognition
Estimated Marginal Means
0,5
0,0
-0,5
-1,0
HC
SZ
BD
Estimated Marginal Means of Theme Recall
Estimated Marginal Means
0,1
0,0
-0,1
-0,2
HC
SZ
BD
Estimated Marginal Means of Digit Span Forward
Estimated Marginal Means
0,2
0,0
-0,2
-0,4
HC
SZ
BD

Figure S1, c)

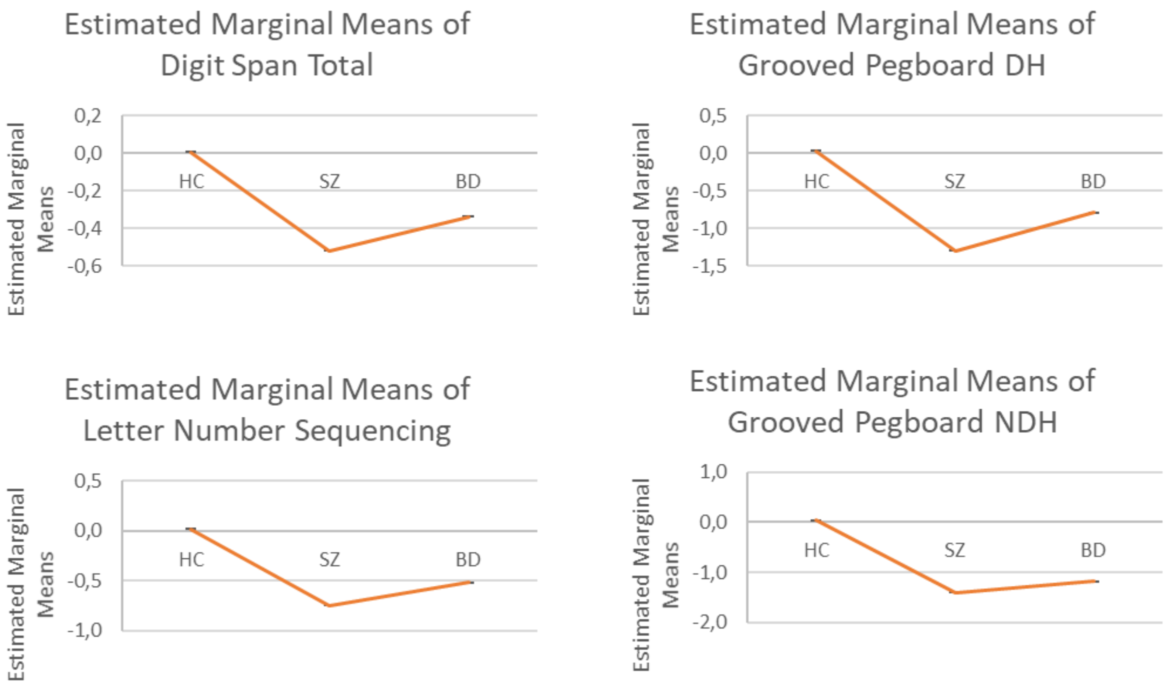

Figure S1 a, b, c) Presented are standardized scores for all cognitive measures in our test battery. Z scores were calculated using the control group means and standard deviations as reference. Note, HC: Healthy controls; SZ: Schizophrenia; BD: Bipolar disorder; NDH: Non-Dominant Hand; DH: Dominant Hand.

Shift function plots

To visualize the spread of tests scores we used the shift function plot (1, 4; R package: ggplot2). The shift function plots help us understand and quantify how two independent distributions differ from one another, by plotting the amount of shift between the two test distributions (or the quantiles of two distributions as a function of the quantiles of the healthy control group). However, this plot was not suitable for all measures as the test scores did not match the quantiles/deciles in the frequency distribution (probably due to that the test span was too narrow).

Overall, we see that there is a larger amount of shift in the lower part of the test distributions among patients compared to controls. This pattern is evident in both SZ and BD. Attachments (the shift function plots) are to be found in the end of the document.

Follow-up analyses with outlier removal

To rule out that the increased variance in the lower end of the test distributions in patients reflects poor test psychometrics rather than “true” heterogeneity, we re-run all analyses with 3 SD cut-off on all tests. The results are presented below (Figure S2). Briefly, we found similar patterns of mean and dispersion differences between patients with SZ and BD and healthy controls, additionally BD had larger inter-individual variability on one measure of intelligence and verbal memory that was not present in the full analyses. Moreover, we found dispersion differences between SZ and BD that was not present in the initial analyses. These results indicate that when removing extreme values, SZ has larger inter-individual variability on measures of intelligence, inhibitory control, verbal memory and on the intra-individual score compared to BD.

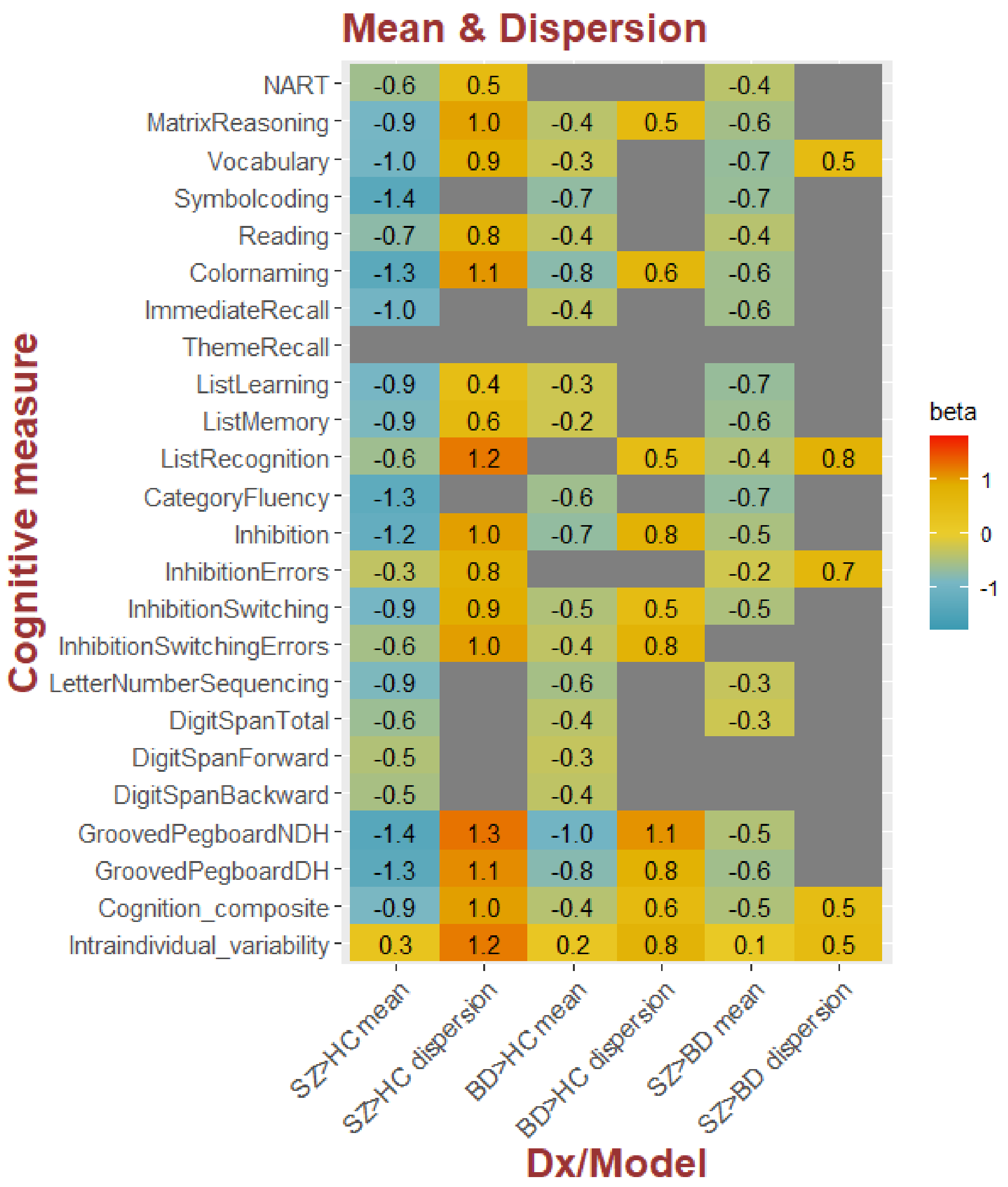

Figure S2 shows results from follow-up analyses with 3 SD outliers' removal on all cognitive measures. Presented are effect sizes (beta values, similar to standardized regression coefficients) for significant (Bonferroni corrected (α = 0.005/48 tests = $p<$ 0.00010415)) mean and dispersion differences between groups across all cognitive tests, adjusted for age and sex. The more negative beta value the poorer the patient group performed compared to HC and more positive beta values indicate increased dispersion in patients compared to HC. Note, NDH: Non-Dominant Hand; DH: Dominant Hand; HC: Healthy controls; SZ: Schizophrenia; BD: Bipolar Disorder

Association of mean cognitive performance on cognitive heterogeneity: Test performance x Test heterogeneity associations

To investigate whether cognitive performance on a given test is related to cognitive heterogeneity in others, we performed follow-up analyses within all subgroups. We used the mean performance on each test to see whether it is associated with heterogeneity of all other tests *(DGLM: (1) Y ~ Age + Sex + test, (2) Age + Sex + test)*. This was done separately for all groups and results are presented in Figure S3.

Briefly, we found that poorer functioning across multiple cognitive tests were associated with larger inter-individual variability on several speeded measures, including measures of inhibitory control, mental processing speed and fine-motor speed. This pattern was most pronounced in SZ, but also observed for BD as well as HC (Figure S3). A similar pattern was found for variability in verbal learning and memory, and intellectual functioning. We found that poorer performance on several complex tests (less influenced by speed) were associated with larger variability in intellectual functioning (particularly reasoning). This pattern was evident for HC as well as SZ, and less pronounced in BD. Additionally, we found that poorer performance on measures of psychomotor processing speed, intellectual functioning, and verbal learning and memory) were associated with larger variability on verbal learning and memory. The strongest negative association was found between measures verbal learning -and memory performance (list learning, list memory) and another variability measure of the same function (list recognition, this was most pronounced in HC), potentially indicating more homogenous performance among higher performing participants.

On most measures of working memory (and on some other tests), mean performance was positively associated with inter-individual variability in other measures of working memory, most pronounced in SZ. This indicates that participants performing poorer on working memory measures, when considered jointly, are cognitively more homogeneous than higher performing participants.

Regarding intra-individual variability, we found that the intra-individual variability score was positively associated with variability in all other functions, although not working memory function in BD. Overall cognitive functioning (cognition composite) was the strongest predictor for intra-individual variability in BD, indicating that poorer composite score predicted larger intra-individual variability. In SZ and HC there were no such association, and performance on verbal learning and memory, and intellectual function were among the strongest predictors for intra-

individual variability in SZ. In HC there were no associations between mean functioning on any cognitive measures and intra-individual variability.

Figure S3

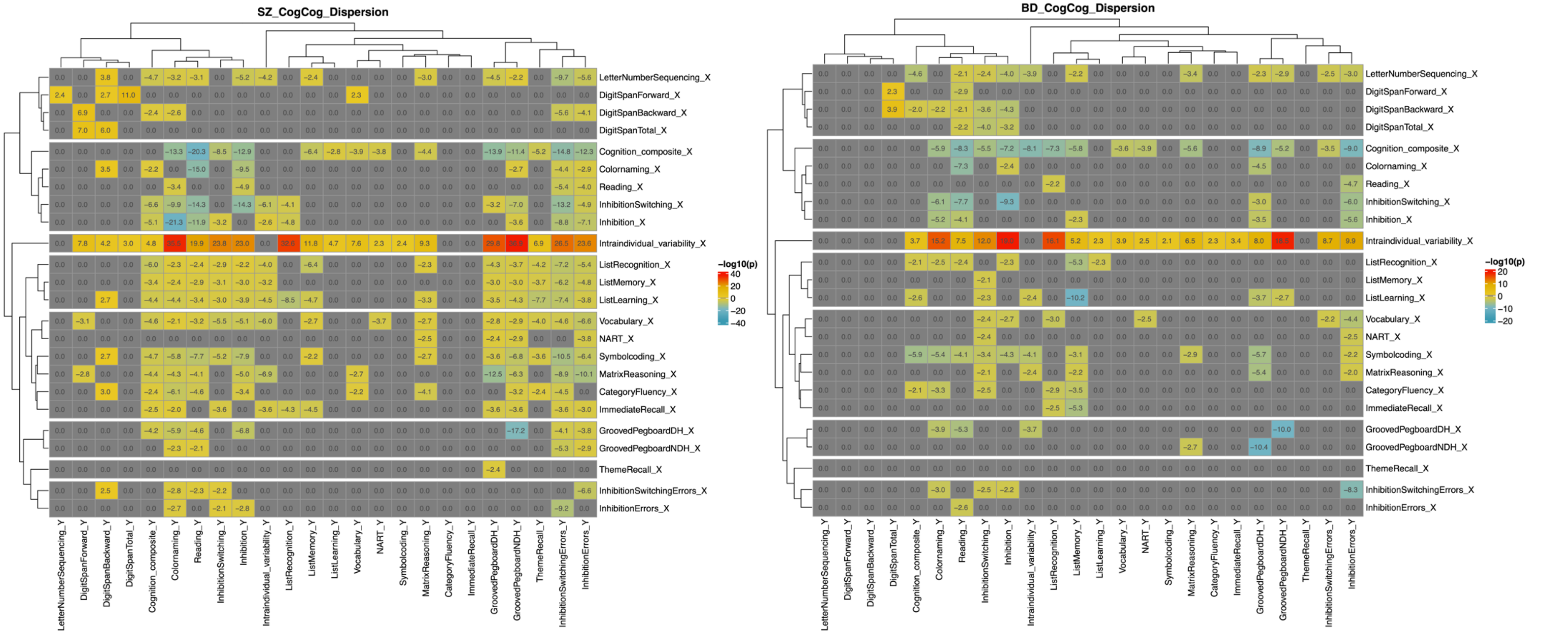

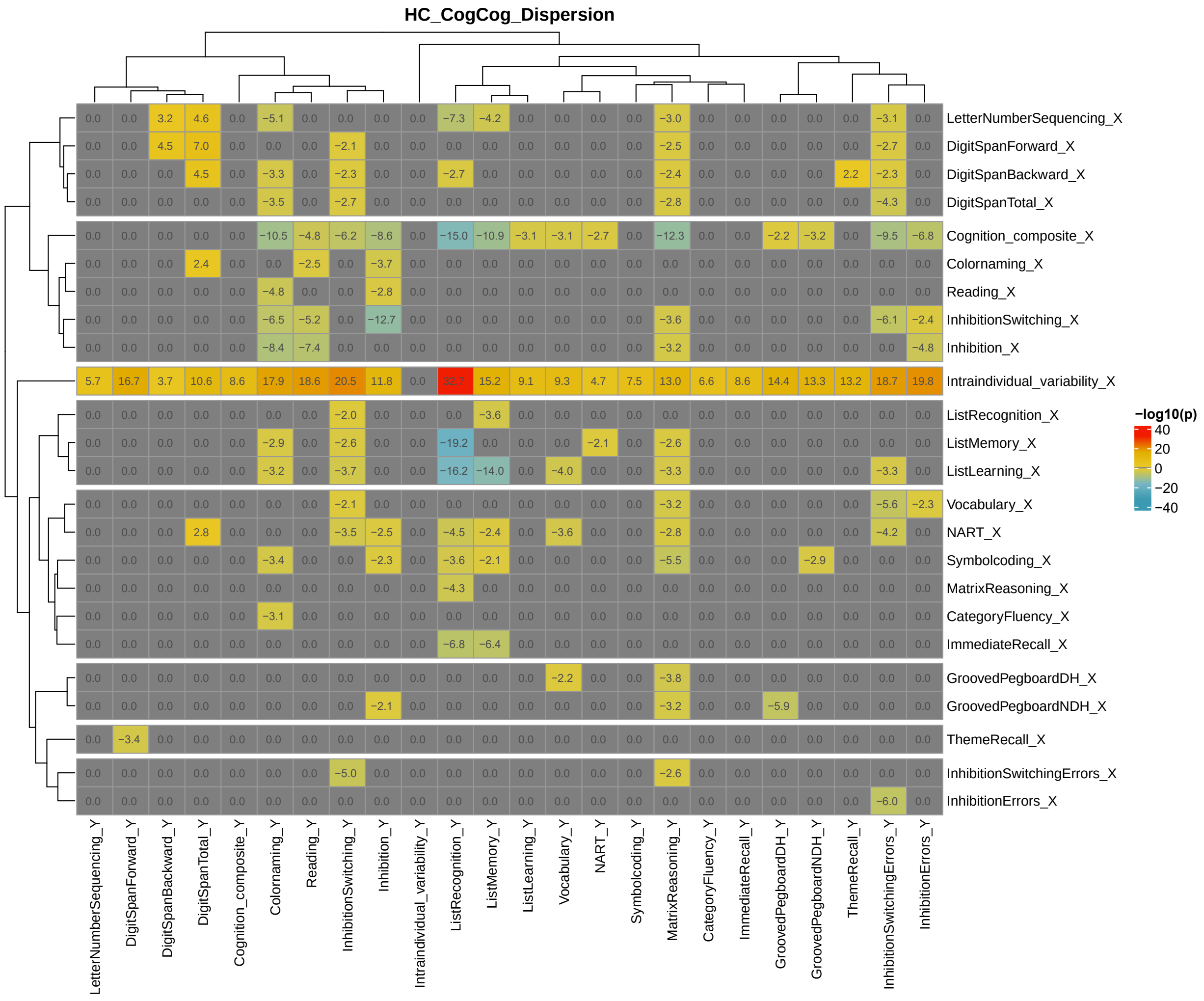

Figure S3. Shows the associations of performance for each of the cognitive scores with dispersion in performance on all other cognitive scores in schizophrenia (a), bipolar disorder (b), and healthy controls (c) using DGLMs. Each cell represents a DGLM dispersion effect, showing how the mean performance on one test is associated with variability on another test. The column names represent the predicted test (Y), and the row names represent the predictor test (X / X*Dx). Orange/red colors indicate positive associations and blue colors indicate negative associations. Values represent signed (by direction of association) -log10(p)-values.

References

1. Alnæs D, Kaufmann T, van der Meer D, Córdova-Palomera A, Rokicki J, Moberget T, et al. Brain Heterogeneity in Schizophrenia and Its Association With Polygenic RiskBrain Heterogeneity in Schizophrenia and Polygenic RiskBrain Heterogeneity in Schizophrenia and Polygenic Risk. JAMA Psychiatry. 2019;76(7):739-48.
2. Simonsen C, Sundet K, Vaskinn A, Birkenaes AB, Engh JA, Hansen CF, et al. Neurocognitive profiles in bipolar I and bipolar II disorder: differences in pattern and magnitude of dysfunction. Bipolar Disord. 2008;10(2):245-55.
3. Bora E. Neurocognitive features in clinical subgroups of bipolar disorder: A meta-analysis. Journal of Affective Disorders. 2018;229:125-34.
4. the shift function: a powerful tool to compare two entire distributions | basic statistics (wordpress.com)

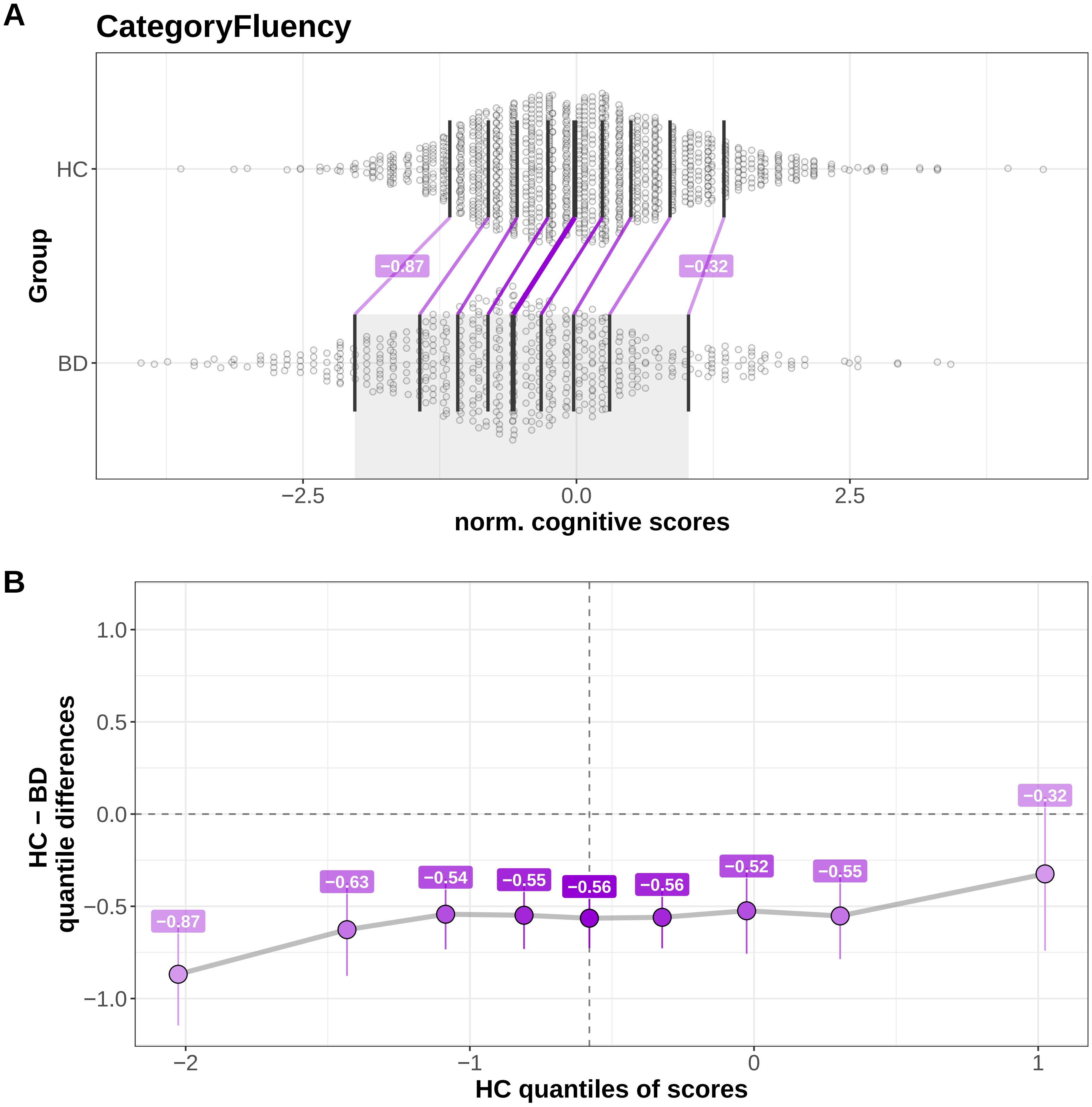

A
CategoryFluency
Group
HC
BD
−0.87
−0.32
−2.5
0.0
2.5
norm. cognitive scores
B
HC − BD
quantile differences
1.0
0.5
0.0
−0.5
−1.0
−0.87
−0.63
−0.54
−0.55
−0.56
−0.56
−0.52
−0.55
−0.32
−2
−1
0
1
HC quantiles of scores

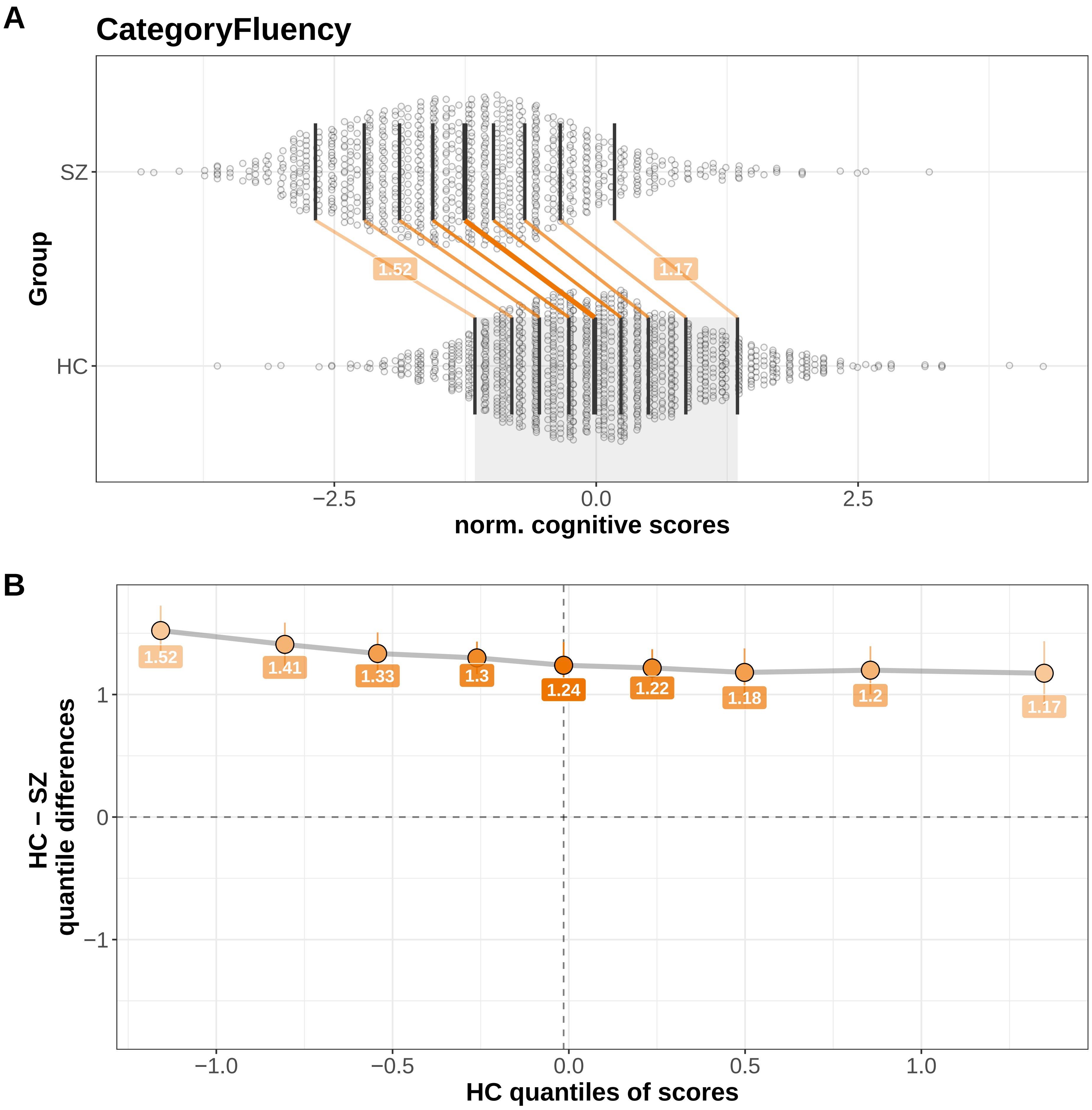
A
CategoryFluency
Group
SZ
HC
1.52
1.17
−2.5
0.0
2.5
norm. cognitive scores
B
HC – SZ
quantile differences
1.52
1.41
1.33
1.3
1.24
1.22
1.18
1.2
1.17
1
0
−1
−1.0
−0.5
0.0
0.5
1.0
HC quantiles of scores

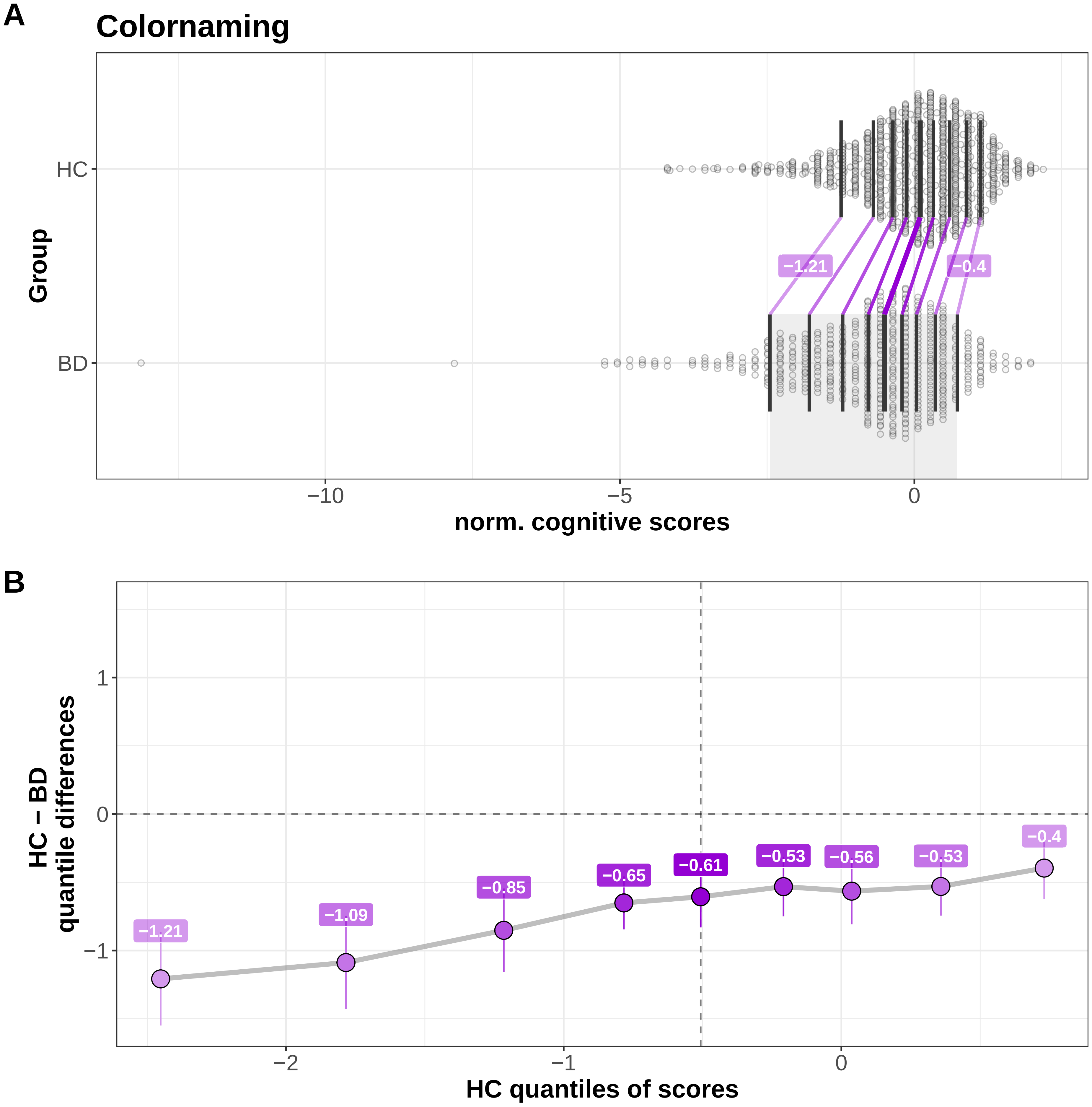
A
Colornaming
HC
BD
Group
−1.21
−0.4
−10
−5
0
norm. cognitive scores
B
HC − BD
quantile differences
1
0
−1
−1.21
−1.09
−0.85
−0.65
−0.61
−0.53
−0.56
−0.53
−0.4
−2
−1
0
HC quantiles of scores

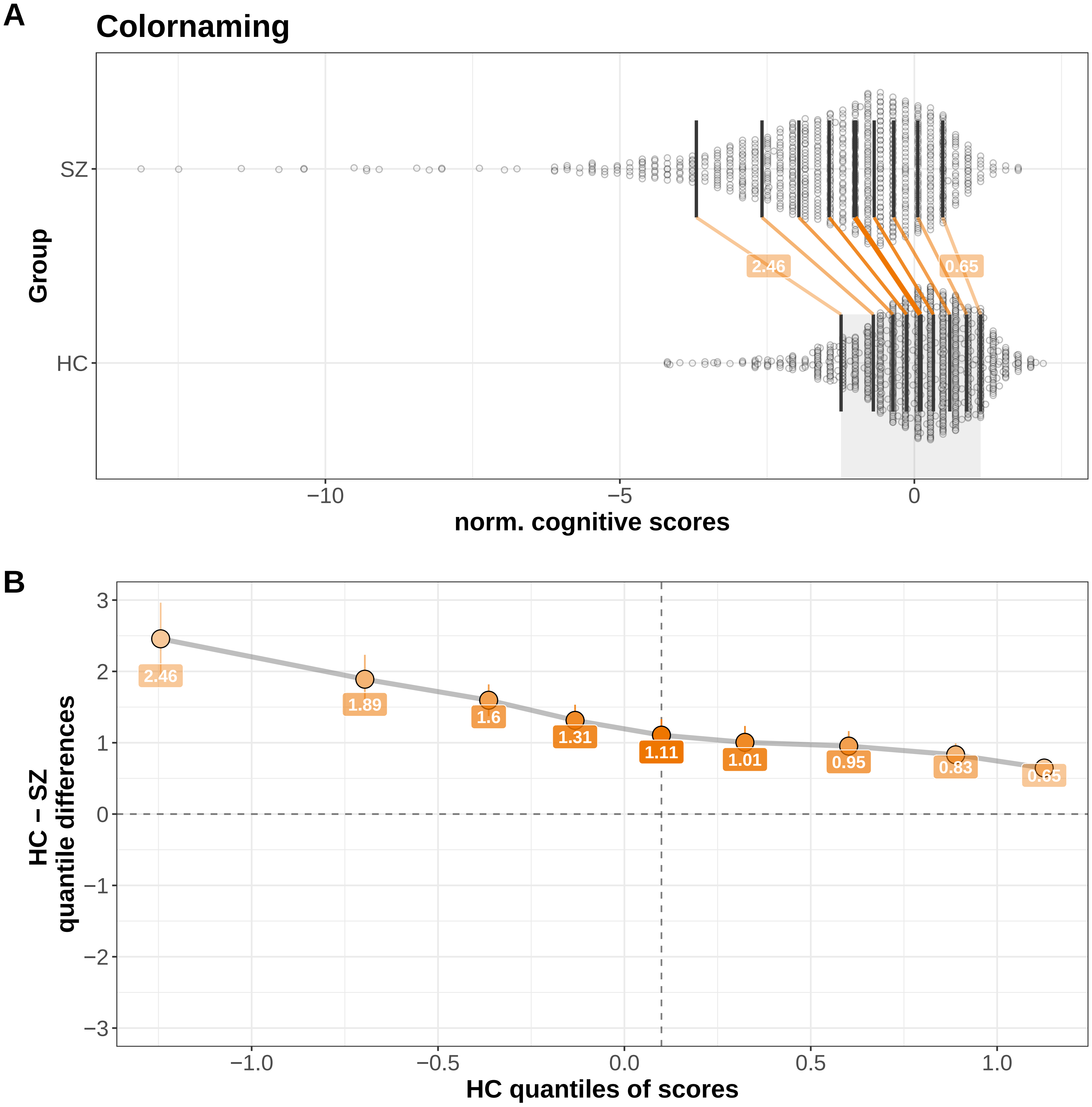

A
Colornaming
SZ
HC
Group
2.46
0.65
−10
−5
0
norm. cognitive scores
B
3
2
1
0
−1
−2
−3
2.46
1.89
1.6
1.31
1.11
1.01
0.95
0.83
0.65
HC − SZ
quantile differences
−1.0
−0.5
0.0
0.5
1.0
HC quantiles of scores

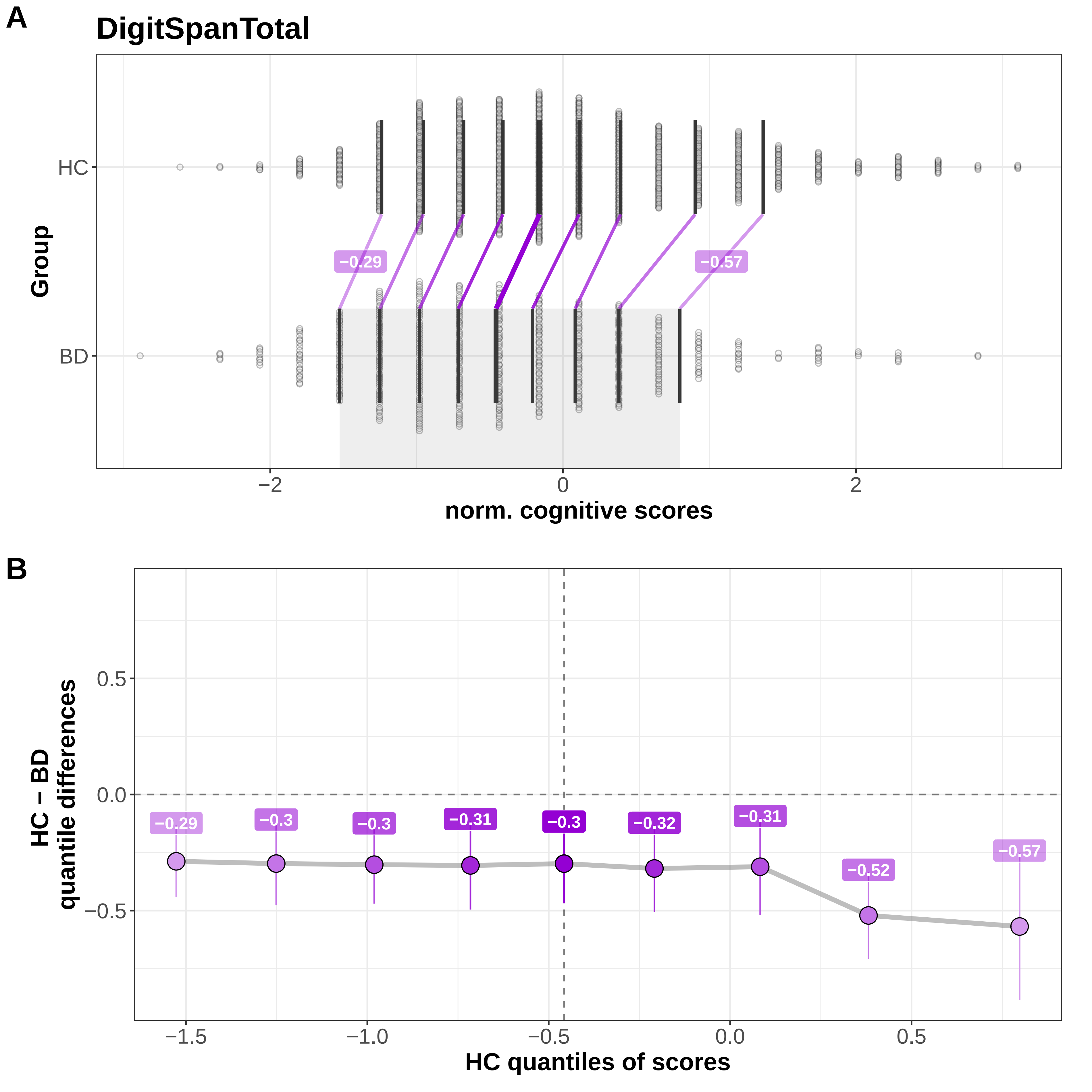

A
DigitSpanTotal
Group
HC
BD
−0.29
−0.57
−2
0
2
norm. cognitive scores
B
HC – BD
quantile differences
0.5
0.0
−0.5
−0.29
−0.3
−0.3
−0.31
−0.3
−0.32
−0.31
−0.52
−0.57
−1.5
−1.0
−0.5
0.0
0.5
HC quantiles of scores

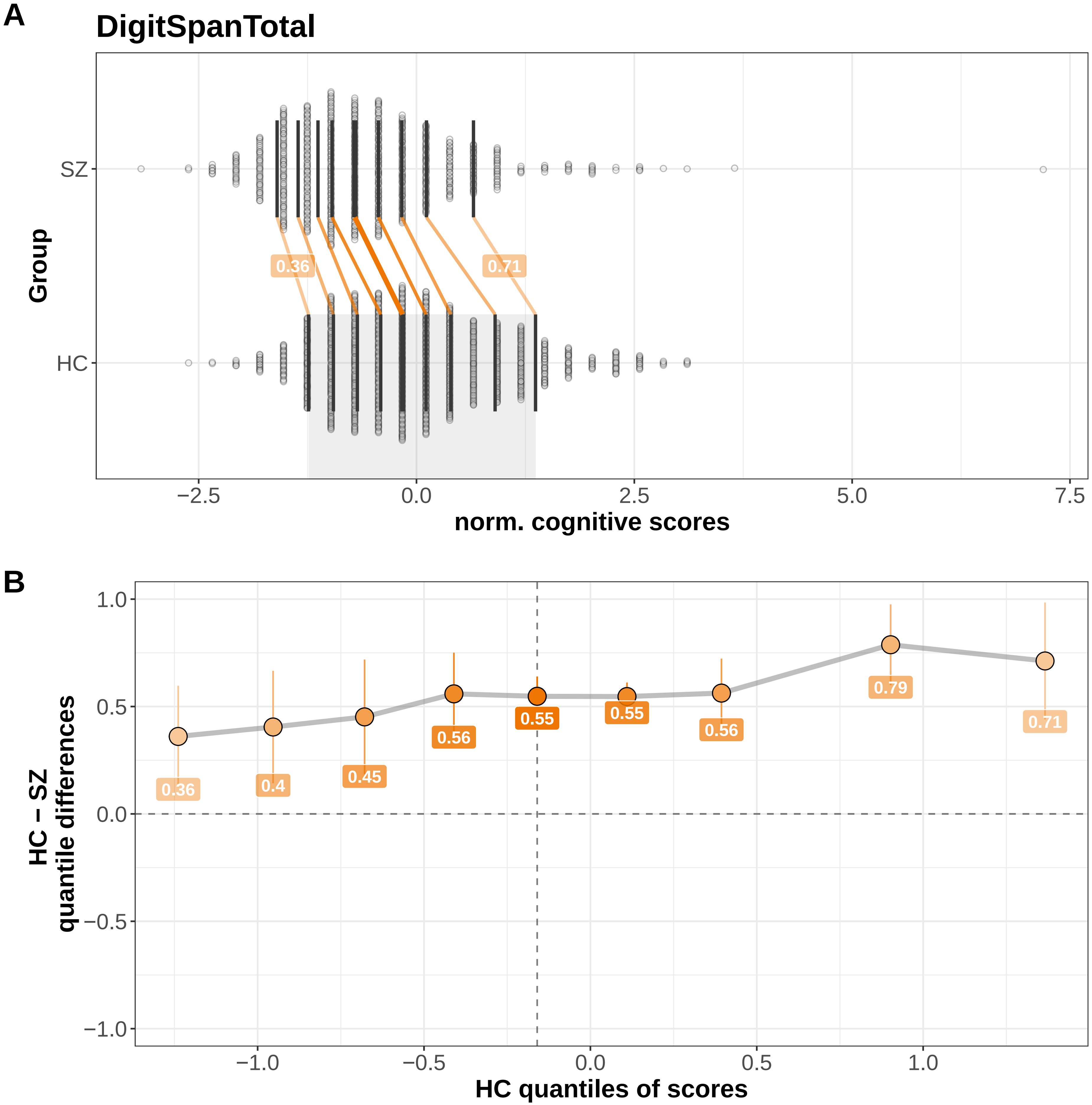
A
DigitSpanTotal
SZ
HC
Group
0.36
0.71
−2.5
0.0
2.5
5.0
7.5
norm. cognitive scores
B
HC − SZ
quantile differences
1.0
0.5
0.0
−0.5
−1.0
0.36
0.4
0.45
0.56
0.55
0.55
0.56
0.79
0.71
−1.0
−0.5
0.0
0.5
1.0
HC quantiles of scores

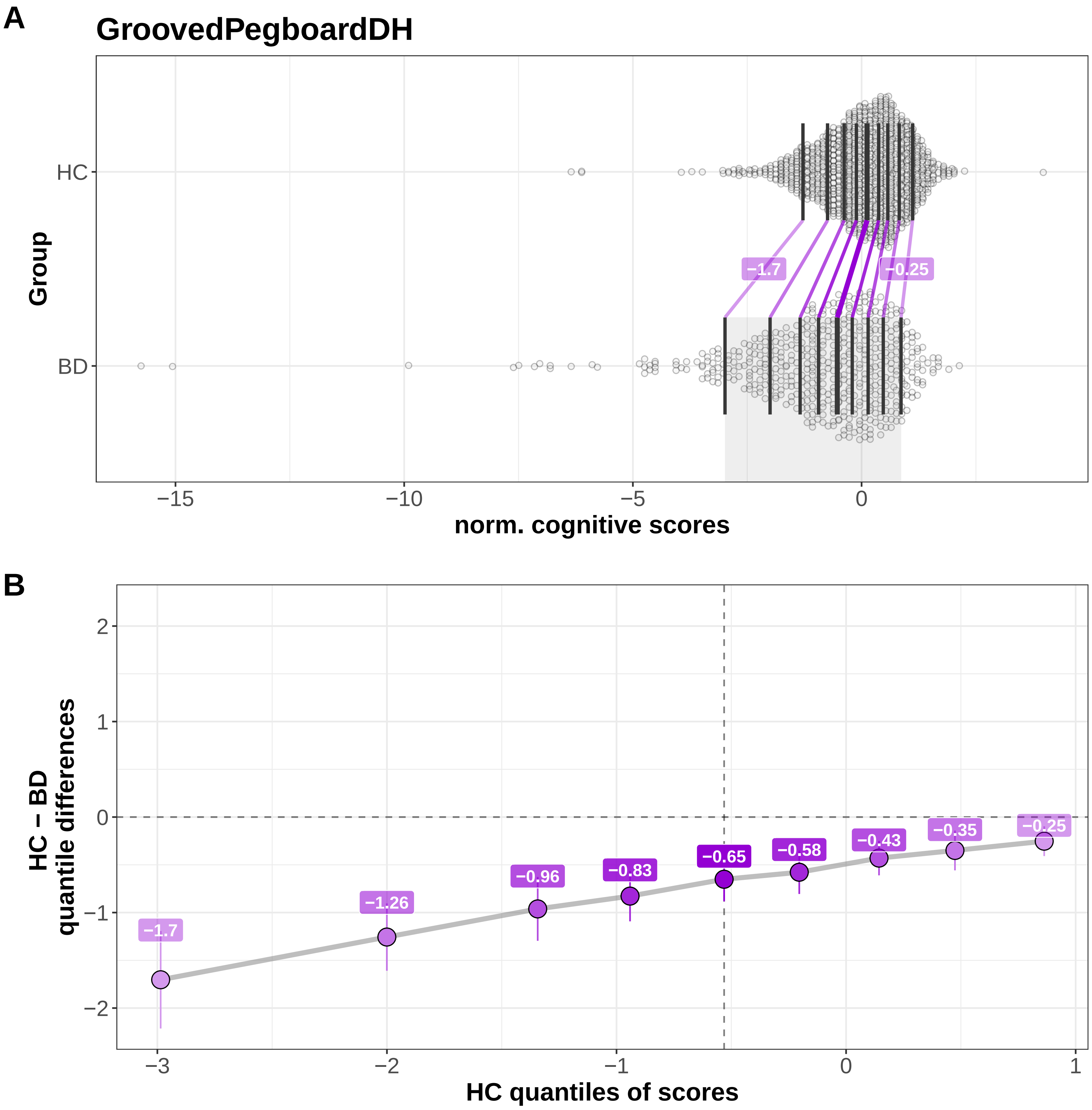

A
GroovedPegboardDH
HC
BD
Group
−1.7
−0.25
−15
−10
−5
0
norm. cognitive scores
B
HC – BD
quantile differences
2
1
0
−1
−2
−1.7
−1.26
−0.96
−0.83
−0.65
−0.58
−0.43
−0.35
−0.25
−3
−2
−1
0
1
HC quantiles of scores

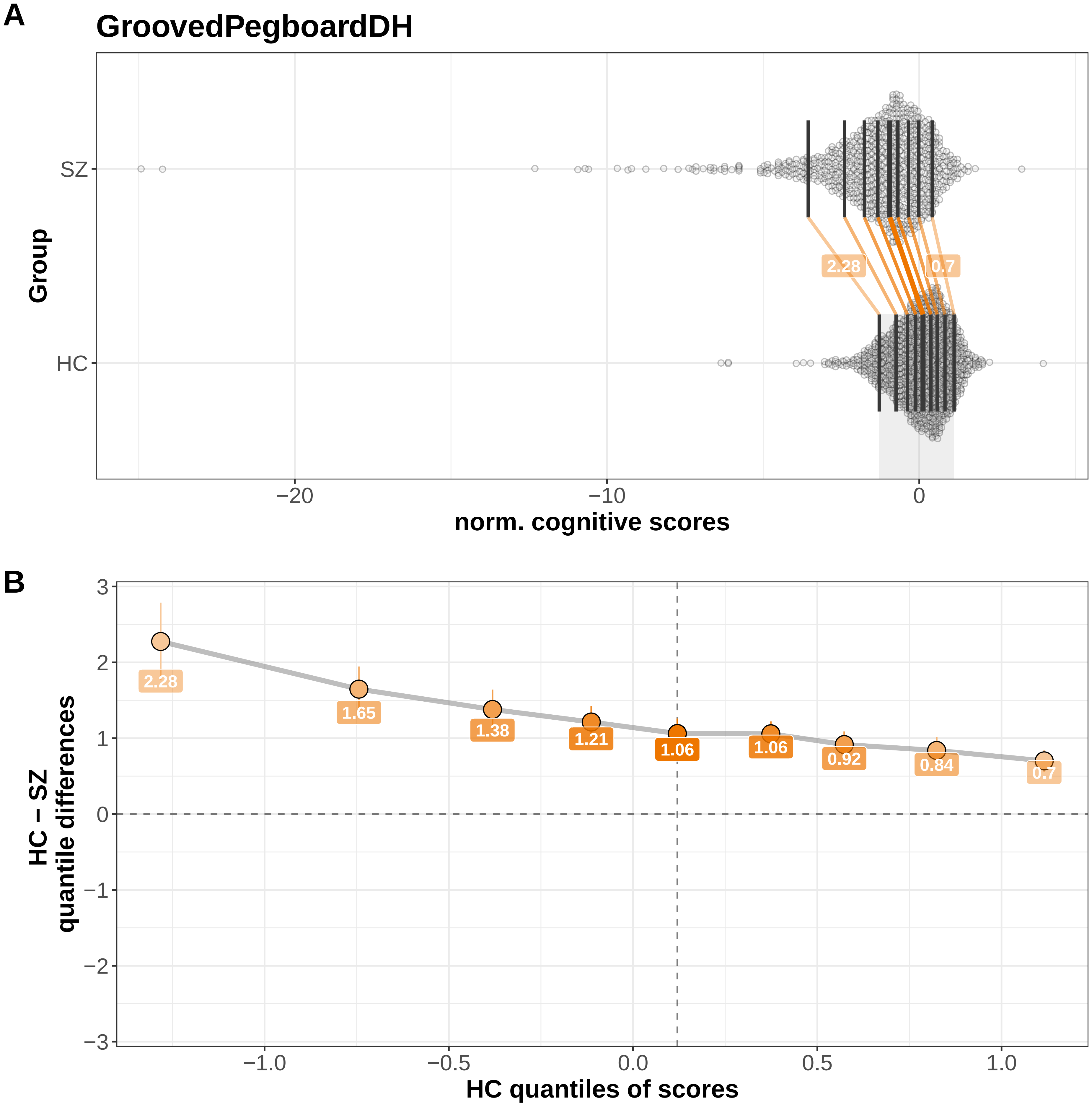

A
GroovedPegboardDH
SZ
HC
Group
2.28
0.7
−20
−10
0
norm. cognitive scores
B
3
2
1
0
−1
−2
−3
HC – SZ
quantile differences
2.28
1.65
1.38
1.21
1.06
1.06
0.92
0.84
0.7
−1.0
−0.5
0.0
0.5
1.0
HC quantiles of scores

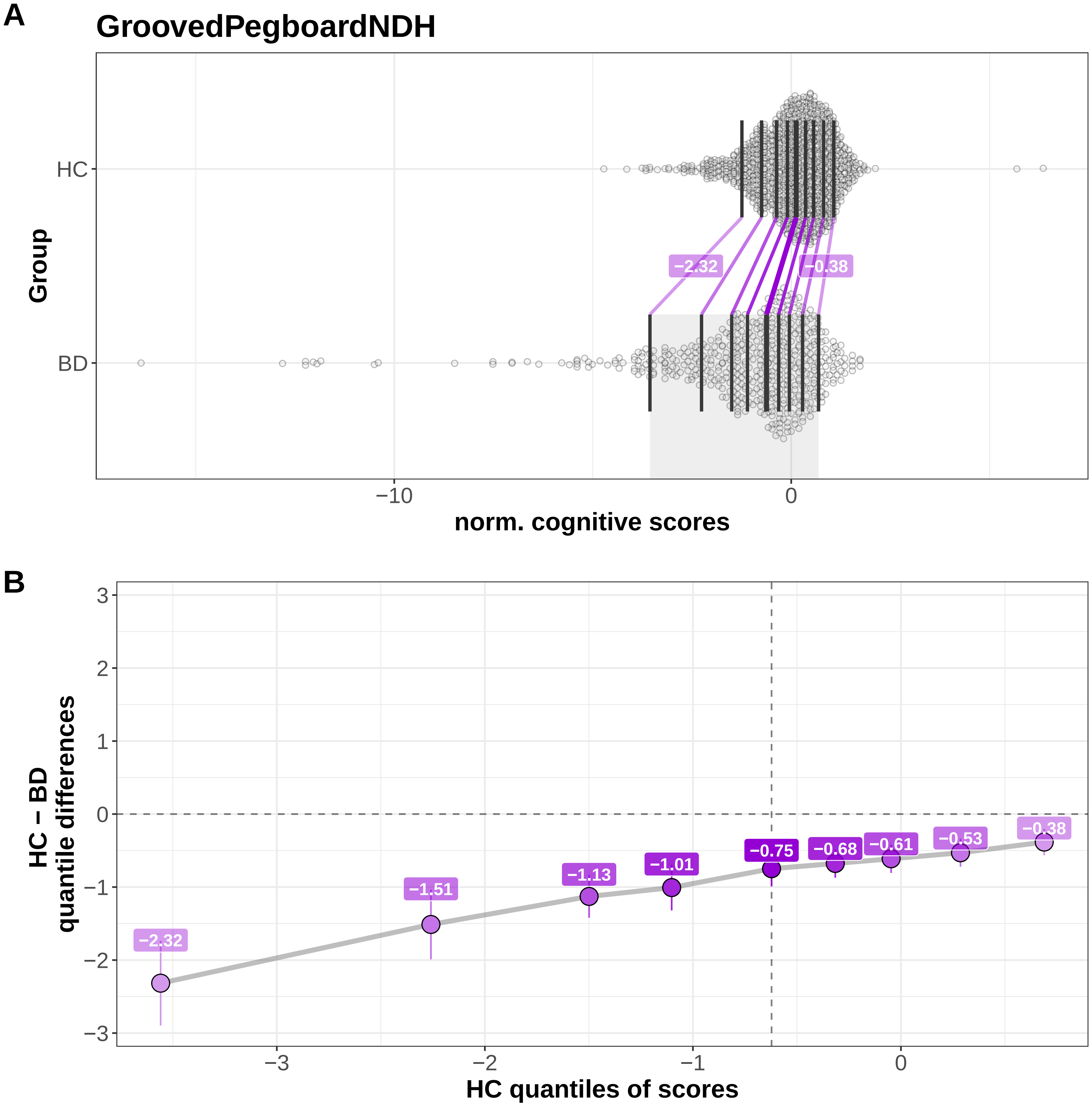

A
GroovedPegboardNDH
HC
BD
Group
−2.32
−0.38
−10
0
norm. cognitive scores
B
HC − BD
quantile differences
3
2
1
0
−1
−2
−3
−2.32
−1.51
−1.13
−1.01
−0.75
−0.68
−0.61
−0.53
−0.38
−3
−2
−1
0
HC quantiles of scores

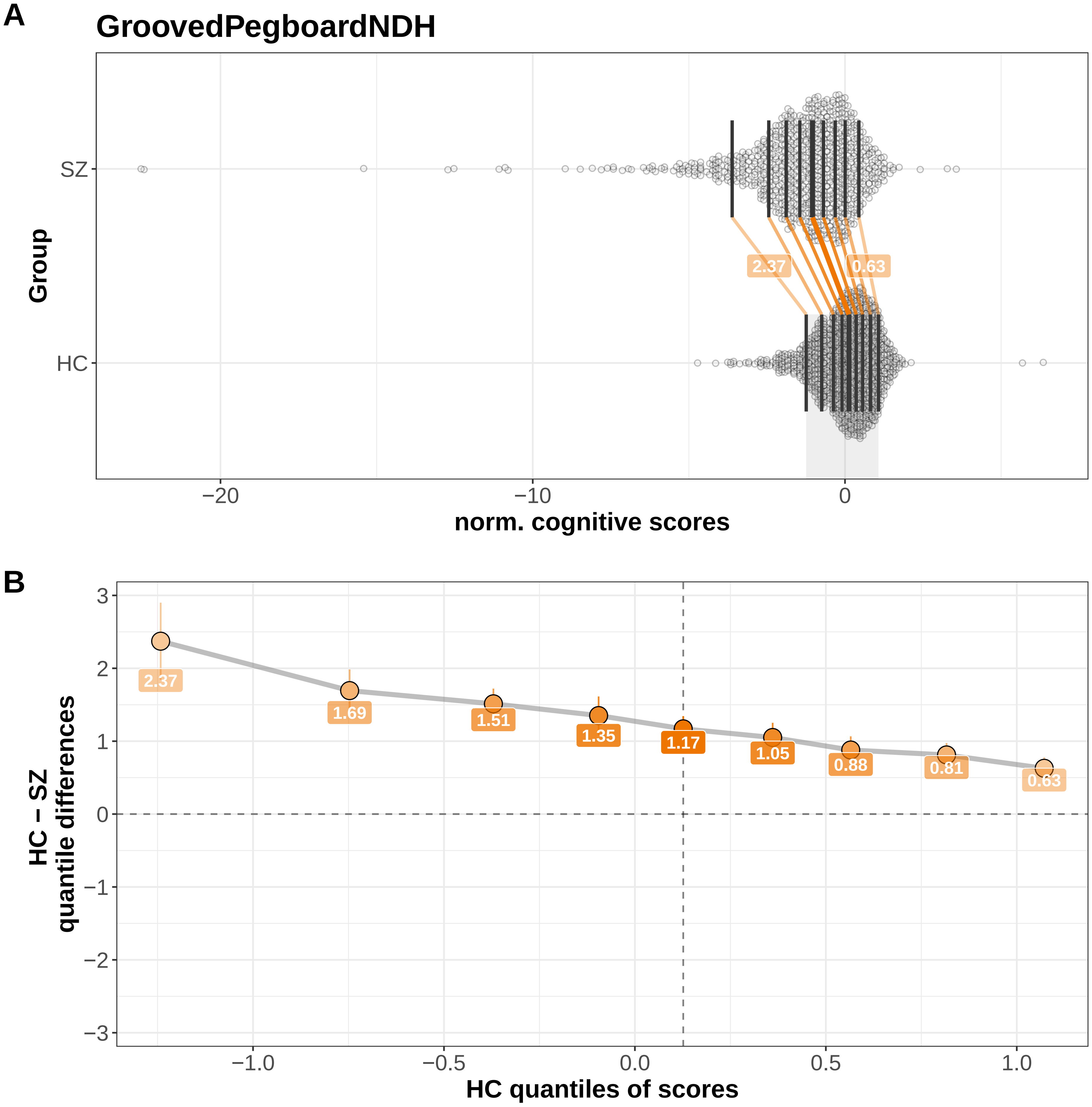

A
GroovedPegboardNDH
Group
SZ
HC
2.37
0.63
−20
−10
0
norm. cognitive scores
B
HC – SZ
quantile differences
3
2
1
0
−1
−2
−3
2.37
1.69
1.51
1.35
1.17
1.05
0.88
0.81
0.63
−1.0
−0.5
0.0
0.5
1.0
HC quantiles of scores

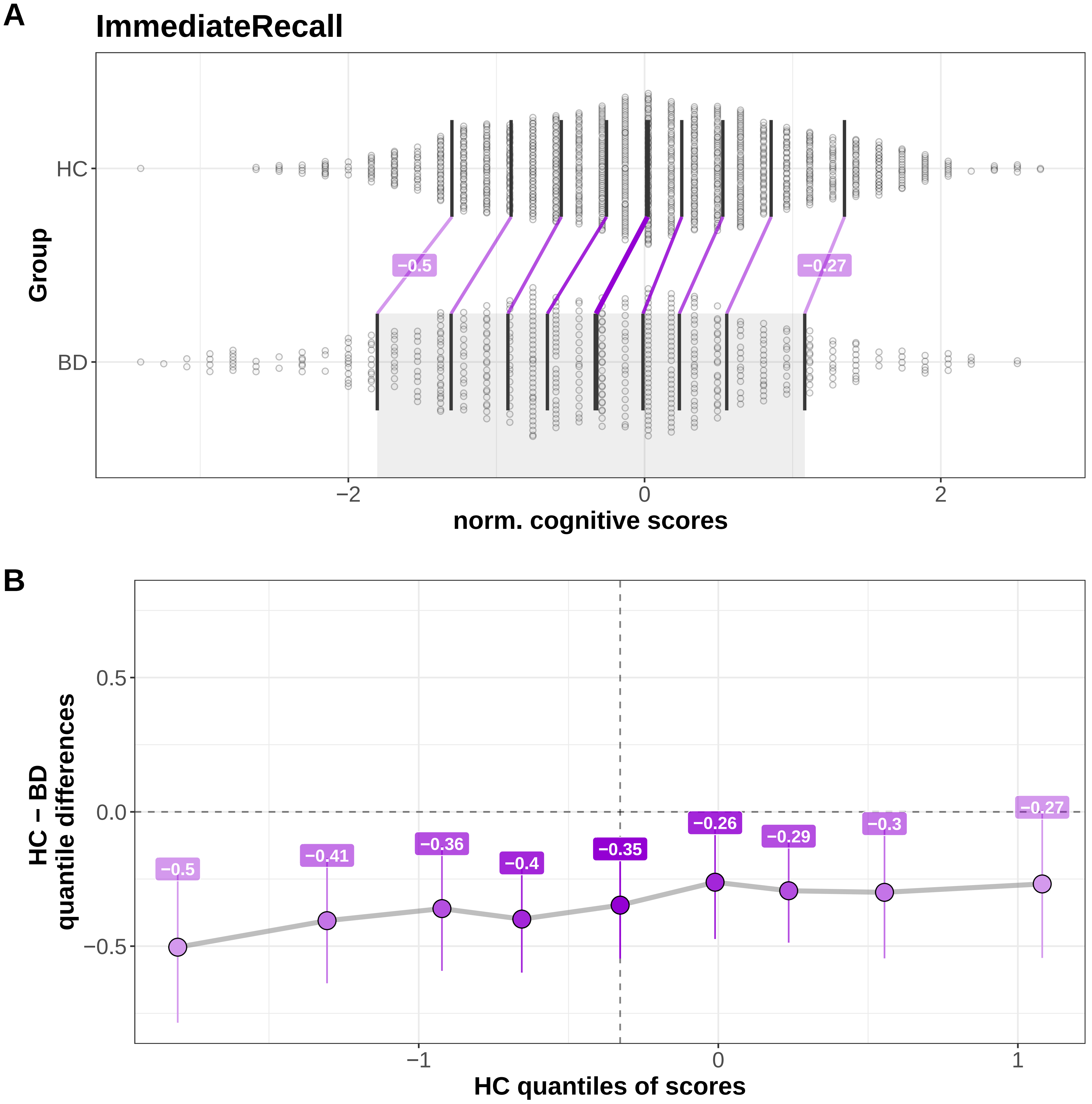

A
ImmediateRecall
Group
HC
BD
−0.5
−0.27
−2
0
2
norm. cognitive scores
B
HC − BD
quantile differences
0.5
0.0
−0.5
−0.5
−0.41
−0.36
−0.4
−0.35
−0.26
−0.29
−0.3
−0.27
−1
0
1
HC quantiles of scores

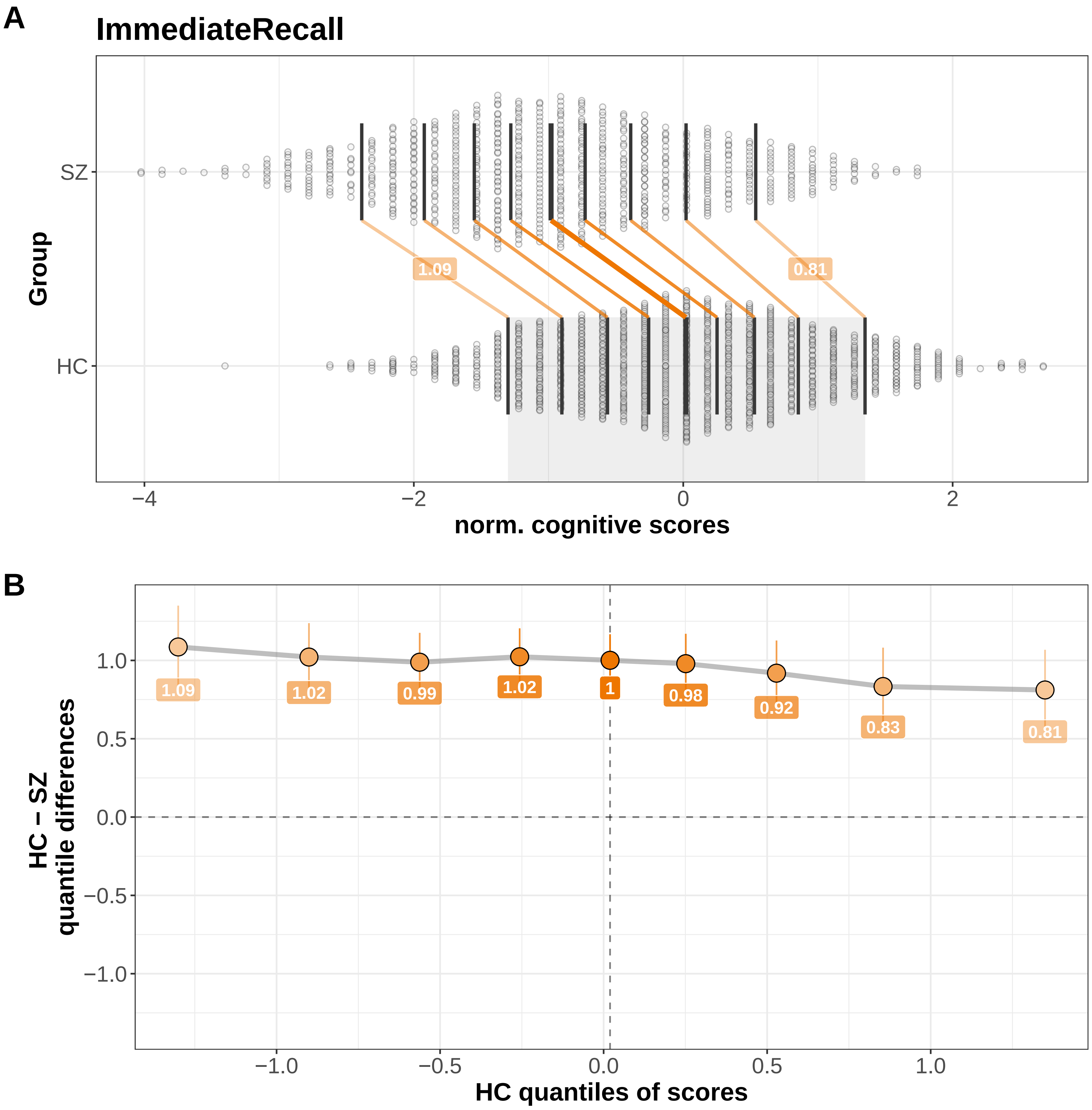

A
ImmediateRecall
Group
SZ
HC
1.09
0.81
−4
−2
0
2
norm. cognitive scores
B
HC − SZ
quantile differences
1.0
0.5
0.0
−0.5
−1.0
1.09
1.02
0.99
1.02
1
0.98
0.92
0.83
0.81
−1.0
−0.5
0.0
0.5
1.0
HC quantiles of scores

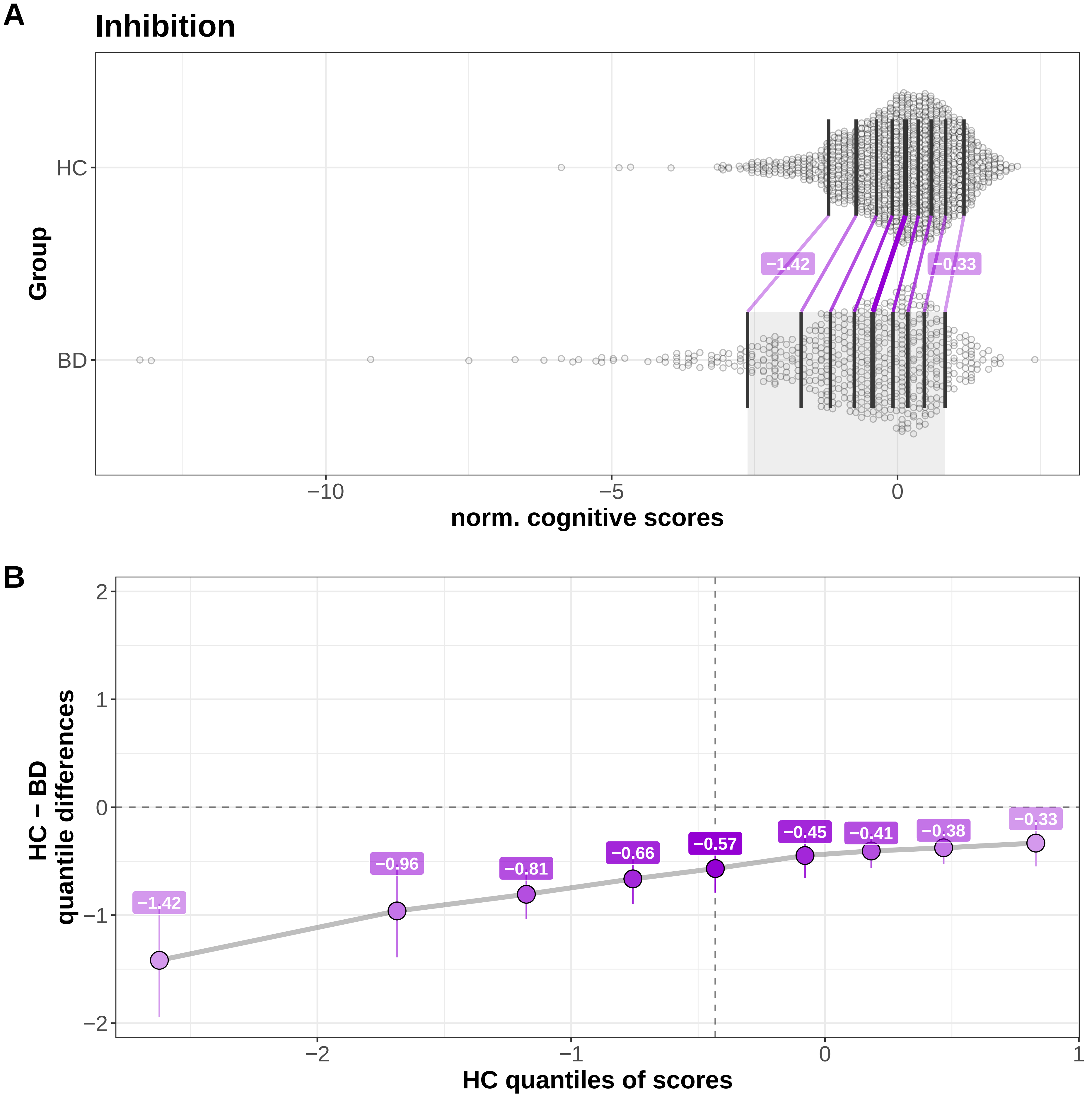

A
Inhibition
Group
HC
BD
−1.42
−0.33
−10
−5
0
norm. cognitive scores
B
HC − BD
quantile differences
2
1
0
−1
−2
−1.42
−0.96
−0.81
−0.66
−0.57
−0.45
−0.41
−0.38
−0.33
HC quantiles of scores

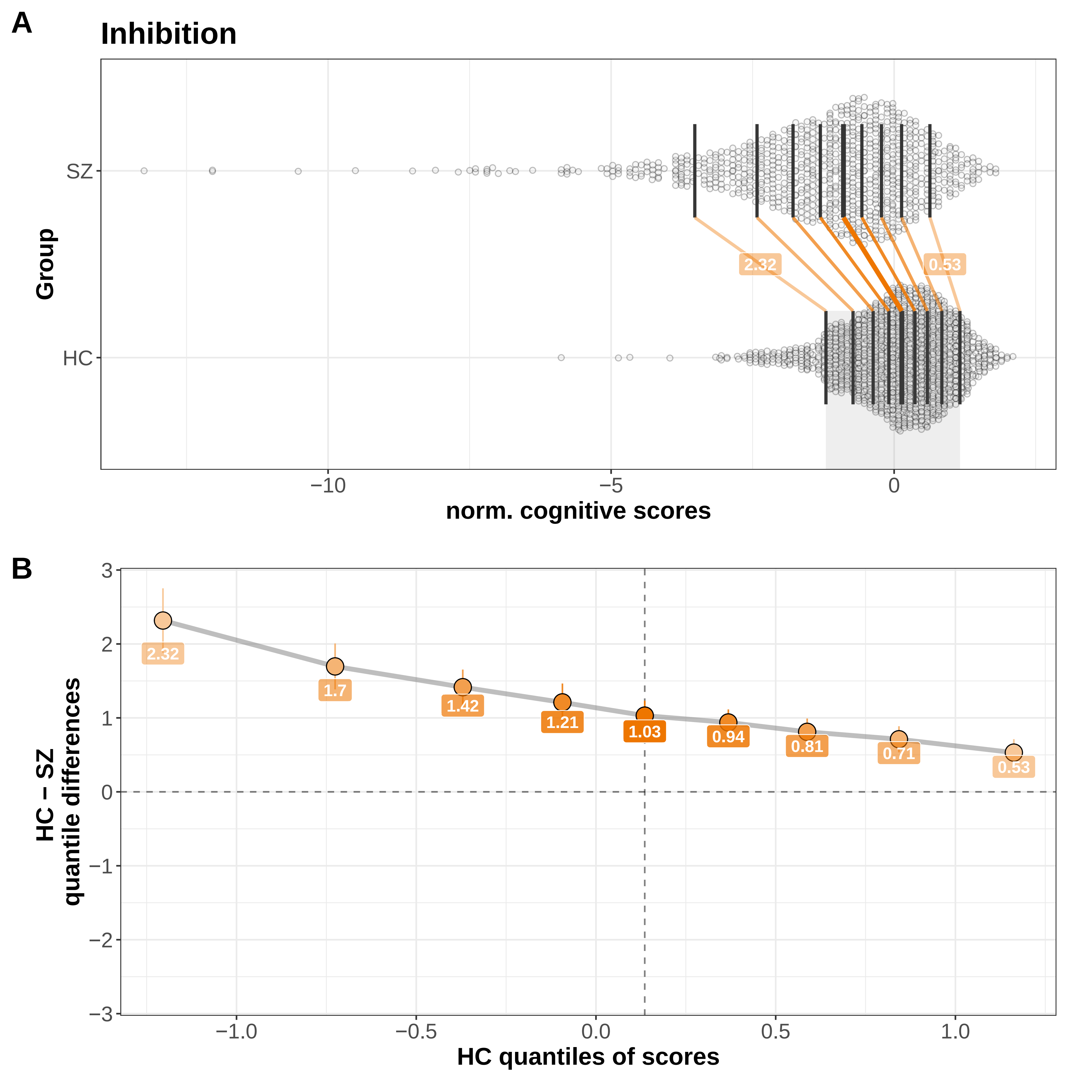
A
Inhibition
Group
SZ
HC
2.32
0.53
−10
−5
0
norm. cognitive scores
B
HC − SZ
quantile differences
3
2
1
0
−1
−2
−3
2.32
1.7
1.42
1.21
1.03
0.94
0.81
0.71
0.53
−1.0
−0.5
0.0
0.5
1.0
HC quantiles of scores

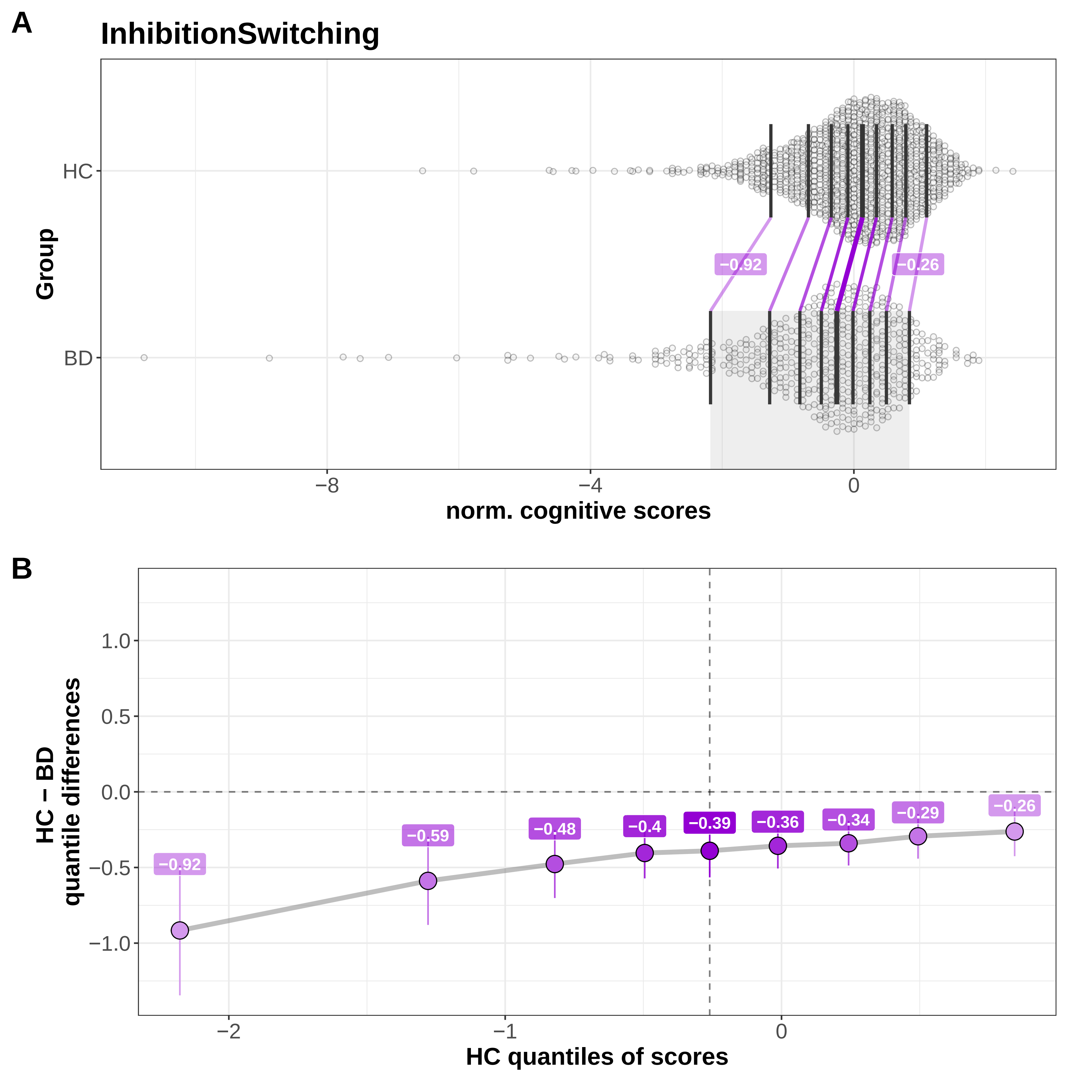
A
InhibitionSwitching
Group
HC
BD
−0.92
−0.26
−8
−4
0
norm. cognitive scores
B
HC – BD
quantile differences
1.0
0.5
0.0
−0.5
−1.0
−0.92
−0.59
−0.48
−0.4
−0.39
−0.36
−0.34
−0.29
−0.26
−2
−1
0
HC quantiles of scores

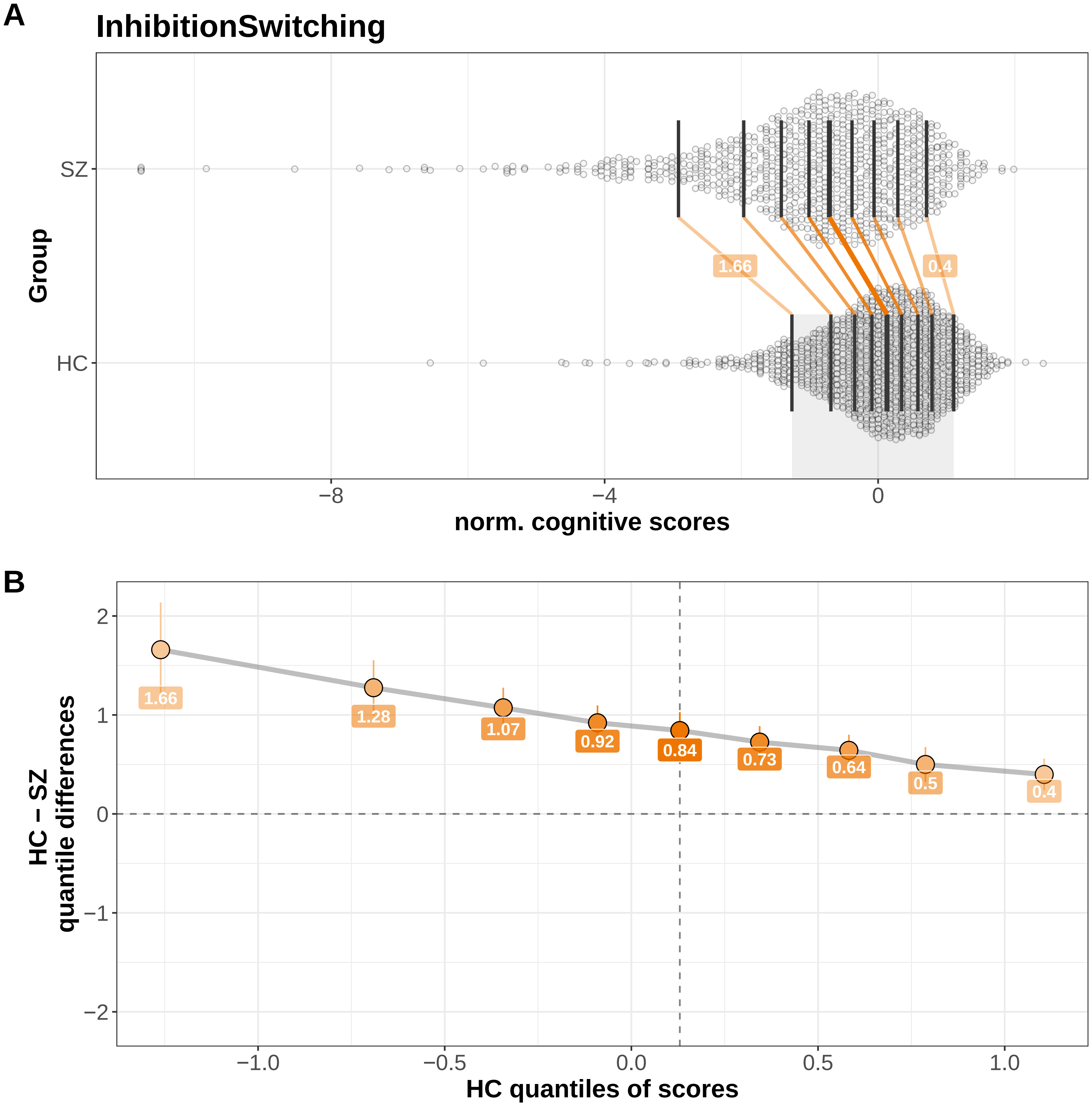
A
InhibitionSwitching
Group
SZ
HC
1.66
0.4
−8
−4
0
norm. cognitive scores
B
HC – SZ
quantile differences
2
1
0
−1
−2
1.66
1.28
1.07
0.92
0.84
0.73
0.64
0.5
0.4
−1.0
−0.5
0.0
0.5
1.0
HC quantiles of scores

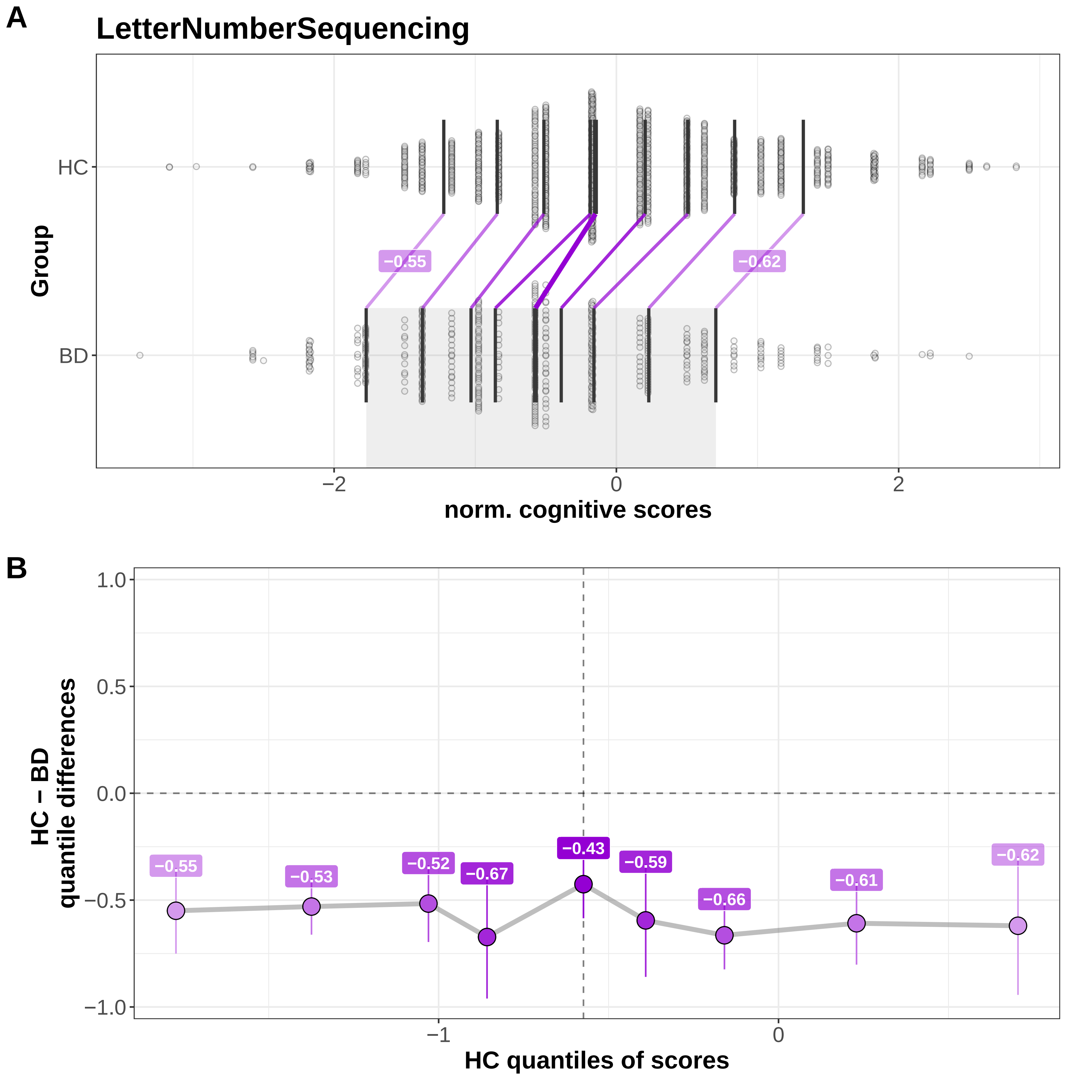

A
LetterNumberSequencing
Group
HC
BD
−0.55
−0.62
−2
0
2
norm. cognitive scores
B
HC − BD
quantile differences
1.0
0.5
0.0
−0.5
−1.0
−0.55
−0.53
−0.52
−0.67
−0.43
−0.59
−0.66
−0.61
−0.62
−1
0
HC quantiles of scores

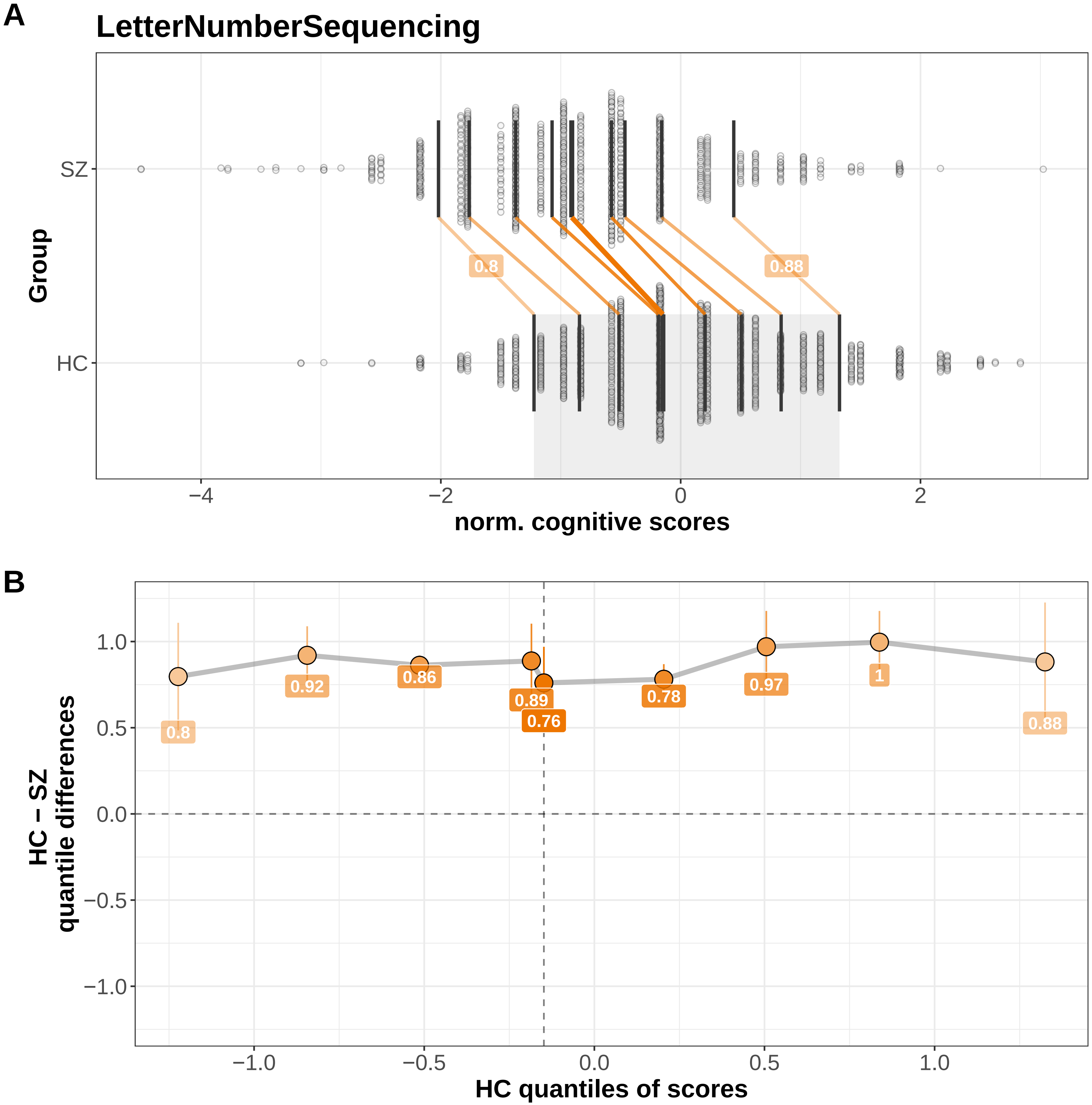
A
LetterNumberSequencing
Group
SZ
HC
0.8
0.88
−4
−2
0
2
norm. cognitive scores
B
HC − SZ
quantile differences
1.0
0.5
0.0
−0.5
−1.0
0.8
0.92
0.86
0.89
0.76
0.78
0.97
1
0.88
−1.0
−0.5
0.0
0.5
1.0
HC quantiles of scores

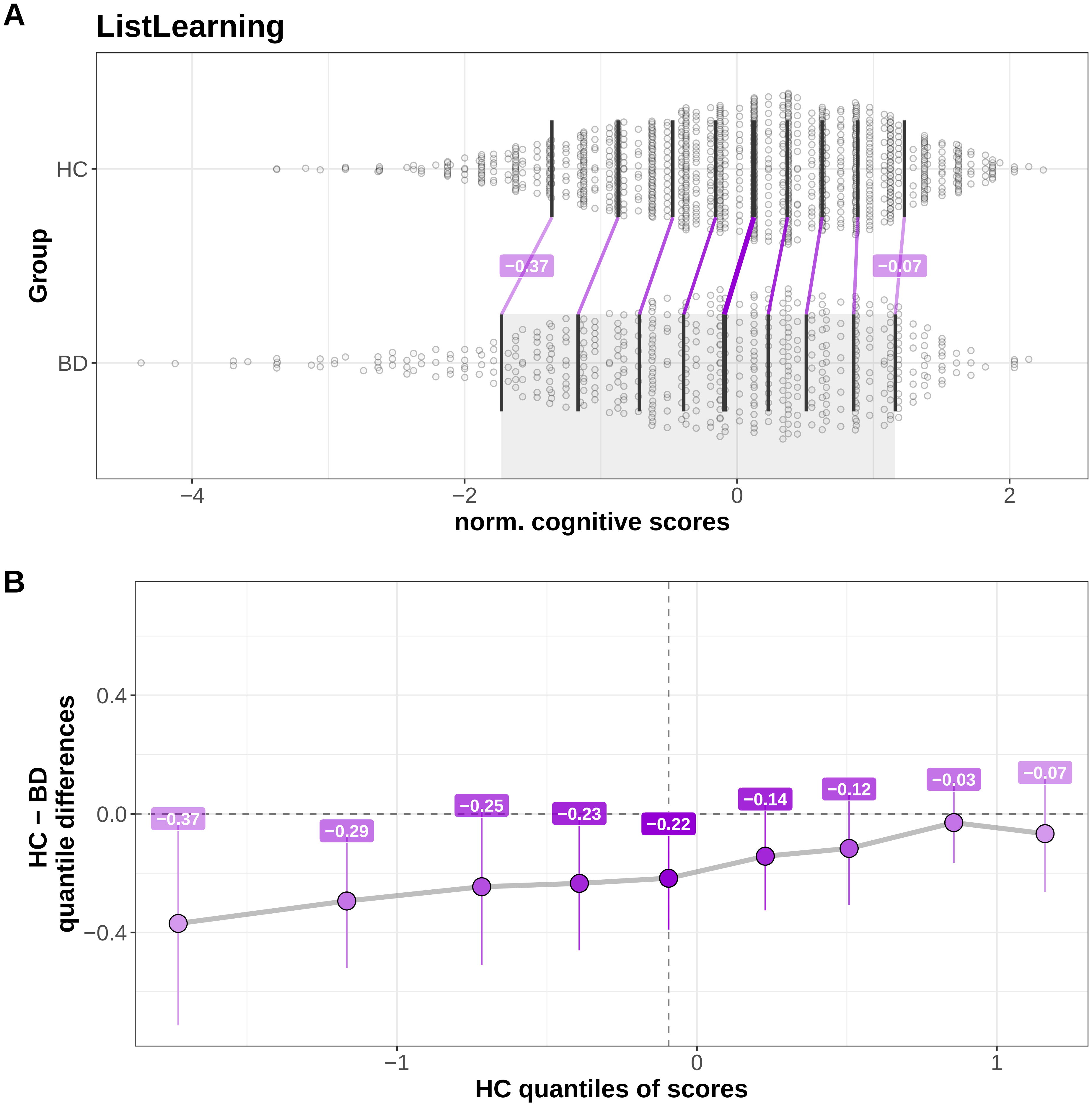
A
ListLearning
Group
HC
BD
−0.37
−0.07
−4
−2
0
2
norm. cognitive scores
B
HC − BD
quantile differences
0.4
0.0
−0.4
−0.37
−0.29
−0.25
−0.23
−0.22
−0.14
−0.12
−0.03
−0.07
−1
0
1
HC quantiles of scores

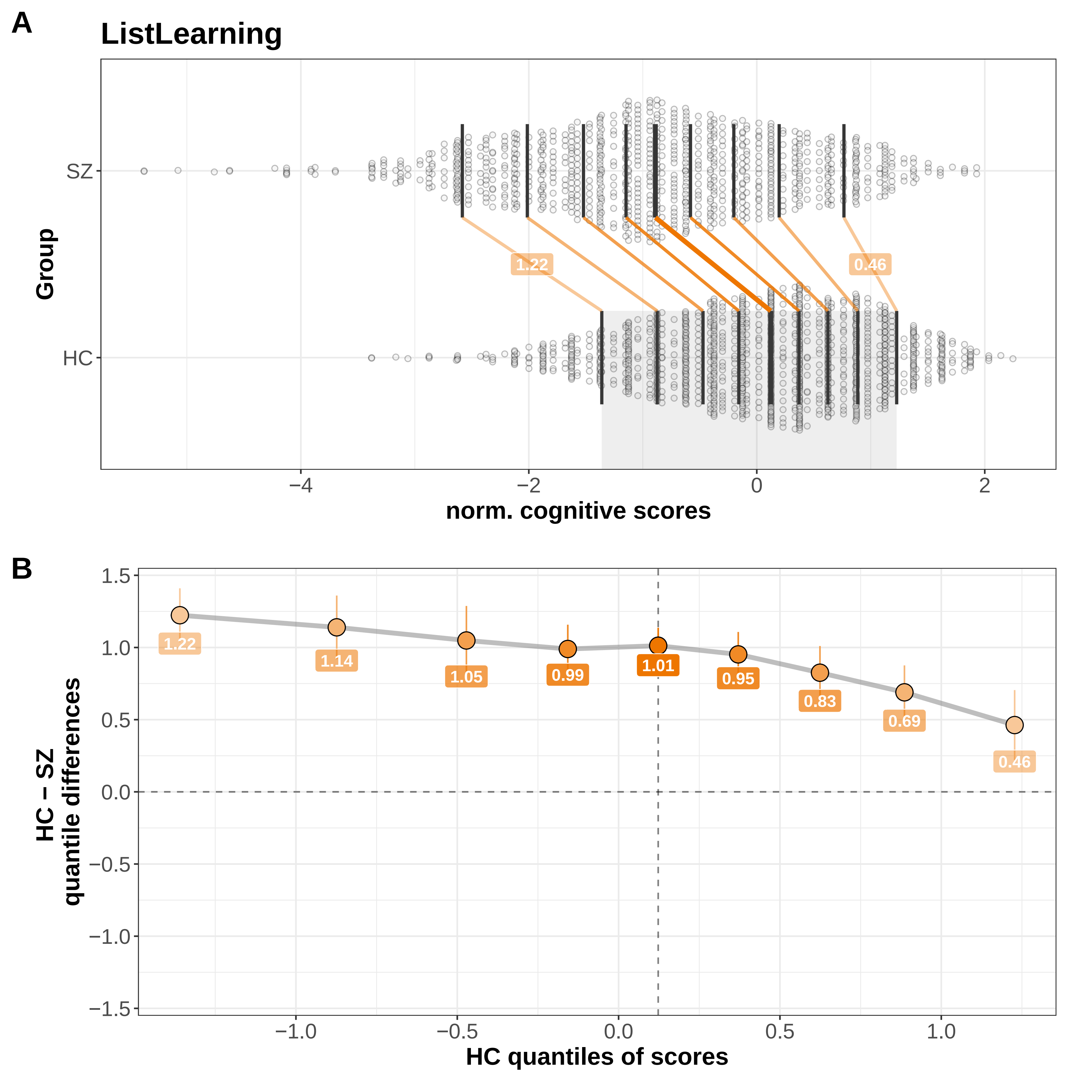

A
ListLearning
Group
SZ
HC
1.22
0.46
−4
−2
0
2
norm. cognitive scores
B
HC – SZ
quantile differences
1.5
1.0
0.5
0.0
−0.5
−1.0
−1.5
1.22
1.14
1.05
0.99
1.01
0.95
0.83
0.69
0.46
−1.0
−0.5
0.0
0.5
1.0
HC quantiles of scores

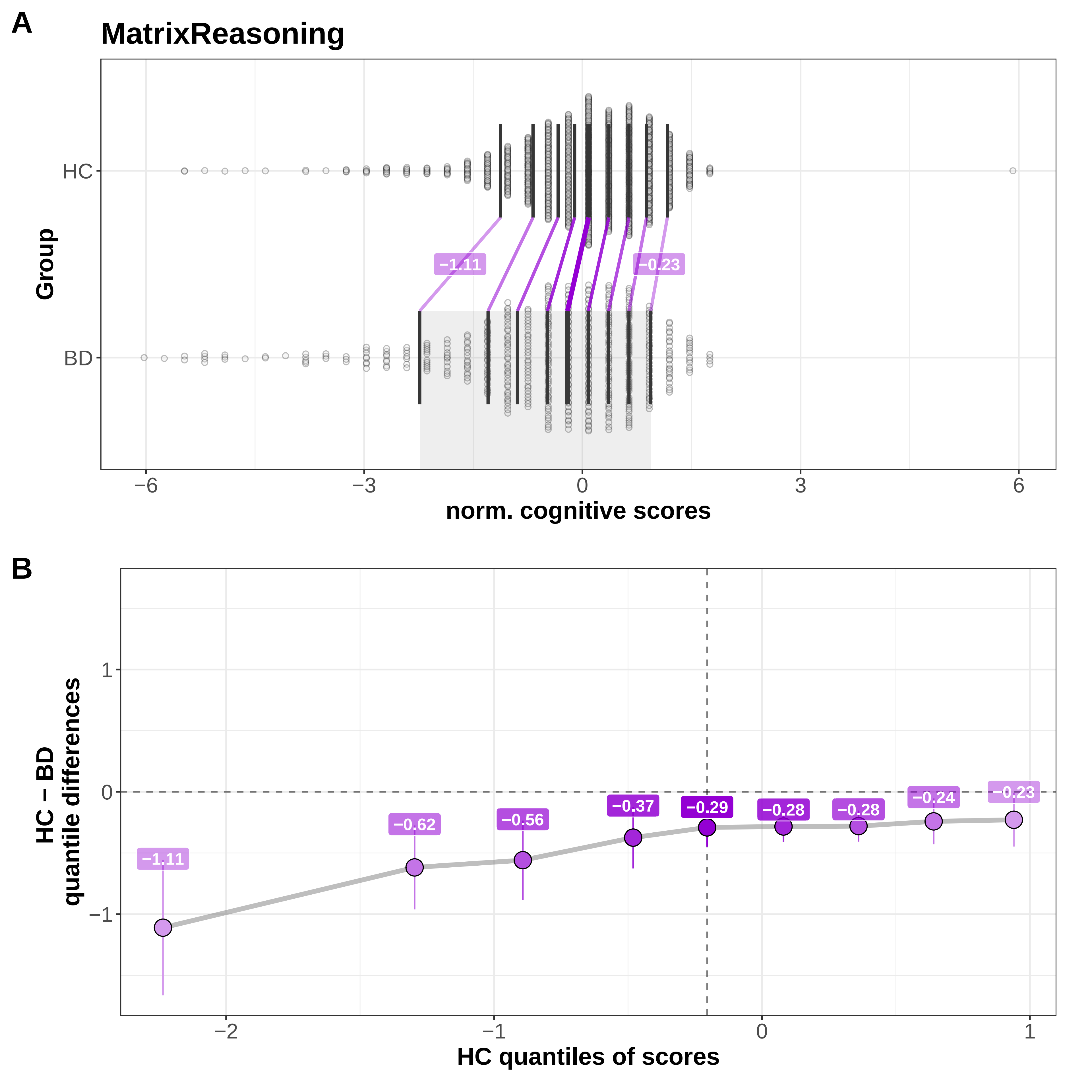
A
MatrixReasoning
HC
BD
Group
−1.11
−0.23
−6
−3
0
3
6
norm. cognitive scores
B
HC − BD
quantile differences
1
0
−1
−1.11
−0.62
−0.56
−0.37
−0.29
−0.28
−0.28
−0.24
−0.23
−2
−1
0
1
HC quantiles of scores

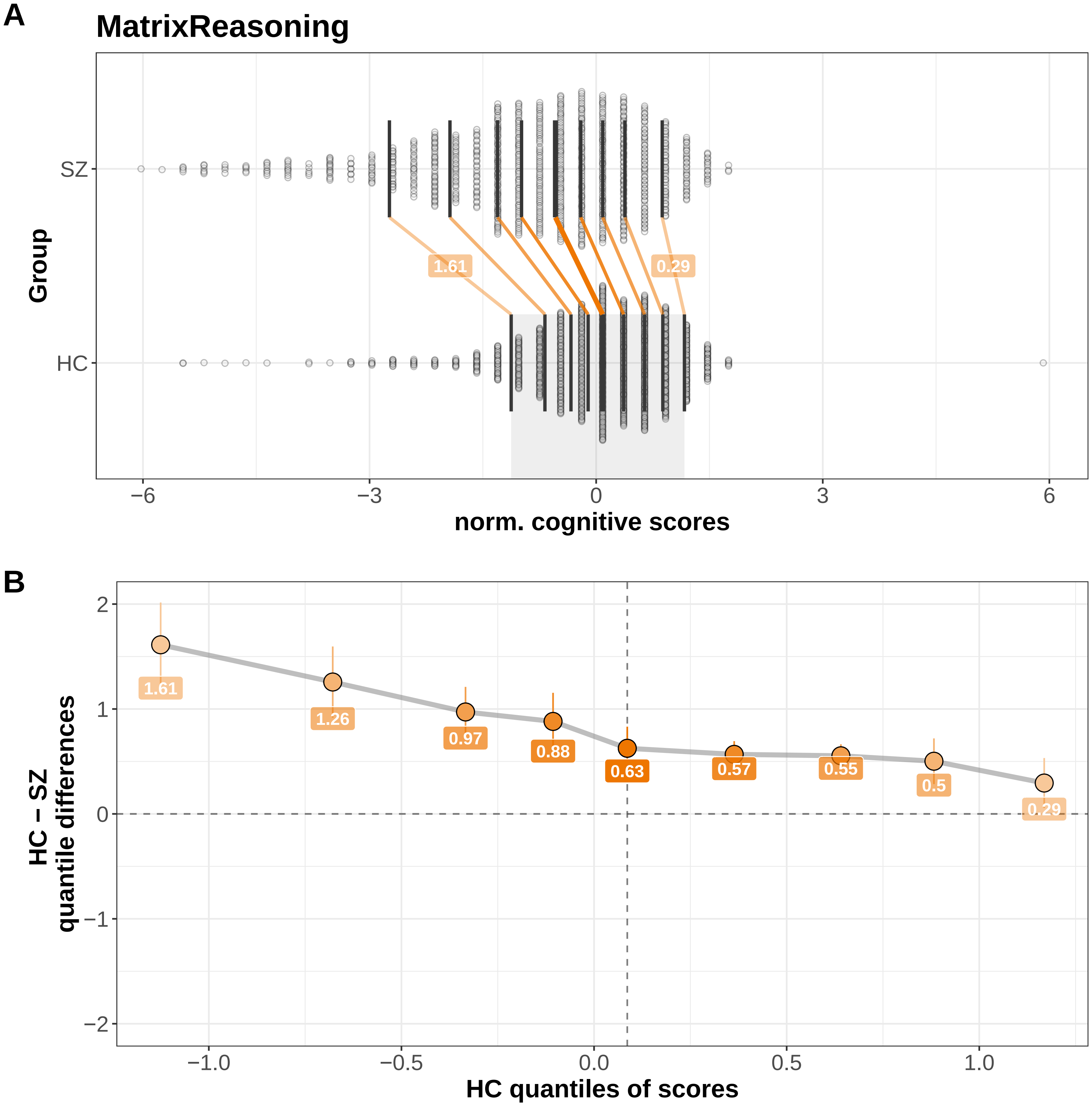

A
MatrixReasoning
SZ
HC
Group
1.61
0.29
−6
−3
0
3
6
norm. cognitive scores
B
1.61
1.26
0.97
0.88
0.63
0.57
0.55
0.5
0.29
2
1
0
−1
−2
HC − SZ
quantile differences
−1.0
−0.5
0.0
0.5
1.0
HC quantiles of scores

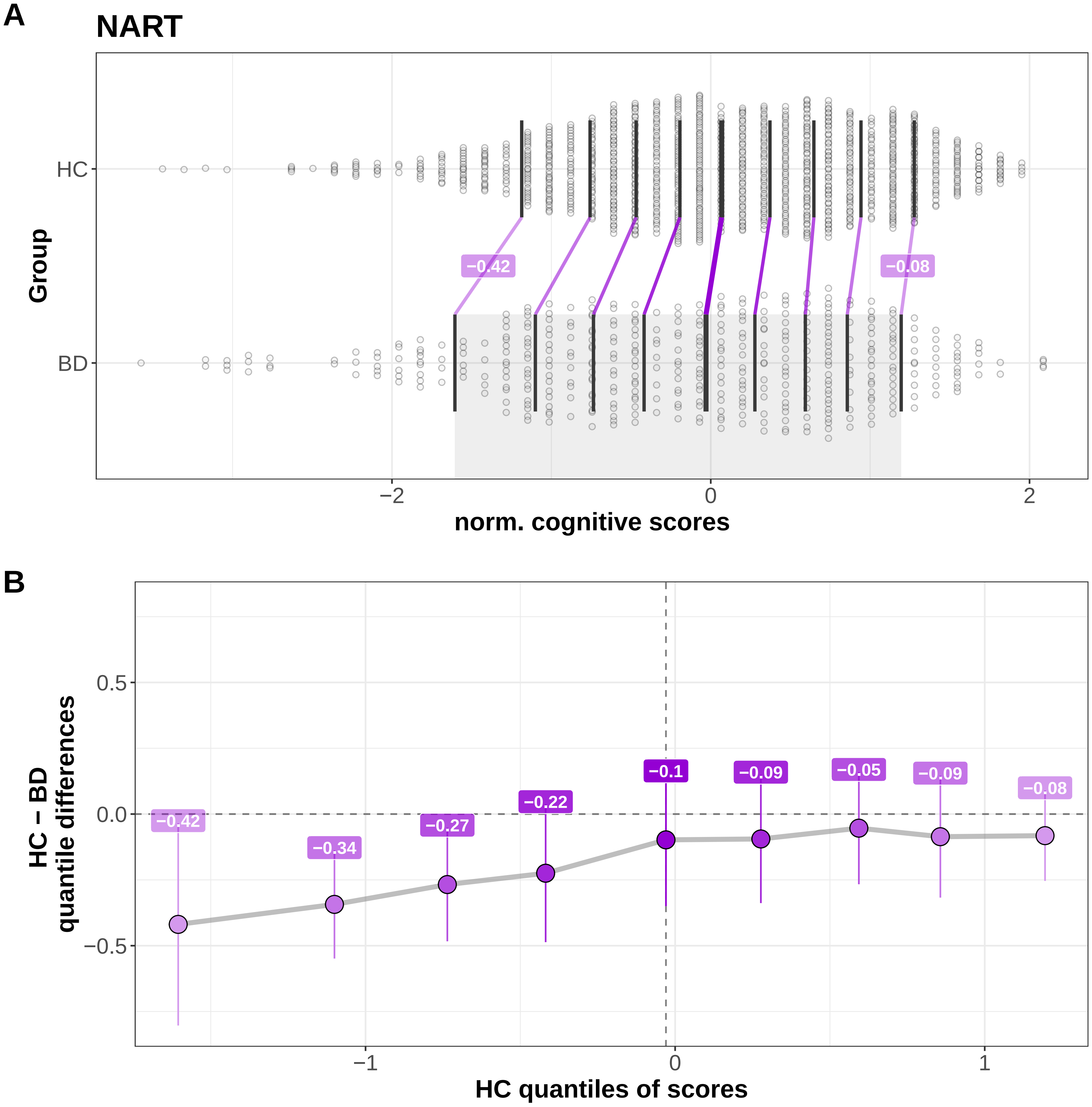
A
NART
HC
BD
Group
−0.42
−0.08
−2
0
2
norm. cognitive scores
B
0.5
0.0
−0.5
HC – BD
quantile differences
−0.42
−0.34
−0.27
−0.22
−0.1
−0.09
−0.05
−0.09
−0.08
−1
0
1
HC quantiles of scores

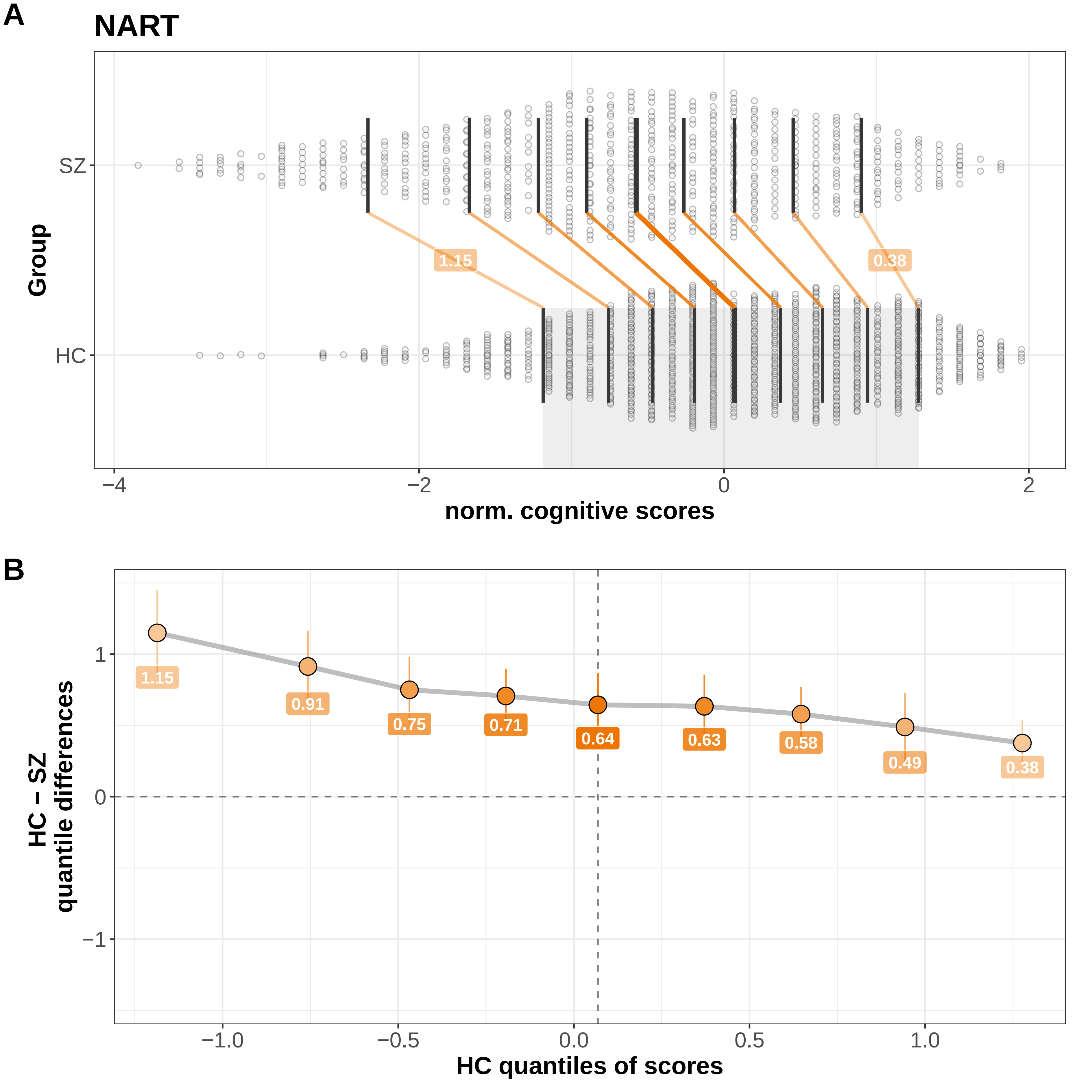
A
NART
SZ
HC
Group
1.15
0.38
−4
−2
0
2
norm. cognitive scores
B
1.15
0.91
0.75
0.71
0.64
0.63
0.58
0.49
0.38
HC − SZ
quantile differences
1
0
−1
−1.0
−0.5
0.0
0.5
1.0
HC quantiles of scores

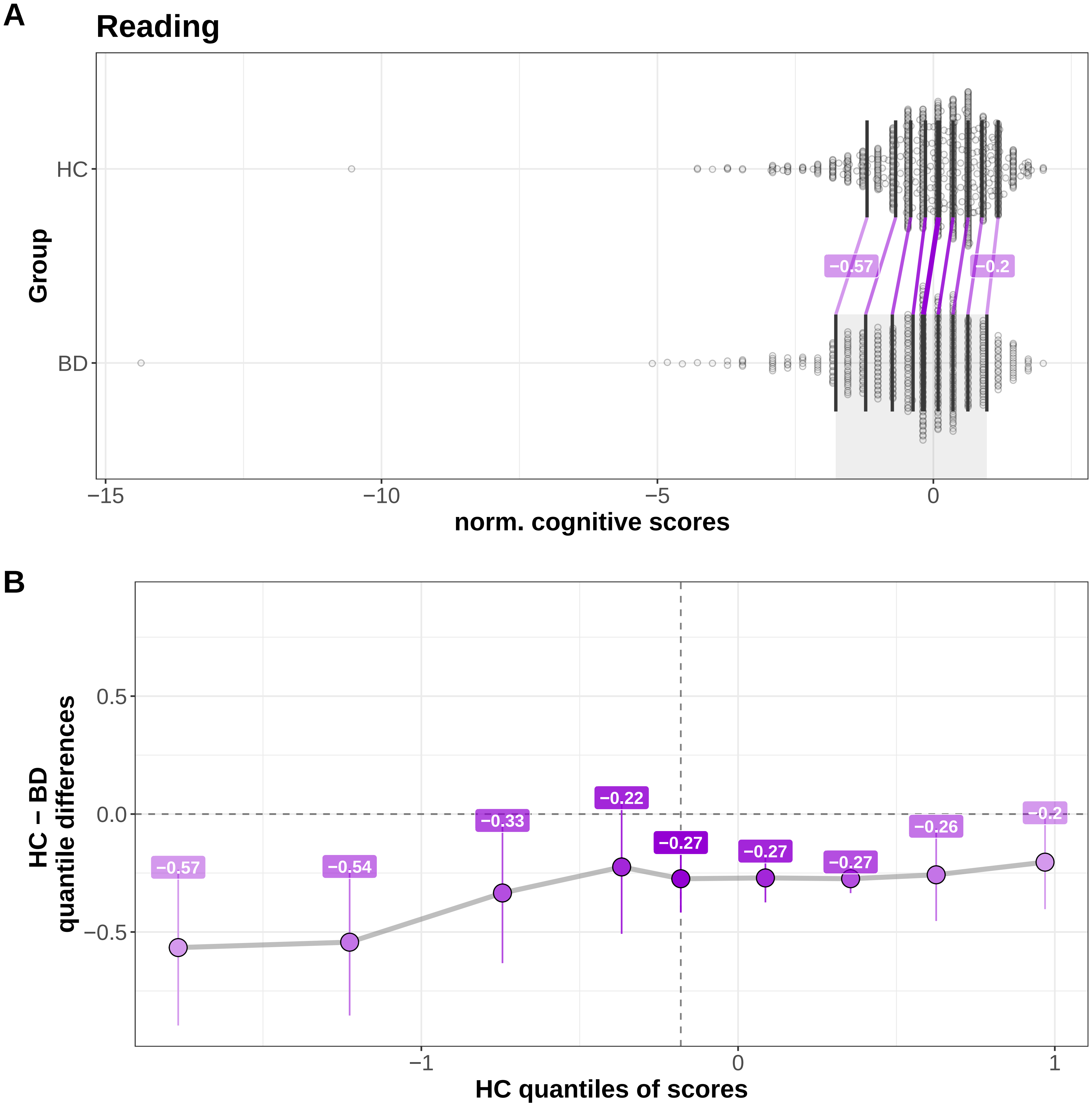

A
Reading
HC
BD
Group
−0.57
−0.2
−15
−10
−5
0
norm. cognitive scores
B
HC − BD
quantile differences
0.5
0.0
−0.5
−0.57
−0.54
−0.33
−0.22
−0.27
−0.27
−0.27
−0.26
−0.2
−1
0
1
HC quantiles of scores

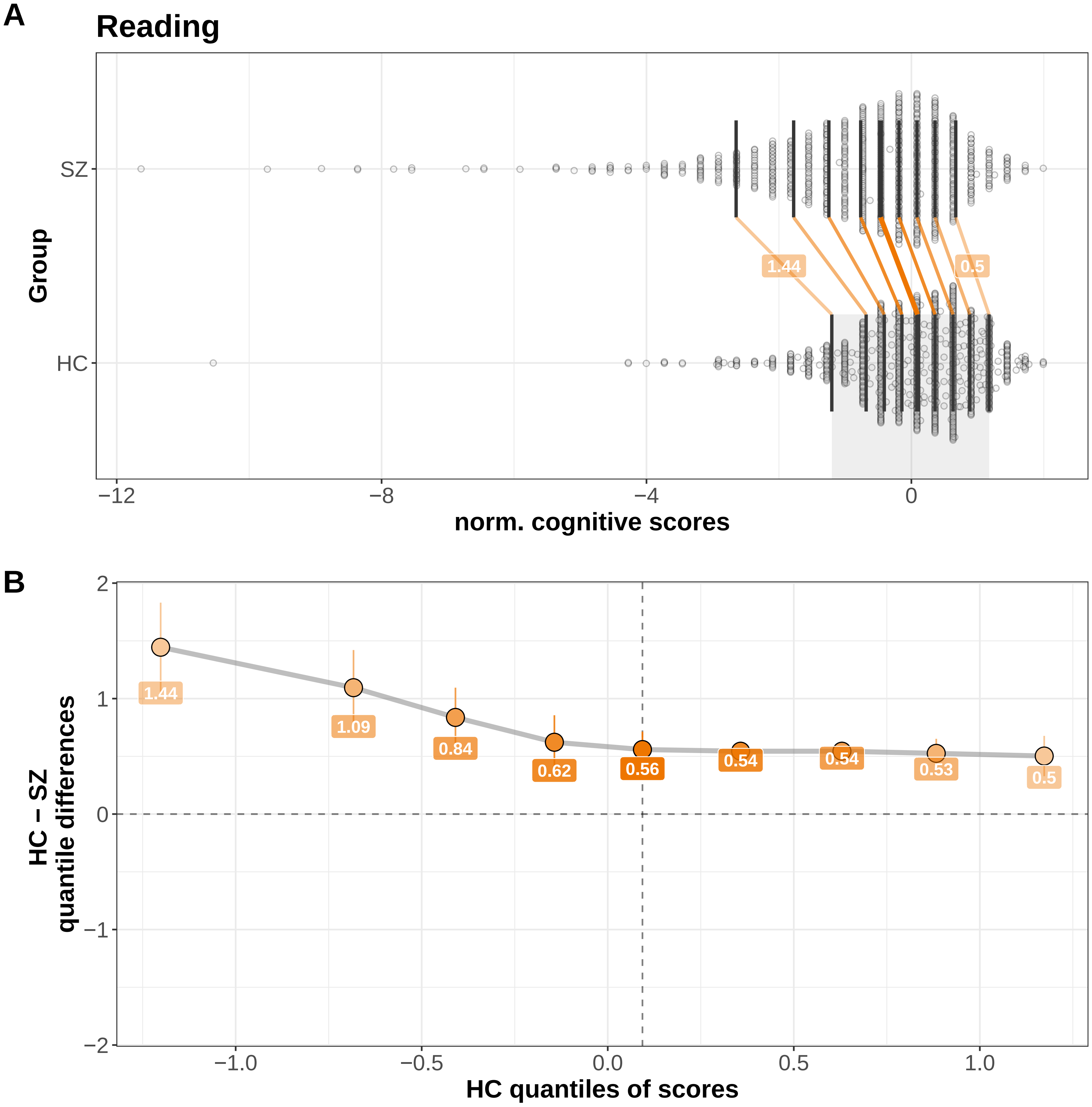
A
Reading
SZ
HC
Group
1.44
0.5
−12
−8
−4
0
norm. cognitive scores
B
2
1
0
−1
−2
1.44
1.09
0.84
0.62
0.56
0.54
0.54
0.53
0.5
HC − SZ
quantile differences
−1.0
−0.5
0.0
0.5
1.0
HC quantiles of scores

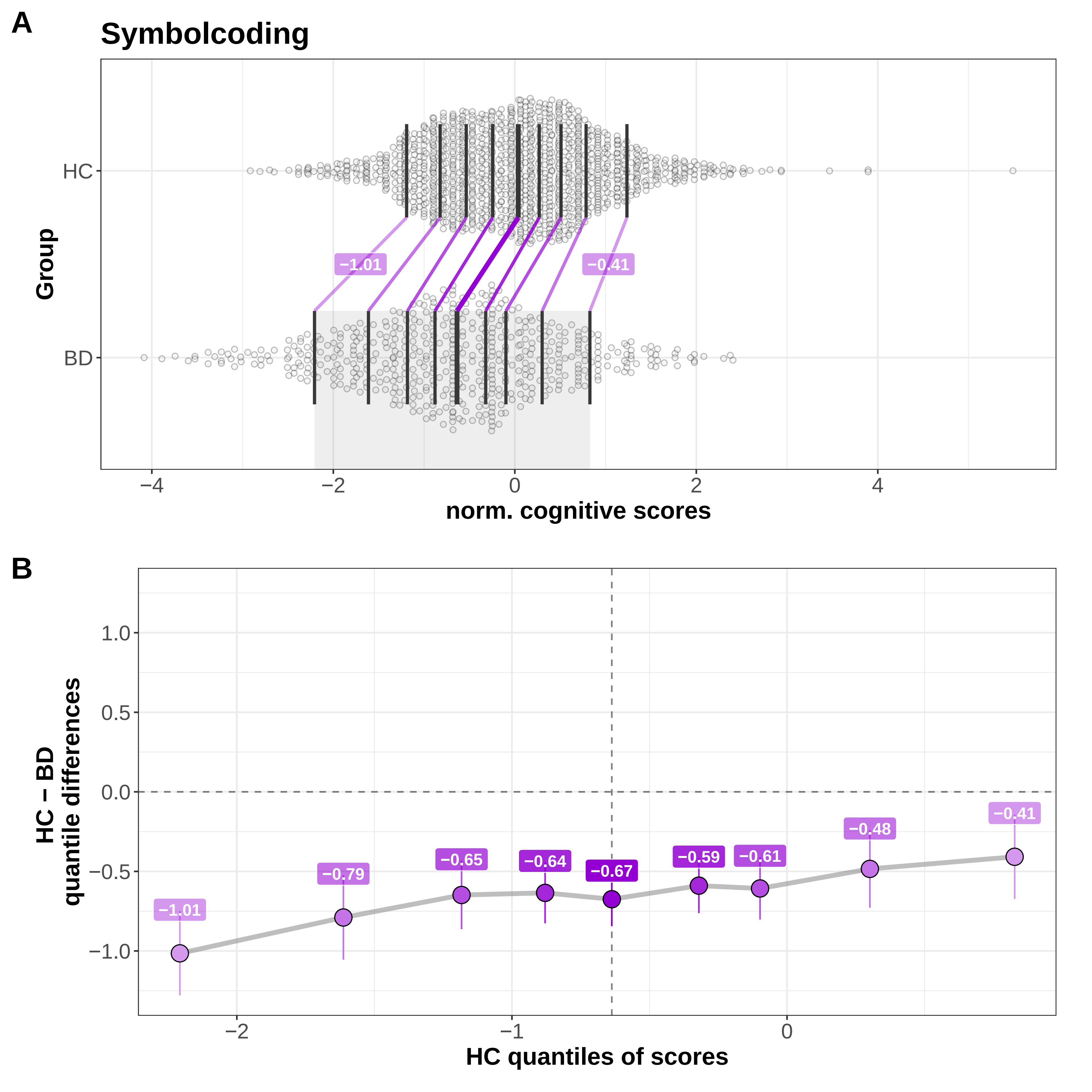

A
Symbolcoding
HC
BD
Group
−1.01
−0.41
−4
−2
0
2
4
norm. cognitive scores
B
1.0
0.5
0.0
−0.5
−1.0
HC − BD
quantile differences
−1.01
−0.79
−0.65
−0.64
−0.67
−0.59
−0.61
−0.48
−0.41
−2
−1
0
HC quantiles of scores

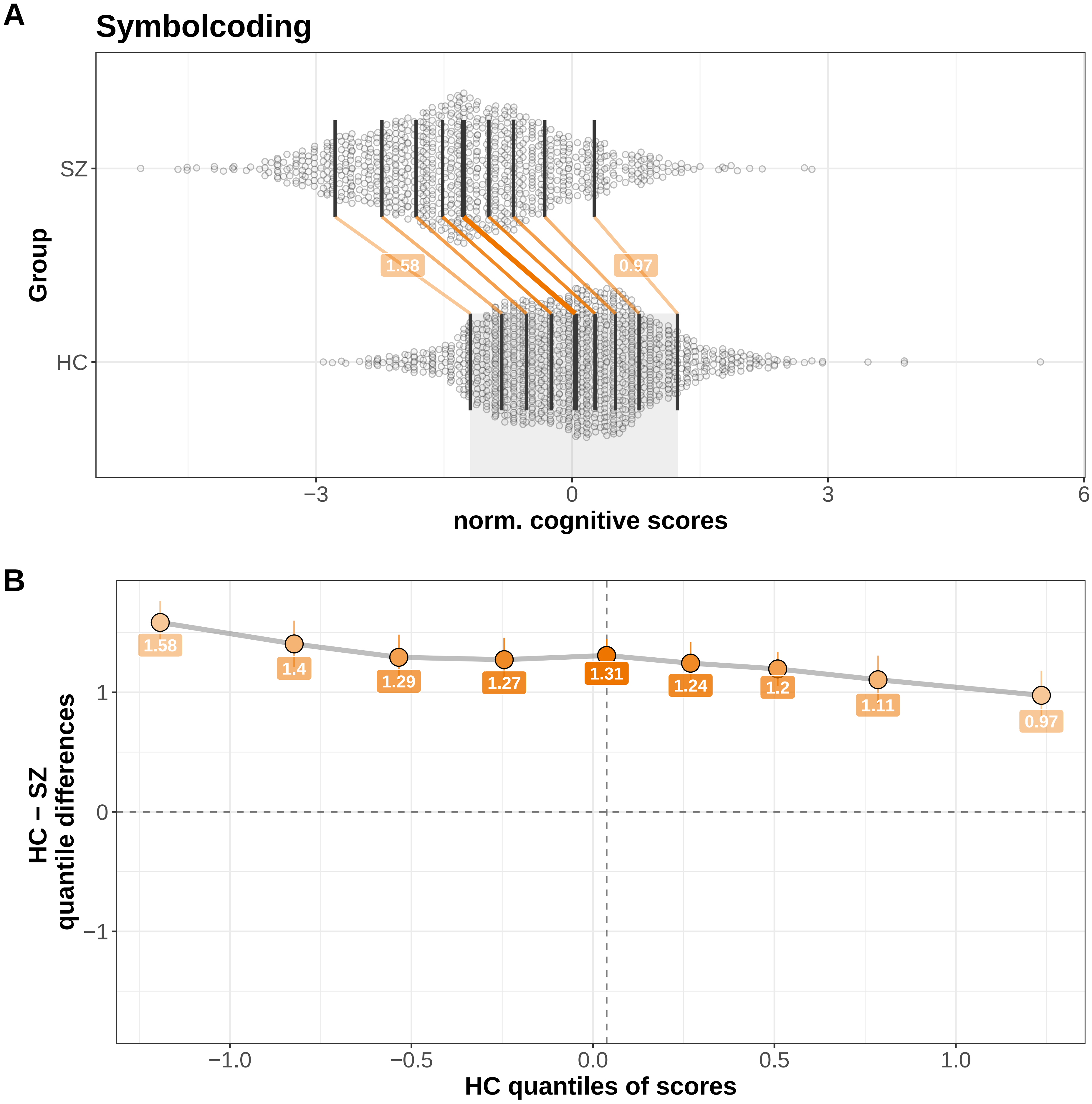
A
Symbolcoding
Group
SZ
HC
1.58
0.97
−3
0
3
6
norm. cognitive scores
B
1.58
1.4
1.29
1.27
1.31
1.24
1.2
1.11
0.97
HC − SZ
quantile differences
1
0
−1
−1.0
−0.5
0.0
0.5
1.0
HC quantiles of scores

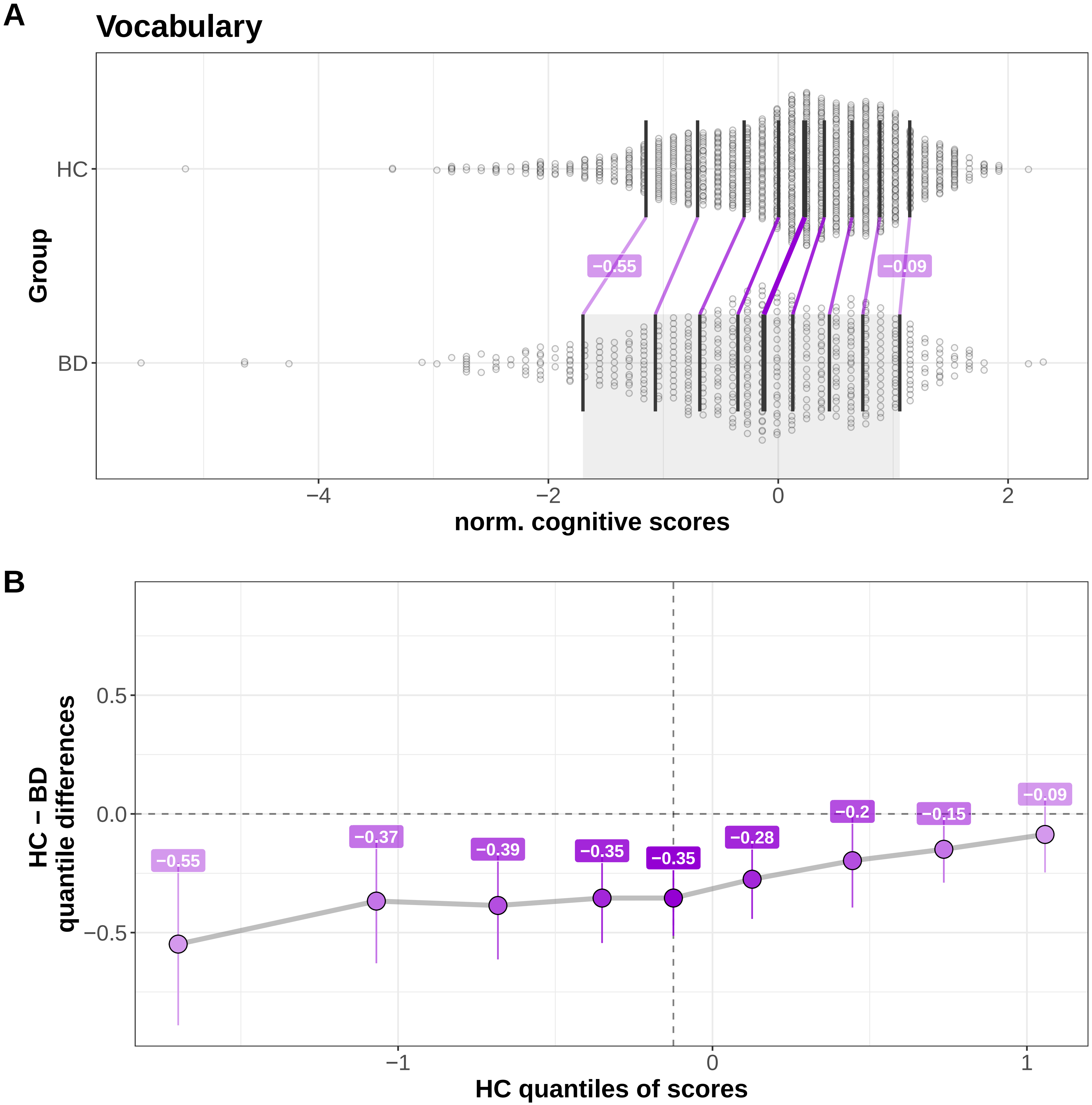

A
Vocabulary
Group
HC
BD
−0.55
−0.09
−4
−2
0
2
norm. cognitive scores
B
HC – BD
quantile differences
0.5
0.0
−0.5
−0.55
−0.37
−0.39
−0.35
−0.35
−0.28
−0.2
−0.15
−0.09
−1
0
1
HC quantiles of scores

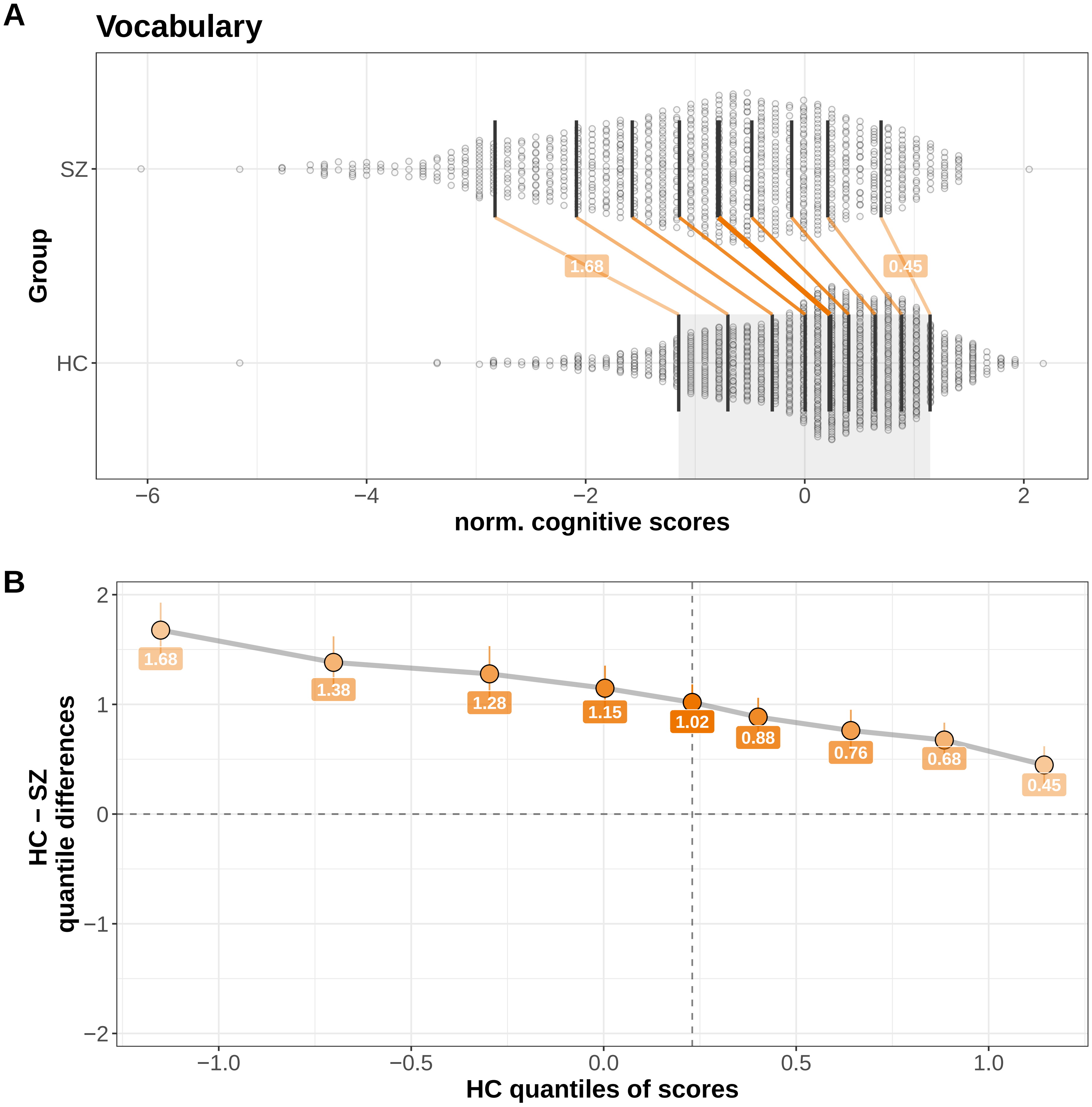
A
Vocabulary
Group
SZ
HC
1.68
0.45
−6
−4
−2
0
2
norm. cognitive scores
B
HC − SZ
quantile differences
2
1
0
−1
−2
1.68
1.38
1.28
1.15
1.02
0.88
0.76
0.68
0.45
−1.0
−0.5
0.0
0.5
1.0
HC quantiles of scores

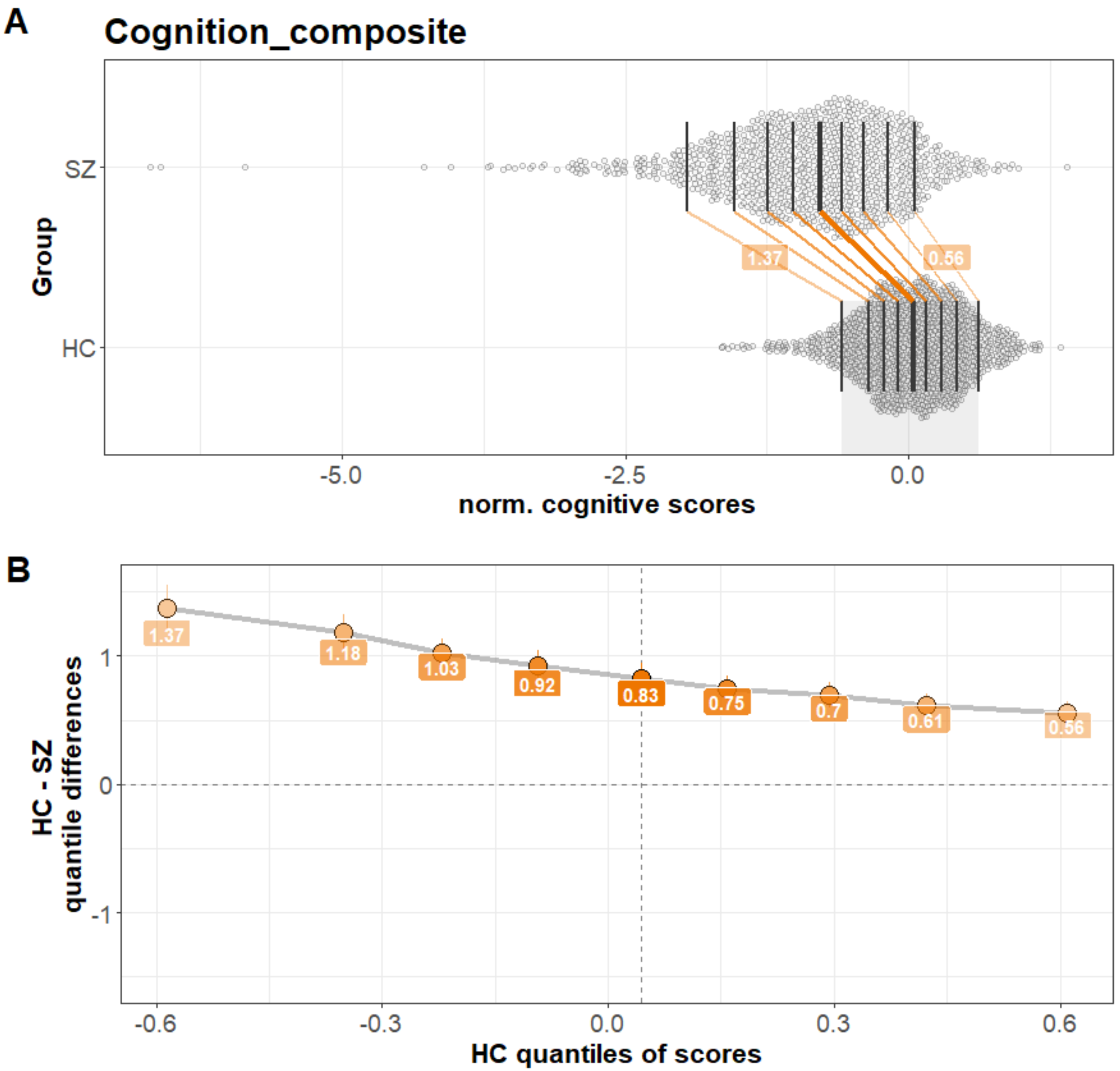

A
Cognition_composite
SZ
HC
Group
1.37
0.56
-5.0
-2.5
0.0
norm. cognitive scores
B
1.37
1.18
1.03
0.92
0.83
0.75
0.7
0.61
0.56
1
0
-1
HC - SZ
quantile differences
-0.6
-0.3
0.0
0.3
0.6
HC quantiles of scores

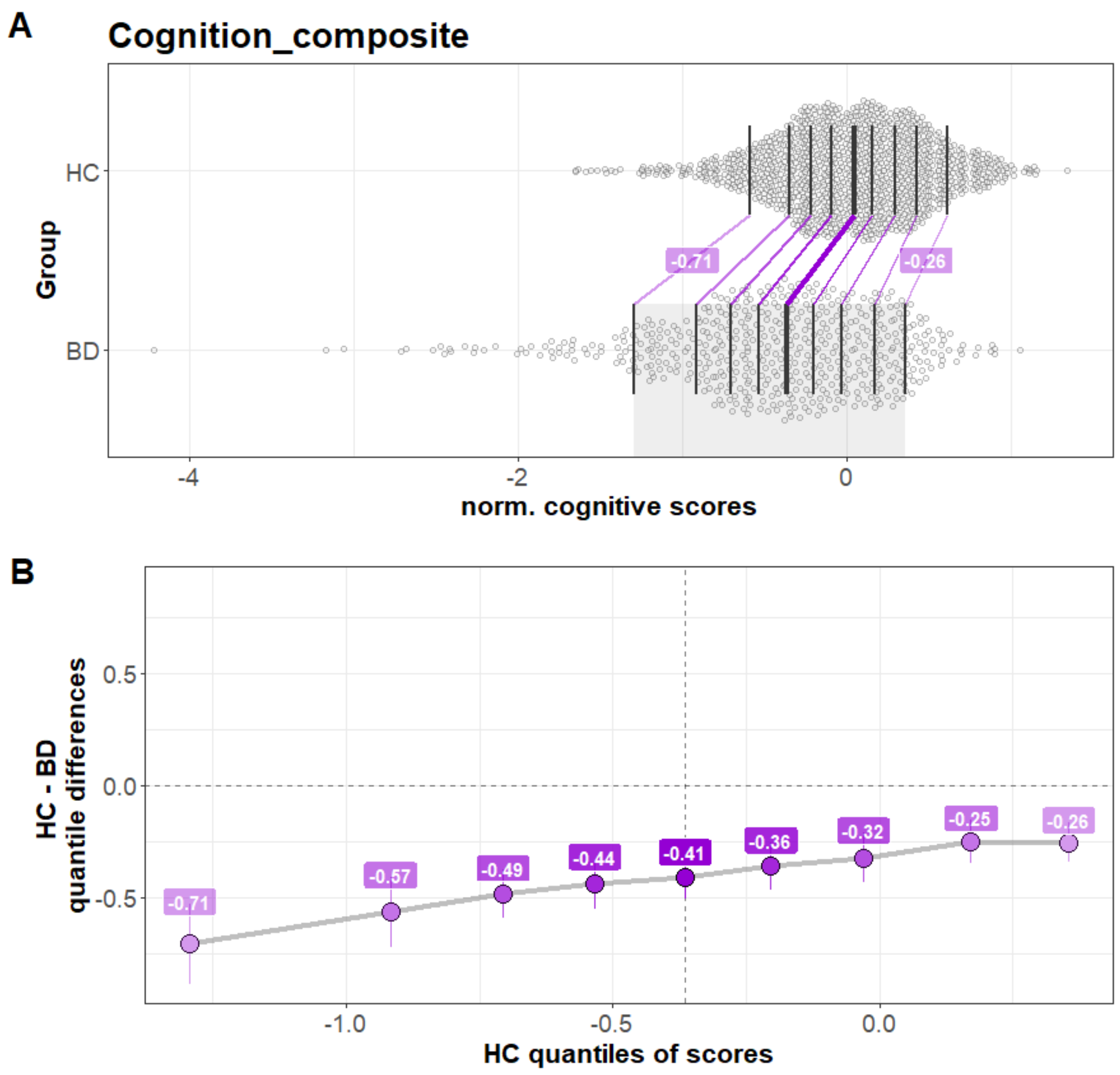
A
Cognition_composite
HC
BD
Group
-0.71
-0.26
-4
-2
0
norm. cognitive scores
B
HC - BD
quantile differences
0.5
0.0
-0.5
-0.71
-0.57
-0.49
-0.44
-0.41
-0.36
-0.32
-0.25
-0.26
-1.0
-0.5
0.0
HC quantiles of scores

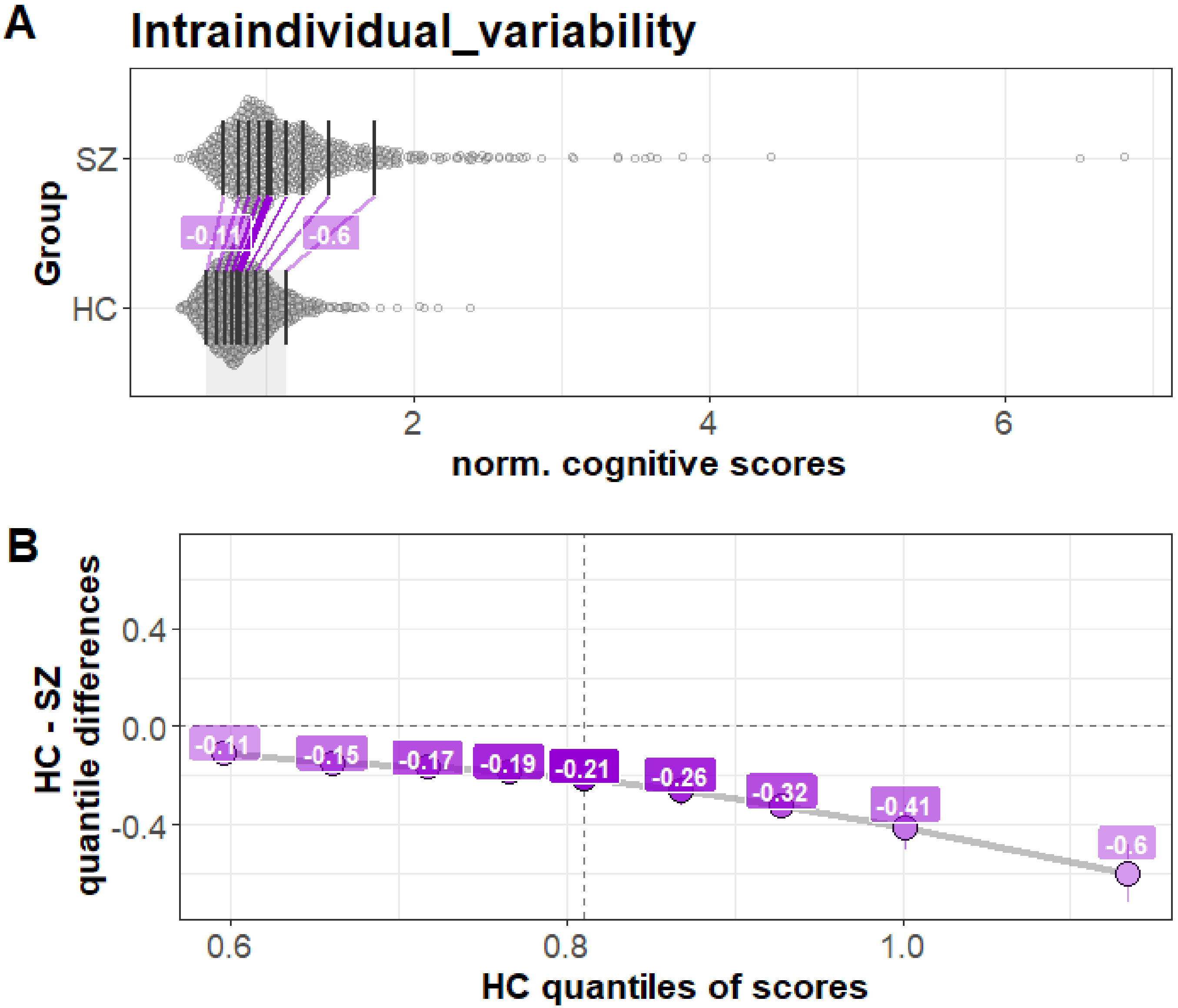

A
Intraindividual_variability
SZ
HC
Group
-0.11
-0.6
2
4
6
norm. cognitive scores
B
HC - SZ
quantile differences
0.4
0.0
-0.4
-0.11
-0.15
-0.17
-0.19
-0.21
-0.26
-0.32
-0.41
-0.6
0.6
0.8
1.0
HC quantiles of scores

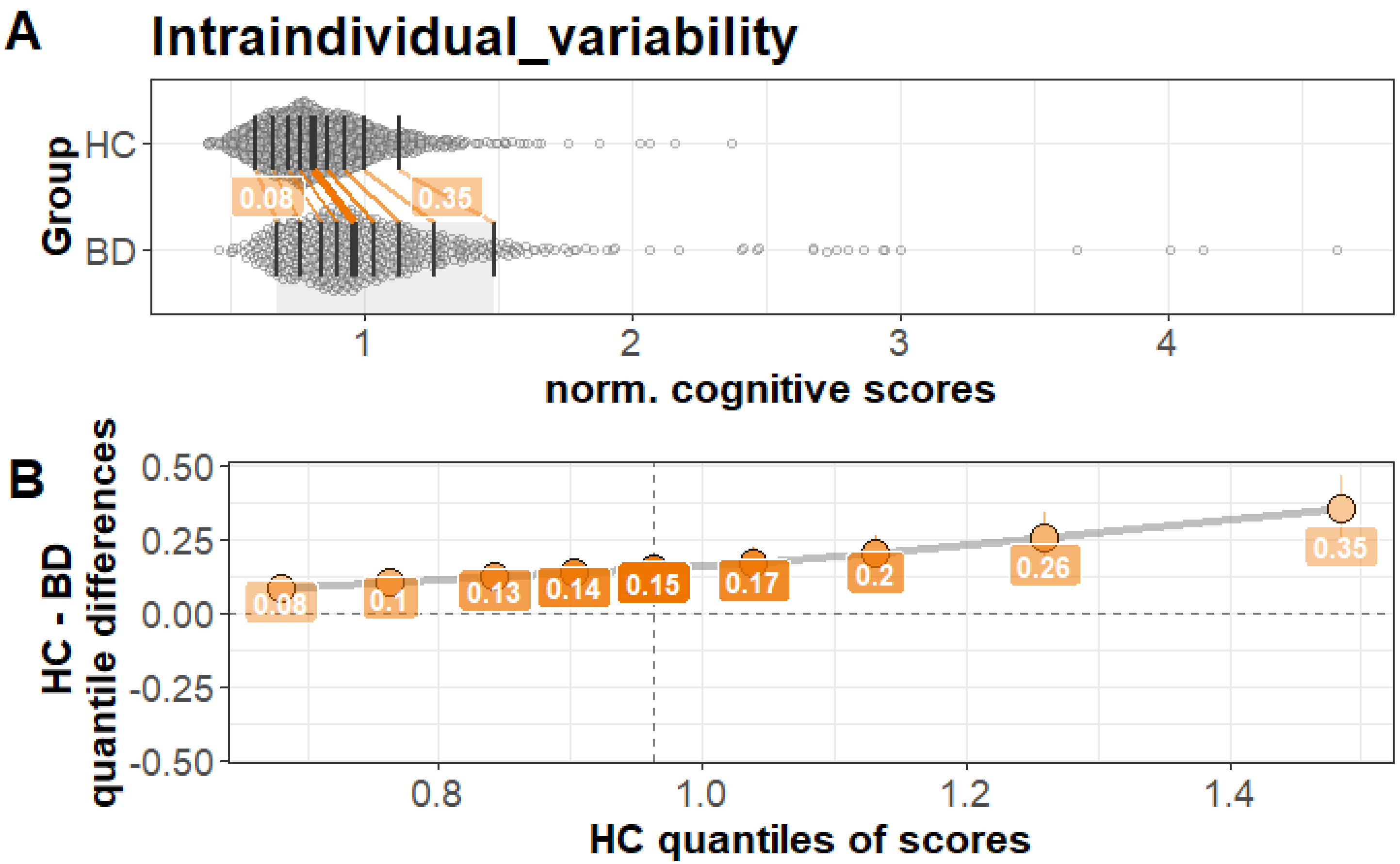

A
Intraindividual_variability
Group
HC
BD
0.08
0.35
1
2
3
4
norm. cognitive scores
B
HC - BD
quantile differences
0.50
0.25
0.00
-0.25
-0.50
0.08
0.1
0.13
0.14
0.15
0.17
0.2
0.26
0.35
0.8
1.0
1.2
1.4
HC quantiles of scores
